# Supplementary material for: Comparison of methods and resources for cell-cell communication inference from single-cell RNA-Seq data
Source: Nat Commun. 2022 Jun 9;13:3224. doi: 10.1038/s41467-022-30755-0 (PMC9184522; doi:10.1038/s41467-022-30755-0)
Supplement: Supplementary file 1 — Supplementary Info [file 41467_2022_30755_MOESM1_ESM.pdf]

Supplementary Information

Supplementary Figure S1. *Overlap between the resources represented as Jaccard indices between A) Interactions B) Receivers and C) Transmitters from different resources.*

A)

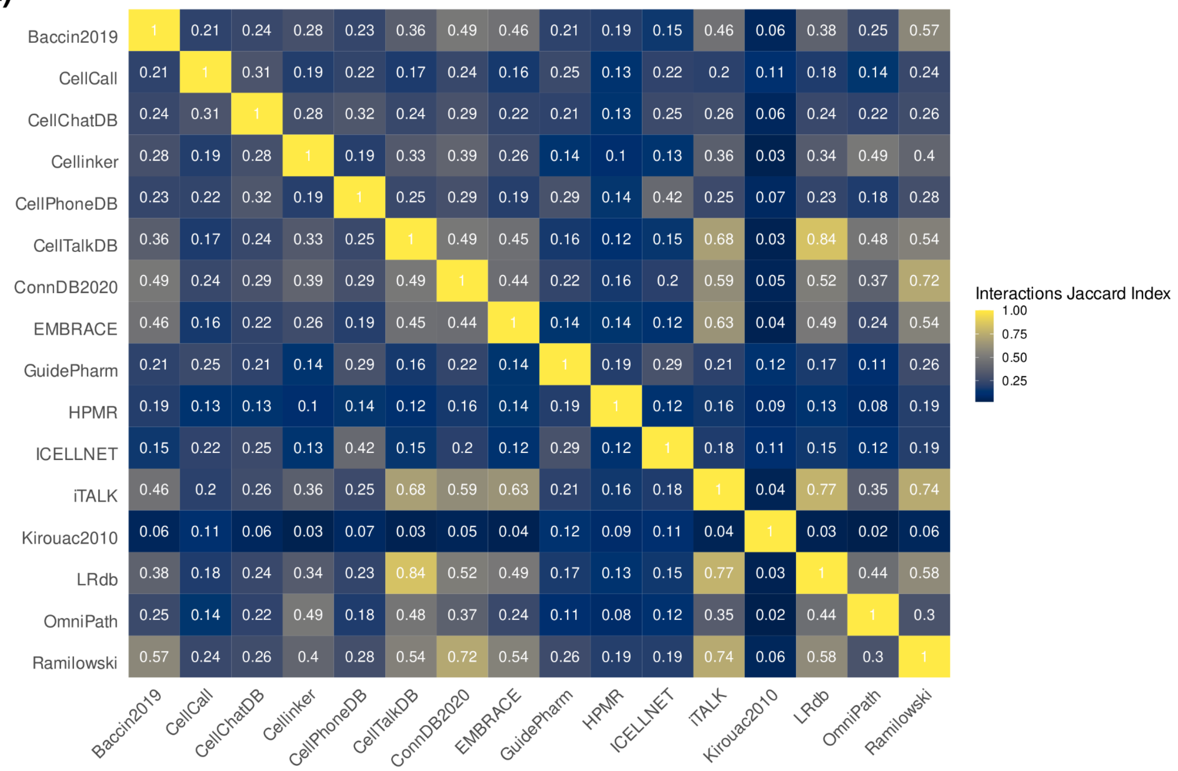

B)

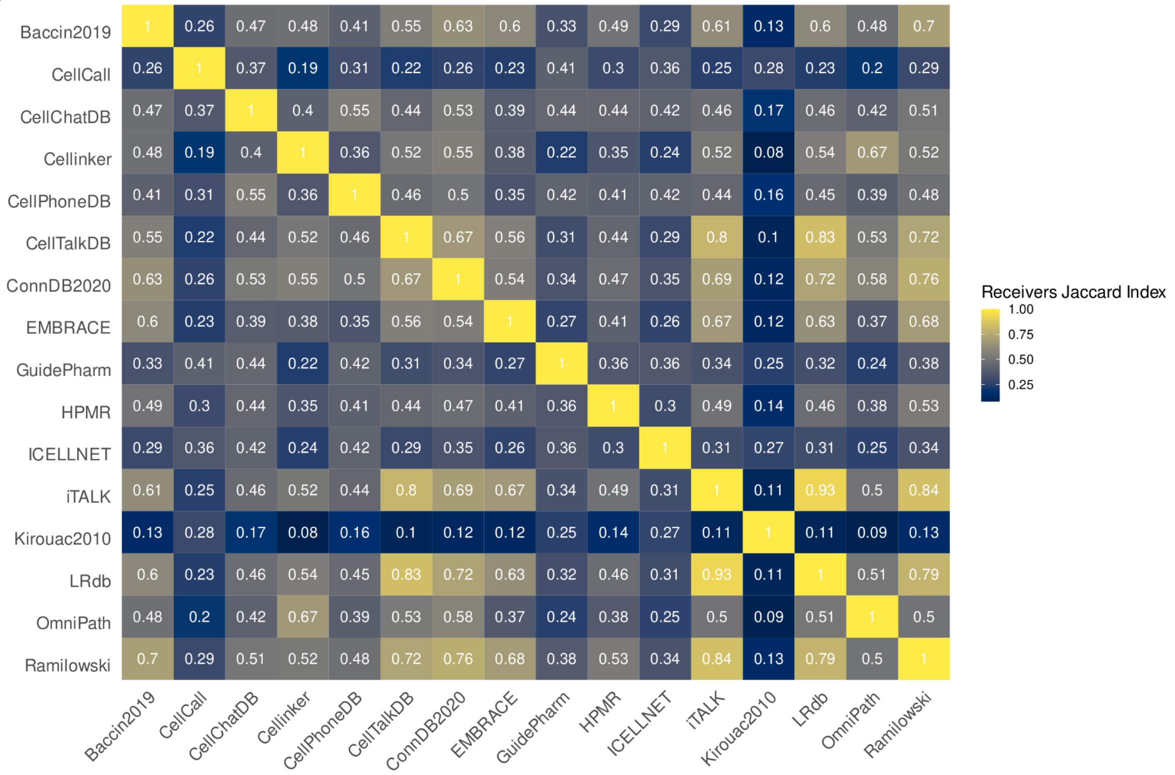
Receivers Jaccard Index
1.00
0.75
0.50
0.25
Baccin2019 1 0.26 0.47 0.48 0.41 0.55 0.63 0.6 0.33 0.49 0.29 0.61 0.13 0.6 0.48 0.7
CellCall 0.26 1 0.37 0.19 0.31 0.22 0.26 0.23 0.41 0.3 0.36 0.25 0.28 0.23 0.2 0.29
CellChatDB 0.47 0.37 1 0.4 0.55 0.44 0.53 0.39 0.44 0.44 0.42 0.46 0.17 0.46 0.42 0.51
Cellinker 0.48 0.19 0.4 1 0.36 0.52 0.55 0.38 0.22 0.35 0.24 0.52 0.08 0.54 0.67 0.52
CellPhoneDB 0.41 0.31 0.55 0.36 1 0.46 0.5 0.35 0.42 0.41 0.42 0.44 0.16 0.45 0.39 0.48
CellTalkDB 0.55 0.22 0.44 0.52 0.46 1 0.67 0.56 0.31 0.44 0.29 0.8 0.1 0.83 0.53 0.72
ConnDB2020 0.63 0.26 0.53 0.55 0.5 0.67 1 0.54 0.34 0.47 0.35 0.69 0.12 0.72 0.58 0.76
EMBRACE 0.6 0.23 0.39 0.38 0.35 0.56 0.54 1 0.27 0.41 0.26 0.67 0.12 0.63 0.37 0.68
GuidePharm 0.33 0.41 0.44 0.22 0.42 0.31 0.34 0.27 1 0.36 0.36 0.34 0.25 0.32 0.24 0.38
HPMR 0.49 0.3 0.44 0.35 0.41 0.44 0.47 0.41 0.36 1 0.3 0.49 0.14 0.46 0.38 0.53
ICELLNET 0.29 0.36 0.42 0.24 0.42 0.29 0.35 0.26 0.36 0.3 1 0.31 0.27 0.31 0.25 0.34
iTALK 0.61 0.25 0.46 0.52 0.44 0.8 0.69 0.67 0.34 0.49 0.31 1 0.11 0.93 0.5 0.84
Kirouac2010 0.13 0.28 0.17 0.08 0.16 0.1 0.12 0.12 0.25 0.14 0.27 0.11 1 0.11 0.09 0.13
LRdb 0.6 0.23 0.46 0.54 0.45 0.83 0.72 0.63 0.32 0.46 0.31 0.93 0.11 1 0.51 0.79
OmniPath 0.48 0.2 0.42 0.67 0.39 0.53 0.58 0.37 0.24 0.38 0.25 0.5 0.09 0.51 1 0.5
Ramilowski 0.7 0.29 0.51 0.52 0.48 0.72 0.76 0.68 0.38 0.53 0.34 0.84 0.13 0.79 0.5 1
Baccin2019 CellCall CellChatDB Cellinker CellPhoneDB CellTalkDB ConnDB2020 EMBRACE GuidePharm HPMR ICELLNET iTALK Kirouac2010 LRdb OmniPath Ramilowski

C)

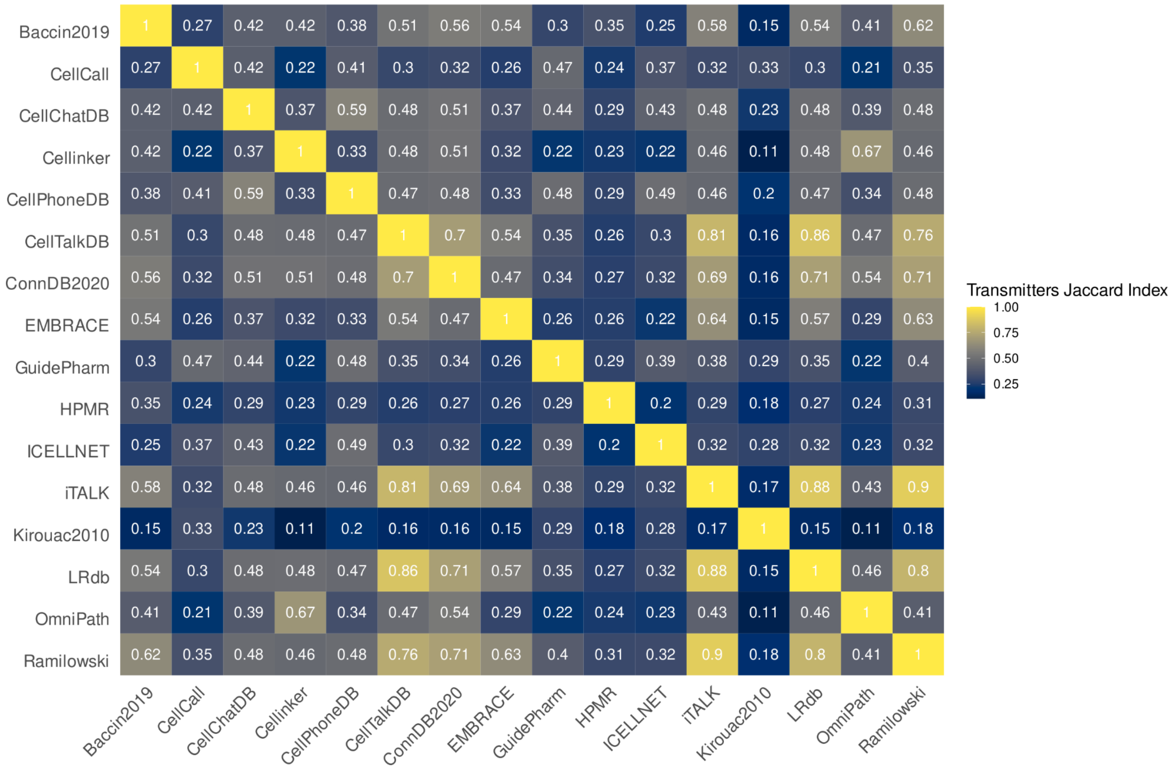
Transmitters Jaccard Index
1.00
0.75
0.50
0.25
Baccin2019 1 0.27 0.42 0.42 0.38 0.51 0.56 0.54 0.3 0.35 0.25 0.58 0.15 0.54 0.41 0.62
CellCall 0.27 1 0.42 0.22 0.41 0.3 0.32 0.26 0.47 0.24 0.37 0.32 0.33 0.3 0.21 0.35
CellChatDB 0.42 0.42 1 0.37 0.59 0.48 0.51 0.37 0.44 0.29 0.43 0.48 0.23 0.48 0.39 0.48
Cellinker 0.42 0.22 0.37 1 0.33 0.48 0.51 0.32 0.22 0.23 0.22 0.46 0.11 0.48 0.67 0.46
CellPhoneDB 0.38 0.41 0.59 0.33 1 0.47 0.48 0.33 0.48 0.29 0.49 0.46 0.2 0.47 0.34 0.48
CellTalkDB 0.51 0.3 0.48 0.48 0.47 1 0.7 0.54 0.35 0.26 0.3 0.81 0.16 0.86 0.47 0.76
ConnDB2020 0.56 0.32 0.51 0.51 0.48 0.7 1 0.47 0.34 0.27 0.32 0.69 0.16 0.71 0.54 0.71
EMBRACE 0.54 0.26 0.37 0.32 0.33 0.54 0.47 1 0.26 0.26 0.22 0.64 0.15 0.57 0.29 0.63
GuidePharm 0.3 0.47 0.44 0.22 0.48 0.35 0.34 0.26 1 0.29 0.39 0.38 0.29 0.35 0.22 0.4
HPMR 0.35 0.24 0.29 0.23 0.29 0.26 0.27 0.26 0.29 1 0.2 0.29 0.18 0.27 0.24 0.31
ICELLNET 0.25 0.37 0.43 0.22 0.49 0.3 0.32 0.22 0.39 0.2 1 0.32 0.28 0.32 0.23 0.32
iTALK 0.58 0.32 0.48 0.46 0.46 0.81 0.69 0.64 0.38 0.29 0.32 1 0.17 0.88 0.43 0.9
Kirouac2010 0.15 0.33 0.23 0.11 0.2 0.16 0.16 0.15 0.29 0.18 0.28 0.17 1 0.15 0.11 0.18
LRdb 0.54 0.3 0.48 0.48 0.47 0.86 0.71 0.57 0.35 0.27 0.32 0.88 0.15 1 0.46 0.8
OmniPath 0.41 0.21 0.39 0.67 0.34 0.47 0.54 0.29 0.22 0.24 0.23 0.43 0.11 0.46 1 0.41
Ramilowski 0.62 0.35 0.48 0.46 0.48 0.76 0.71 0.63 0.4 0.31 0.32 0.9 0.18 0.8 0.41 1
Baccin2019 CellCall CellChatDB Cellinker CellPhoneDB CellTalkDB ConnDB2020 EMBRACE GuidePharm HPMR ICELLNET iTALK Kirouac2010 LRdb OmniPath Ramilowski

Supplementary Figure S2. *Asymmetric overlap between the resources.* ***A)*** *Interactions* ***B)*** *Receivers,* ***C)*** *Transmitters, and* ***D)*** *Curated interactions present in each resource when taken from the rest of the resources. Note these plots are asymmetric and represent the % of interactions from the resources on the X axis found in each resource on the Y axis.*

A)

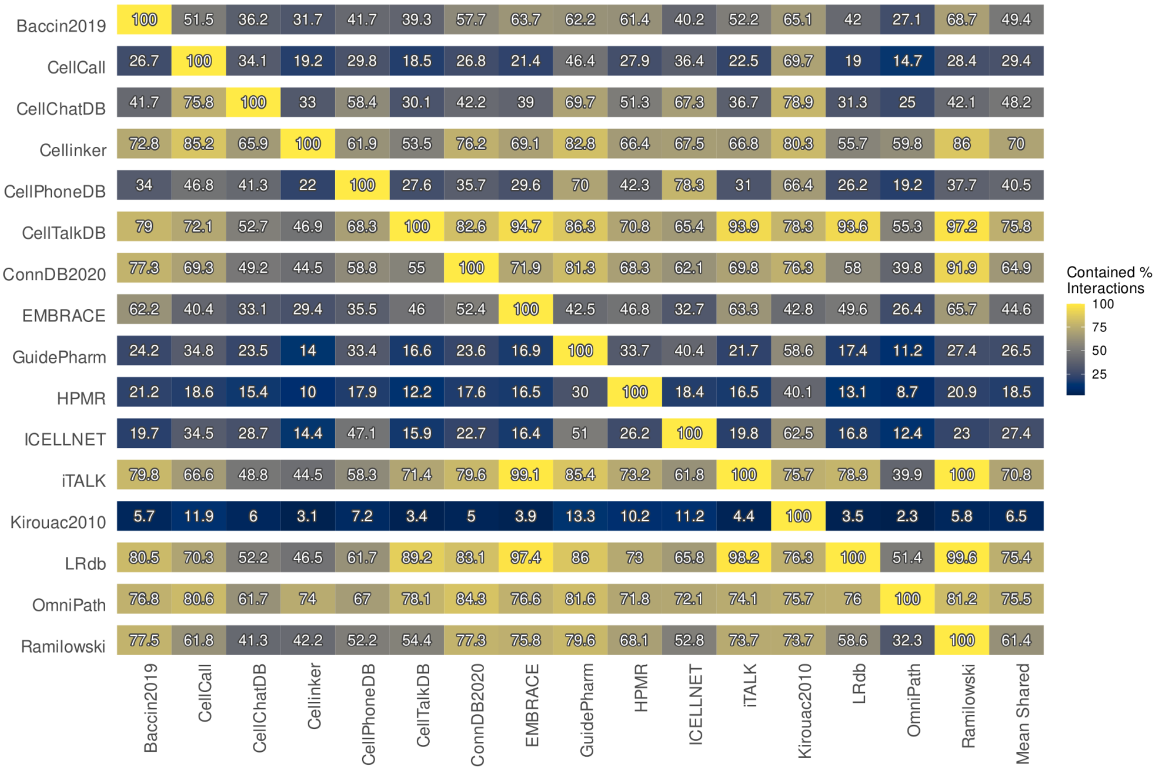

B)

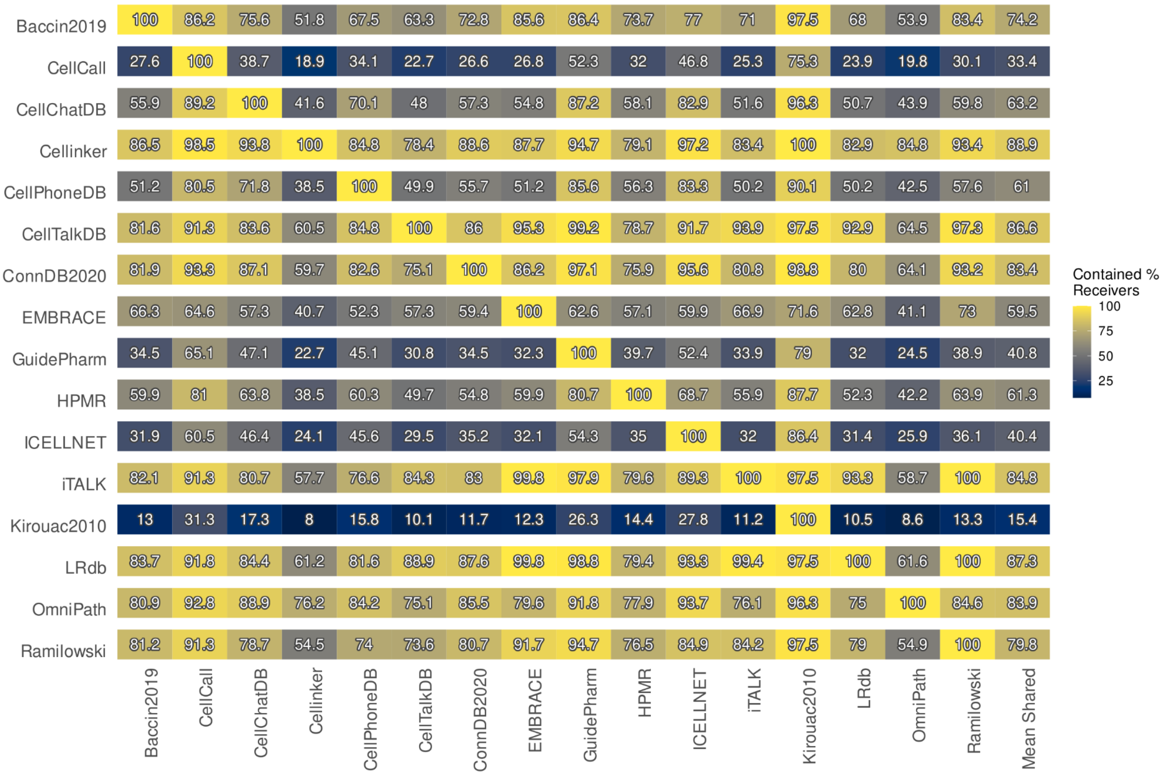

C)

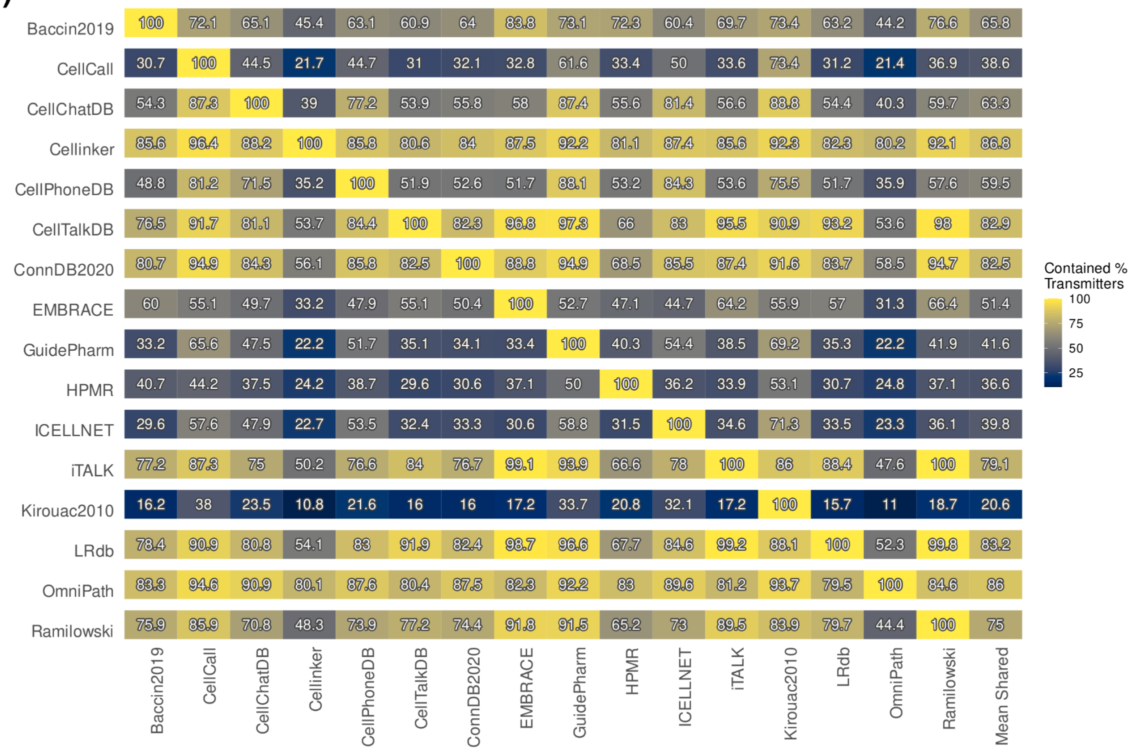
Baccin2019 100 72.1 65.1 45.4 63.1 60.9 64 83.8 73.1 72.3 60.4 69.7 73.4 63.2 44.2 76.6 65.8
CellCall 30.7 100 44.5 21.7 44.7 31 32.1 32.8 61.6 33.4 50 33.6 73.4 31.2 21.4 36.9 38.6
CellChatDB 54.3 87.3 100 39 77.2 53.9 55.8 58 87.4 55.6 81.4 56.6 88.8 54.4 40.3 59.7 63.3
Cellinker 85.6 96.4 88.2 100 85.8 80.6 84 87.5 92.2 81.1 87.4 85.6 92.3 82.3 80.2 92.1 86.8
CellPhoneDB 48.8 81.2 71.5 35.2 100 51.9 52.6 51.7 88.1 53.2 84.3 53.6 75.5 51.7 35.9 57.6 59.5
CellTalkDB 76.5 91.7 81.1 53.7 84.4 100 82.3 96.8 97.3 66 83 95.5 90.9 93.2 53.6 98 82.9
ConnDB2020 80.7 94.9 84.3 56.1 85.8 82.5 100 88.8 94.9 68.5 85.5 87.4 91.6 83.7 58.5 94.7 82.5
EMBRACE 60 55.1 49.7 33.2 47.9 55.1 50.4 100 52.7 47.1 44.7 64.2 55.9 57 31.3 66.4 51.4
GuidePharm 33.2 65.6 47.5 22.2 51.7 35.1 34.1 33.4 100 40.3 54.4 38.5 69.2 35.3 22.2 41.9 41.6
HPMR 40.7 44.2 37.5 24.2 38.7 29.6 30.6 37.1 50 100 36.2 33.9 53.1 30.7 24.8 37.1 36.6
ICELLNET 29.6 57.6 47.9 22.7 53.5 32.4 33.3 30.6 58.8 31.5 100 34.6 71.3 33.5 23.3 36.1 39.8
iTALK 77.2 87.3 75 50.2 76.6 84 76.7 99.1 93.9 66.6 78 100 86 88.4 47.6 100 79.1
Kirouac2010 16.2 38 23.5 10.8 21.6 16 16 17.2 33.7 20.8 32.1 17.2 100 15.7 11 18.7 20.6
LRdb 78.4 90.9 80.8 54.1 83 91.9 82.4 98.7 96.6 67.7 84.6 99.2 88.1 100 52.3 99.8 83.2
OmniPath 83.3 94.6 90.9 80.1 87.6 80.4 87.5 82.3 92.2 83 89.6 81.2 93.7 79.5 100 84.6 86
Ramilowski 75.9 85.9 70.8 48.3 73.9 77.2 74.4 91.8 91.5 65.2 73 89.5 83.9 79.7 44.4 100 75
Baccin2019 CellCall CellChatDB Cellinker CellPhoneDB CellTalkDB ConnDB2020 EMBRACE GuidePharm HPMR ICELLNET iTALK Kirouac2010 LRdb OmniPath Ramilowski Mean Shared
Contained %
Transmitters
100
75
50
25

D)

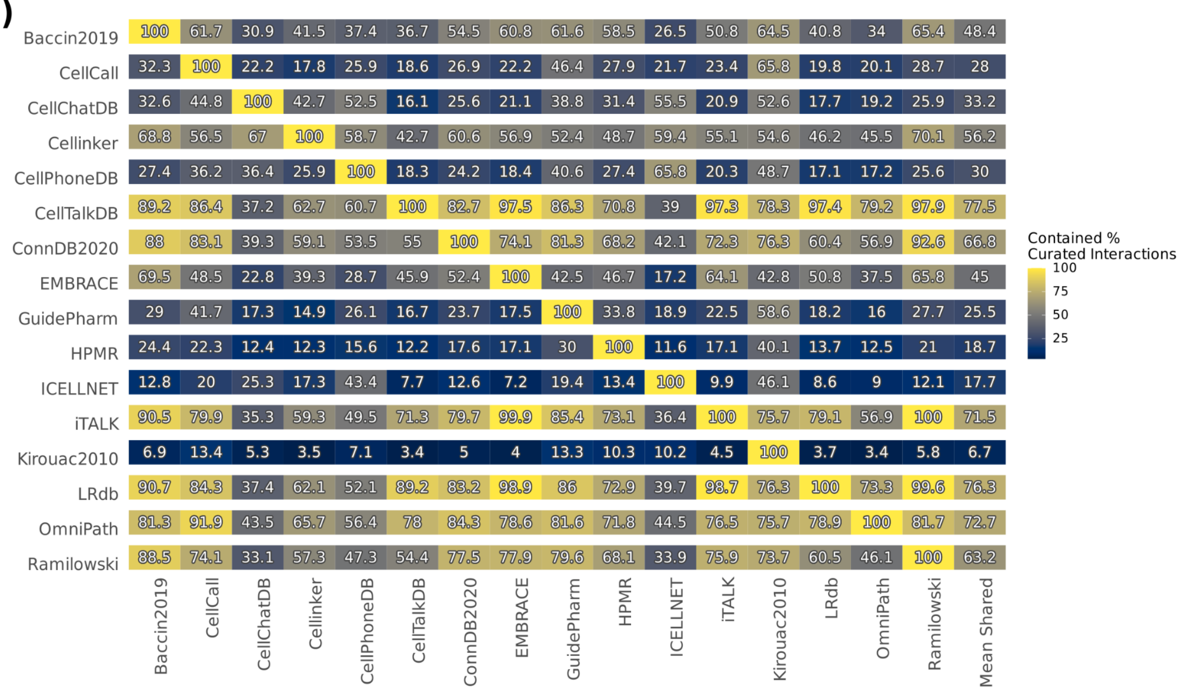
Baccin2019 100 61.7 30.9 41.5 37.4 36.7 54.5 60.8 61.6 58.5 26.5 50.8 64.5 40.8 34 65.4 48.4
CellCall 32.3 100 22.2 17.8 25.9 18.6 26.9 22.2 46.4 27.9 21.7 23.4 65.8 19.8 20.1 28.7 28
CellChatDB 32.6 44.8 100 42.7 52.5 16.1 25.6 21.1 38.8 31.4 55.5 20.9 52.6 17.7 19.2 25.9 33.2
Cellinker 68.8 56.5 67 100 58.7 42.7 60.6 56.9 52.4 48.7 59.4 55.1 54.6 46.2 45.5 70.1 56.2
CellPhoneDB 27.4 36.2 36.4 25.9 100 18.3 24.2 18.4 40.6 27.4 65.8 20.3 48.7 17.1 17.2 25.6 30
CellTalkDB 89.2 86.4 37.2 62.7 60.7 100 82.7 97.5 86.3 70.8 39 97.3 78.3 97.4 79.2 97.9 77.5
ConnDB2020 88 83.1 39.3 59.1 53.5 55 100 74.1 81.3 68.2 42.1 72.3 76.3 60.4 56.9 92.6 66.8
EMBRACE 69.5 48.5 22.8 39.3 28.7 45.9 52.4 100 42.5 46.7 17.2 64.1 42.8 50.8 37.5 65.8 45
GuidePharm 29 41.7 17.3 14.9 26.1 16.7 23.7 17.5 100 33.8 18.9 22.5 58.6 18.2 16 27.7 25.5
HPMR 24.4 22.3 12.4 12.3 15.6 12.2 17.6 17.1 30 100 11.6 17.1 40.1 13.7 12.5 21 18.7
ICELLNET 12.8 20 25.3 17.3 43.4 7.7 12.6 7.2 19.4 13.4 100 9.9 46.1 8.6 9 12.1 17.7
iTALK 90.5 79.9 35.3 59.3 49.5 71.3 79.7 99.9 85.4 73.1 36.4 100 75.7 79.1 56.9 100 71.5
Kirouac2010 6.9 13.4 5.3 3.5 7.1 3.4 5 4 13.3 10.3 10.2 4.5 100 3.7 3.4 5.8 6.7
LRdb 90.7 84.3 37.4 62.1 52.1 89.2 83.2 98.9 86 72.9 39.7 98.7 76.3 100 73.3 99.6 76.3
OmniPath 81.3 91.9 43.5 65.7 56.4 78 84.3 78.6 81.6 71.8 44.5 76.5 75.7 78.9 100 81.7 72.7
Ramilowski 88.5 74.1 33.1 57.3 47.3 54.4 77.5 77.9 79.6 68.1 33.9 75.9 73.7 60.5 46.1 100 63.2
Baccin2019 CellCall CellChatDB Cellinker CellPhoneDB CellTalkDB ConnDB2020 EMBRACE GuidePharm HPMR ICELLNET iTALK Kirouac2010 LRdb OmniPath Ramilowski Mean Shared
Contained %
Curated Interactions
100
75
50
25

Supplementary Figure S3. *SignaLink pathway distributions across CCC resources. Enrichment Scores (A-C), Percentage Proportions (D-F), and Number of matches (G-I) to the SignaLink database per resource for Interactions, Receivers and Transmitters from different CCC resources. Fisher's exact test was used to estimate the differentially-represented categories. Differentially represented (absolute(log2(Odds ratio)) > 1) categories were marked according to FDR-corrected p-values =< 0.05 (diamond; ◊), 0.01 (triangle; △), and 0.001 (8-pointed asterisk; ✳).*

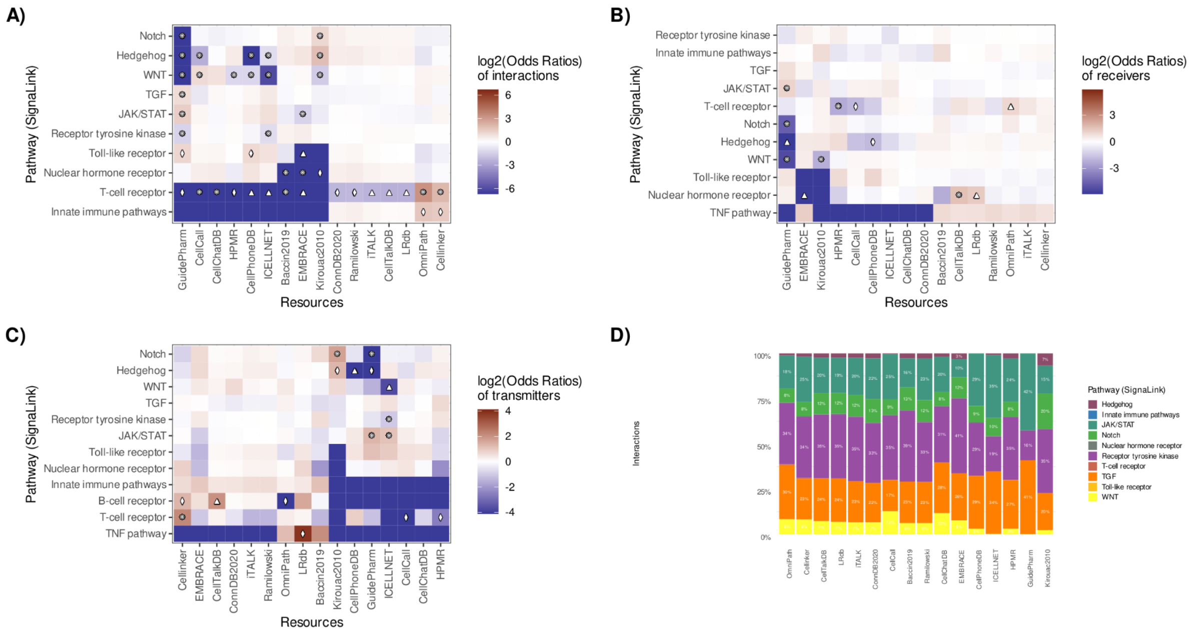

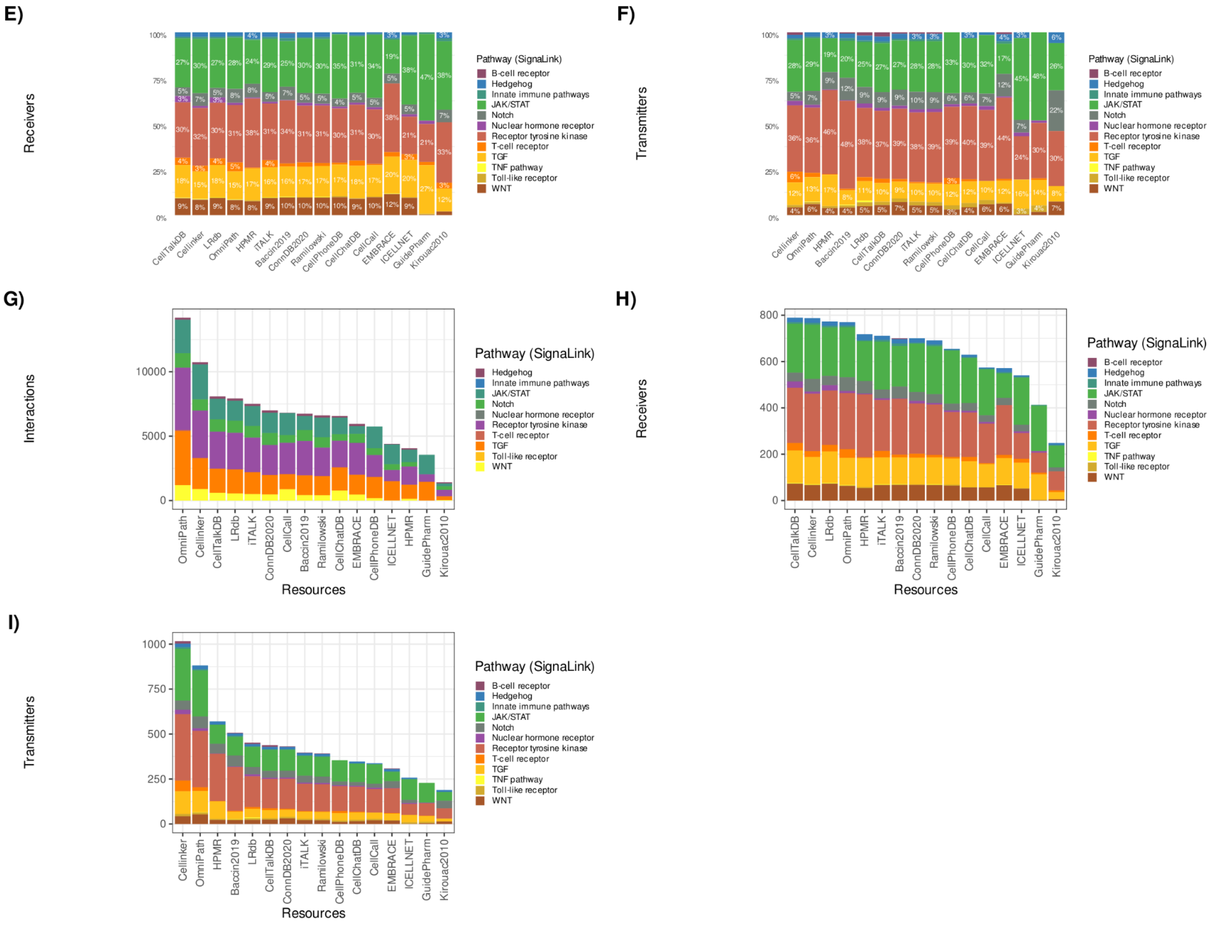
E)
Receivers
Pathway (SignaLink)
B-cell receptor
Hedgehog
Innate immune pathways
JAK/STAT
Notch
Nuclear hormone receptor
Receptor tyrosine kinase
T-cell receptor
TGF
TNF pathway
Toll-like receptor
WNT
F)
Transmitters
G)
Interactions
Resources
H)
Receivers
Resources
I)
Transmitters
Resources
OmniPath
Cellinker
CellTalkDB
LRdb
iTALK
ConnDB2020
CellCall
Baccin2019
Ramilowski
CellChatDB
EMBRACE
CellPhoneDB
ICELLNET
HPMR
GuidePharm
Kirouac2010

Supplementary Figure S4. *NetPath pathway distributions across CCC resources. Enrichment Scores (A-C), Percentage Proportions (D-F), and Number of matches (G-I) to the NetPath database per resource for Interactions, Receivers and Transmitters from different CCC resources. Fisher's exact test was used to estimate the differentially-represented categories. Differentially represented (absolute(log2(Odds ratio)) > 1) categories were marked according to FDR-corrected p-values =< 0.05 (diamond; ◇), 0.01 (triangle; △), and 0.001 (8-pointed asterisk; ✻).*

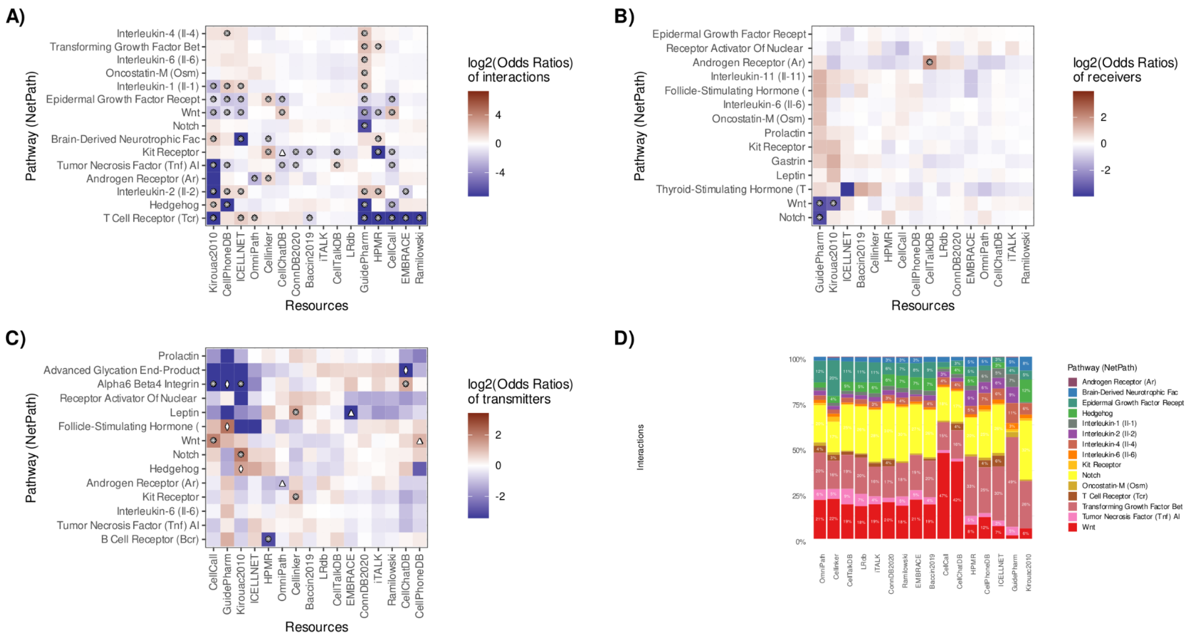

E)

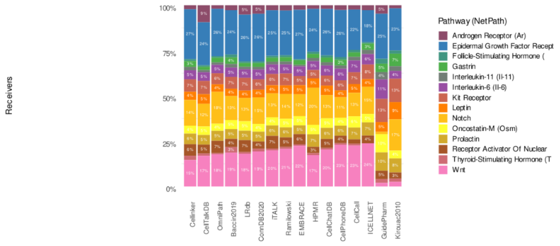
Receivers
Pathway (NetPath)
Androgen Receptor (Ar)
Epidermal Growth Factor Recept
Follicle-Stimulating Hormone (
Gastrin
Interleukin-11 (Il-11)
Interleukin-6 (Il-6)
Kit Receptor
Leptin
Notch
Oncostatin-M (Osm)
Prolactin
Receptor Activator Of Nuclear
Thyroid-Stimulating Hormone (T
Wnt

F)

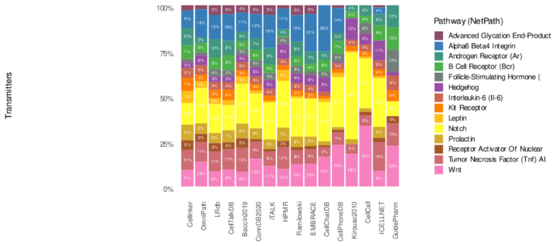
Transmitters
Pathway (NetPath)
Advanced Glycation End-Product
Alpha6 Beta4 Integrin
Androgen Receptor (Ar)
B Cell Receptor (Bcr)
Follicle-Stimulating Hormone (
Hedgehog
Interleukin-6 (Il-6)
Kit Receptor
Leptin
Notch
Prolactin
Receptor Activator Of Nuclear
Tumor Necrosis Factor (Tnf) Al
Wnt

G)

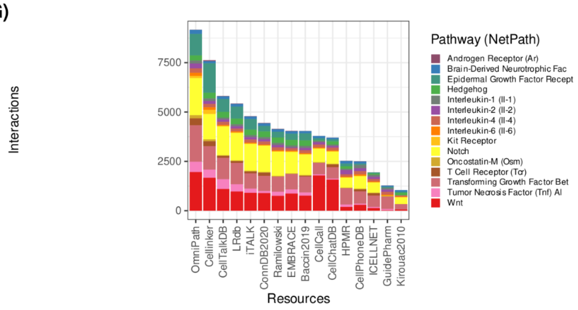
Interactions
Resources
Pathway (NetPath)
Androgen Receptor (Ar)
Brain-Derived Neurotrophic Fac
Epidermal Growth Factor Recept
Hedgehog
Interleukin-1 (Il-1)
Interleukin-2 (Il-2)
Interleukin-4 (Il-4)
Interleukin-6 (Il-6)
Kit Receptor
Notch
Oncostatin-M (Osm)
T Cell Receptor (Tcr)
Transforming Growth Factor Bet
Tumor Necrosis Factor (Tnf) Al
Wnt

H)

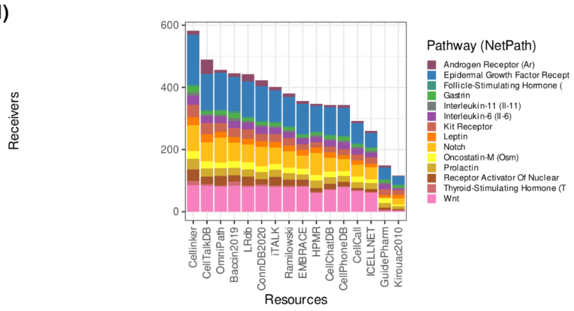
Receivers
Resources
Pathway (NetPath)
Androgen Receptor (Ar)
Epidermal Growth Factor Recept
Follicle-Stimulating Hormone (
Gastrin
Interleukin-11 (Il-11)
Interleukin-6 (Il-6)
Kit Receptor
Leptin
Notch
Oncostatin-M (Osm)
Prolactin
Receptor Activator Of Nuclear
Thyroid-Stimulating Hormone (T
Wnt

I)

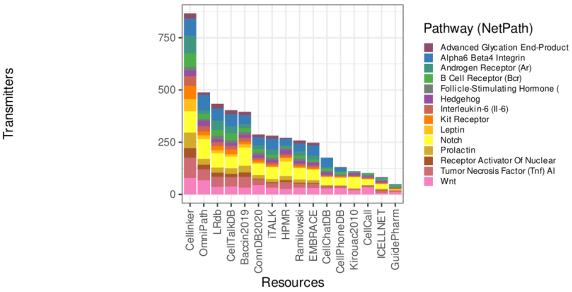
Transmitters
Resources
Pathway (NetPath)
Advanced Glycation End-Product
Alpha6 Beta4 Integrin
Androgen Receptor (Ar)
B Cell Receptor (Bcr)
Follicle-Stimulating Hormone (
Hedgehog
Interleukin-6 (Il-6)
Kit Receptor
Leptin
Notch
Prolactin
Receptor Activator Of Nuclear
Tumor Necrosis Factor (Tnf) Al
Wnt

Supplementary Figure S5. *CancerSEA cancer genes-set distributions across CCC resources. Enrichment Scores (A-C), Percentage Proportions (D-F), and Number of matches (G-I) to the CancerSEA database per resource for Interactions, Receivers and Transmitters from different CCC resources. Fisher's exact test was used to estimate the differentially-represented categories. Differentially represented (absolute(log2(Odds ratio)) > 1) categories were marked according to FDR-corrected p-values =< 0.05 (diamond; ◊), 0.01 (triangle; △), and 0.001 (8-pointed asterisk; ✳).*

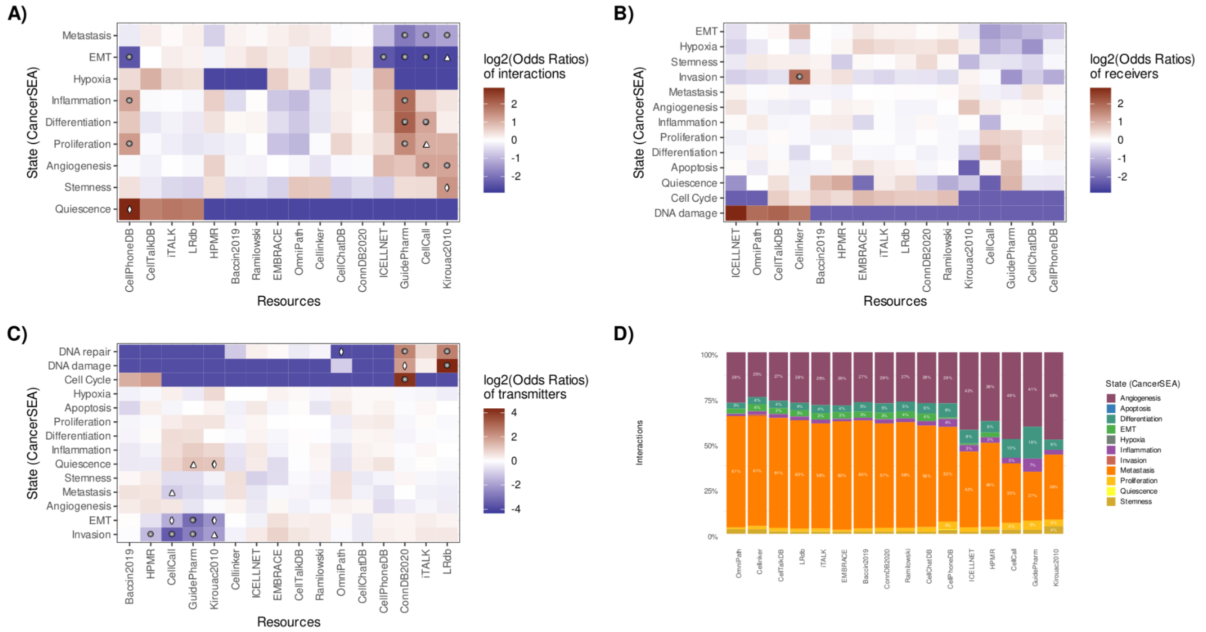

E)

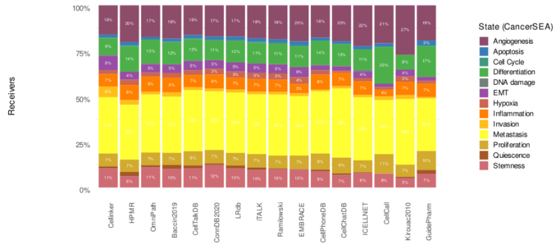
Receivers
State (CancerSEA)
Angiogenesis
Apoptosis
Cell Cycle
Differentiation
DNA damage
EMT
Hypoxia
Inflammation
Invasion
Metastasis
Proliferation
Quiescence
Stemness
Cellinker
HPMR
OmniPath
Baccin2019
CellTalkDB
ConnDB2020
LRdb
iTALK
Ramilowski
EMBRACE
CellPhoneDB
CellChatDB
ICELLNET
CellCall
Kirouac2010
GuidePharm

F)

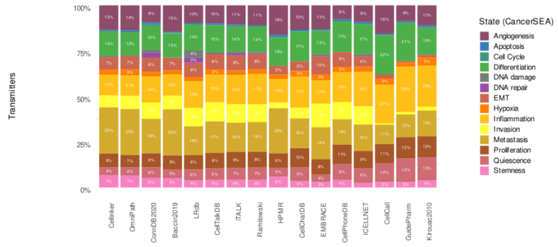
Transmitters
State (CancerSEA)
Angiogenesis
Apoptosis
Cell Cycle
Differentiation
DNA damage
DNA repair
EMT
Hypoxia
Inflammation
Invasion
Metastasis
Proliferation
Quiescence
Stemness
Cellinker
OmniPath
ConnDB2020
Baccin2019
LRdb
CellTalkDB
iTALK
Ramilowski
HPMR
CellChatDB
EMBRACE
CellPhoneDB
ICELLNET
CellCall
GuidePharm
Kirouac2010

G)

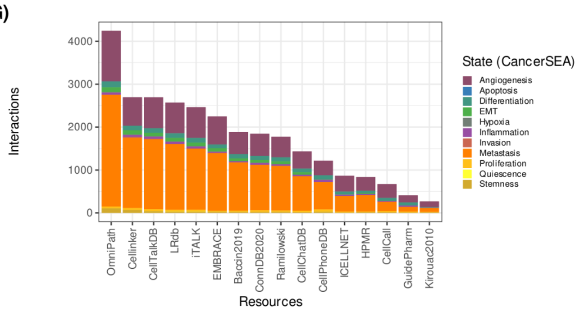
Interactions
Resources
State (CancerSEA)
Angiogenesis
Apoptosis
Differentiation
EMT
Hypoxia
Inflammation
Invasion
Metastasis
Proliferation
Quiescence
Stemness
OmniPath
Cellinker
CellTalkDB
LRdb
iTALK
EMBRACE
Baccin2019
ConnDB2020
Ramilowski
CellChatDB
CellPhoneDB
ICELLNET
HPMR
CellCall
GuidePharm
Kirouac2010

H)

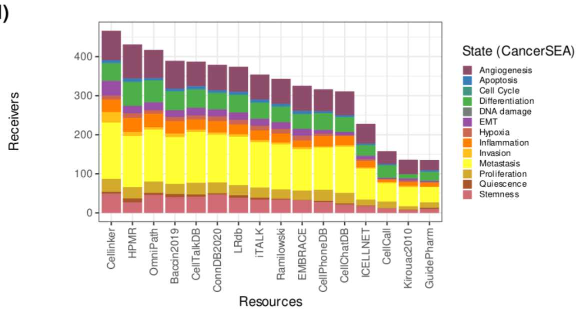
Receivers
Resources
State (CancerSEA)
Angiogenesis
Apoptosis
Cell Cycle
Differentiation
DNA damage
EMT
Hypoxia
Inflammation
Invasion
Metastasis
Proliferation
Quiescence
Stemness
Cellinker
HPMR
OmniPath
Baccin2019
CellTalkDB
ConnDB2020
LRdb
iTALK
Ramilowski
EMBRACE
CellPhoneDB
CellChatDB
ICELLNET
CellCall
Kirouac2010
GuidePharm

I)

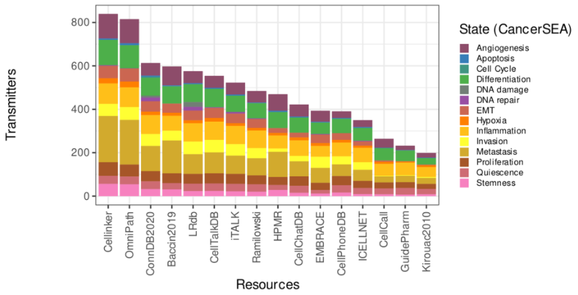
Transmitters
Resources
State (CancerSEA)
Angiogenesis
Apoptosis
Cell Cycle
Differentiation
DNA damage
DNA repair
EMT
Hypoxia
Inflammation
Invasion
Metastasis
Proliferation
Quiescence
Stemness
Cellinker
OmniPath
ConnDB2020
Baccin2019
LRdb
CellTalkDB
iTALK
Ramilowski
HPMR
CellChatDB
EMBRACE
CellPhoneDB
ICELLNET
CellCall
GuidePharm
Kirouac2010

Supplementary Figure S6. *Localisation category distributions across CCC resources. Enrichment Scores (A-C), Percentage Proportions (D-F), and Number of matches (G-I) to the subcellular protein localisation categories from the OmniPath database for Interactions, Receivers and Transmitters from different CCC resources. '**T**', '**P**', and '**S**' stand for transmembrane, peripheral, and secreted, respectively. Fisher's exact test was used to estimate the differentially-represented categories. Differentially represented (absolute(log2(Odds ratio)) > 1) categories were marked according to FDR-corrected p-values =< 0.05 (diamond; ◇), 0.01 (triangle; △), and 0.001 (8-pointed asterisk; ❋).*

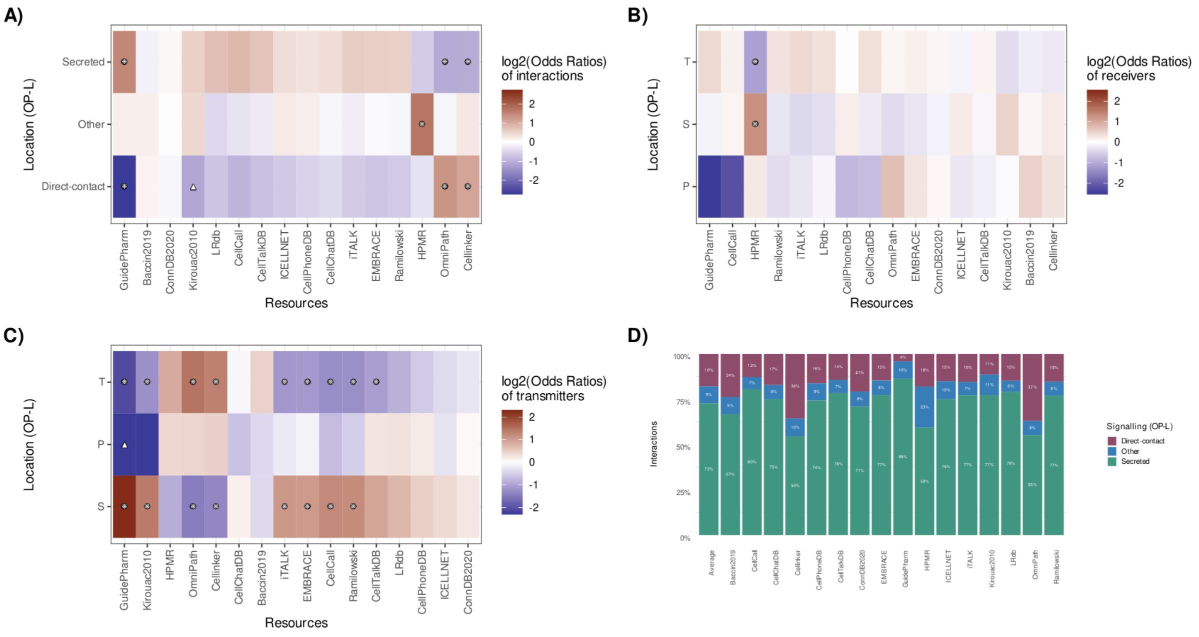

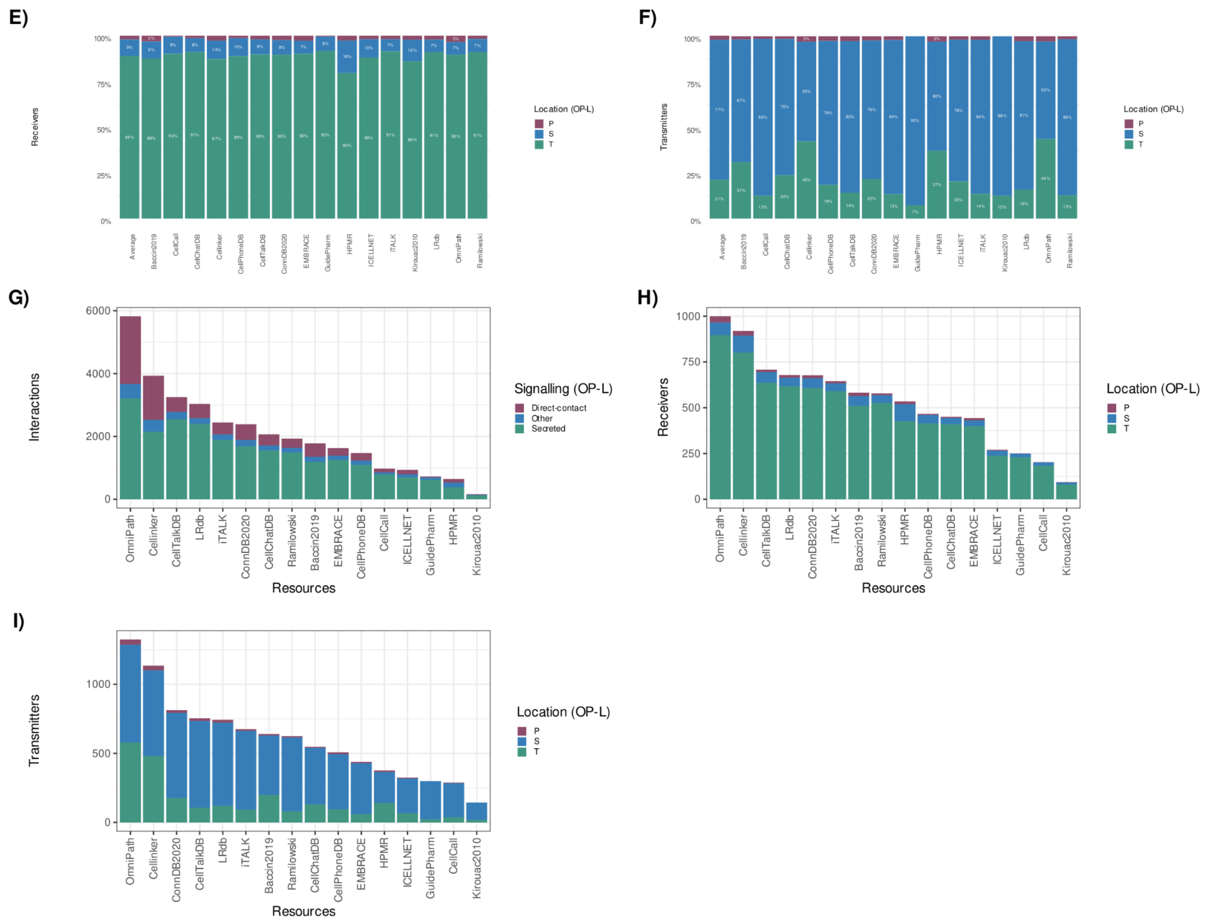

E)
Receivers
Location (OP-L)
P
S
T
Average
Baccin2019
CellCall
CellChatDB
Cellinker
CellPhoneDB
CellTalkDB
ConnDB2020
EMBRACE
GuidePharm
HPMR
ICELLNET
iTALK
Kirouac2010
LRdb
OmniPath
Ramilowski
F)
Transmitters
G)
Interactions
Signalling (OP-L)
Direct-contact
Other
Secreted
Resources
H)
Receivers
Location (OP-L)
Resources
I)
Transmitters
Location (OP-L)
Resources

Supplementary Figure S7. *Organ-enriched protein distributions across CCC resources. Enrichment Scores (A-C), Percentage Proportions (D-F), and Number of matches (G-I) to the 15 largest categories of organ-enriched proteins in the HPA database per resource for Interactions, Receivers and Transmitters from different CCC resources. Fisher's exact test was used to estimate the differentially-represented categories. Differentially represented (absolute(log2(Odds ratio)) > 1) categories were marked according to FDR-corrected p-values =< 0.05 (diamond; ◇), 0.01 (triangle; △), and 0.001 (8-pointed asterisk; ✻).*

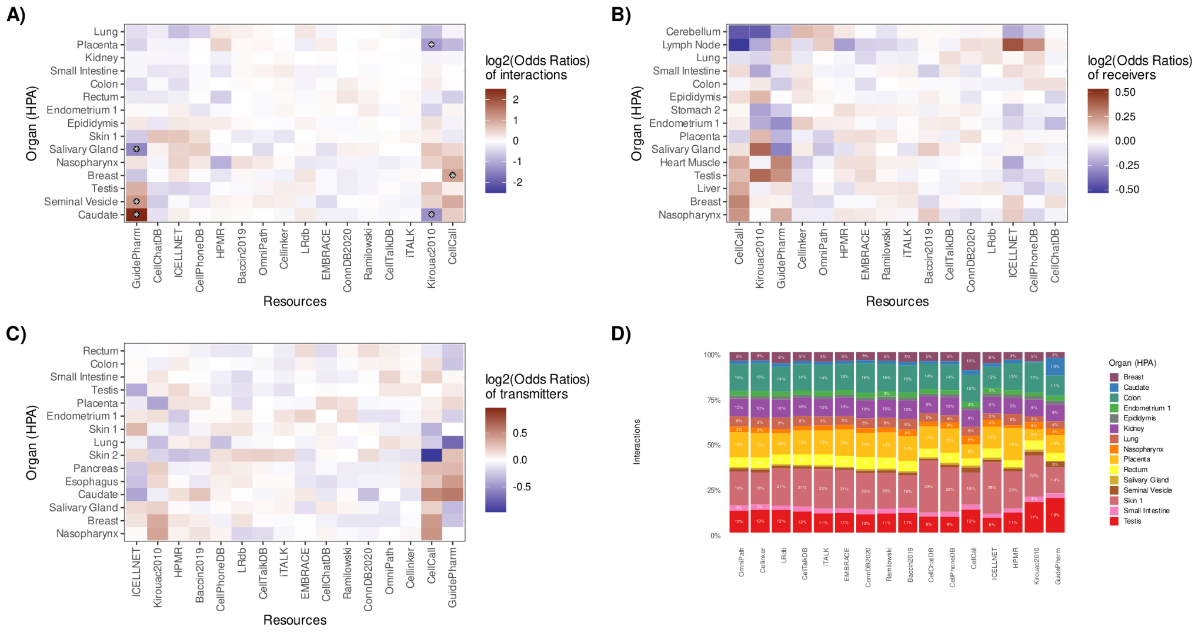

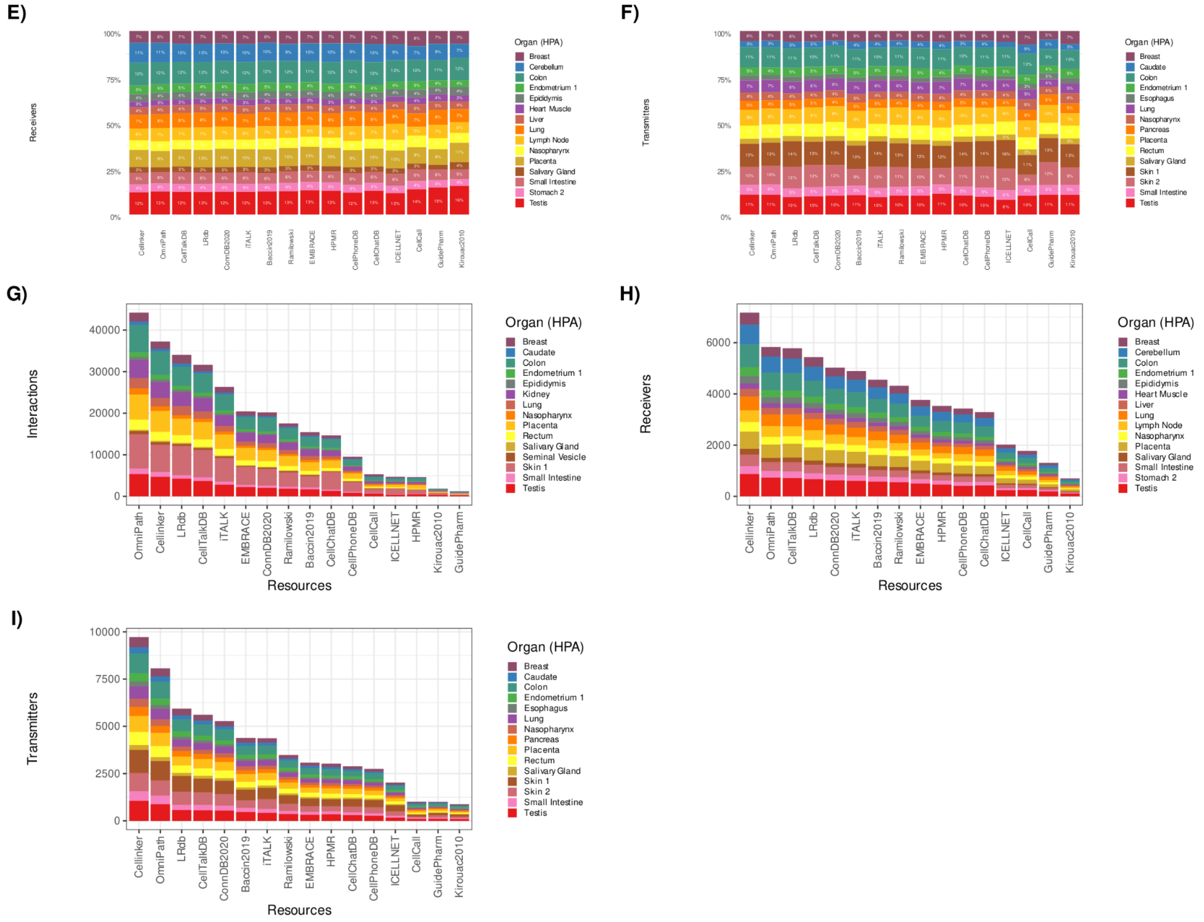
E)
Receivers
100%
75%
50%
25%
0%
Cellinker OmniPath CellTalkDB LRdb ConnDB2020 iTALK Baccin2019 Ramilowski EMBRACE HPMR CellPhoneDB CellChatDB ICELLNET CellCall GuidePharm Kirouac2010
Organ (HPA)
Breast
Cerebellum
Colon
Endometrium 1
Epididymis
Heart Muscle
Liver
Lung
Lymph Node
Nasopharynx
Placenta
Salivary Gland
Small Intestine
Stomach 2
Testis
F)
Transmitters
100%
75%
50%
25%
0%
Cellinker OmniPath LRdb CellTalkDB ConnDB2020 Baccin2019 iTALK Ramilowski EMBRACE HPMR CellChatDB CellPhoneDB ICELLNET CellCall GuidePharm Kirouac2010
Organ (HPA)
Breast
Caudate
Colon
Endometrium 1
Esophagus
Lung
Nasopharynx
Pancreas
Placenta
Rectum
Salivary Gland
Skin 1
Skin 2
Small Intestine
Testis
G)
Interactions
40000
30000
20000
10000
0
OmniPath Cellinker LRdb CellTalkDB iTALK EMBRACE ConnDB2020 Ramilowski Baccin2019 CellChatDB CellPhoneDB CellCall ICELLNET HPMR Kirouac2010 GuidePharm
Resources
Organ (HPA)
Breast
Caudate
Colon
Endometrium 1
Epididymis
Kidney
Lung
Nasopharynx
Placenta
Rectum
Salivary Gland
Seminal Vesicle
Skin 1
Small Intestine
Testis
H)
Receivers
6000
4000
2000
0
Cellinker OmniPath CellTalkDB LRdb ConnDB2020 iTALK Baccin2019 Ramilowski EMBRACE HPMR CellPhoneDB CellChatDB ICELLNET CellCall GuidePharm Kirouac2010
Resources
Organ (HPA)
Breast
Cerebellum
Colon
Endometrium 1
Epididymis
Heart Muscle
Liver
Lung
Lymph Node
Nasopharynx
Placenta
Salivary Gland
Small Intestine
Stomach 2
Testis
I)
Transmitters
10000
7500
5000
2500
0
Cellinker OmniPath LRdb CellTalkDB ConnDB2020 Baccin2019 iTALK Ramilowski EMBRACE HPMR CellChatDB CellPhoneDB ICELLNET CellCall GuidePharm Kirouac2010
Resources
Organ (HPA)
Breast
Caudate
Colon
Endometrium 1
Esophagus
Lung
Nasopharynx
Pancreas
Placenta
Rectum
Salivary Gland
Skin 1
Skin 2
Small Intestine
Testis

Supplementary Figure S8. *Interaction enrichment scores calculated for the largest 50 categories matched to organ-enriched proteins from the HPA database. Fisher's exact test was used to estimate the differentially-represented categories. Differentially represented (absolute(log2(Odds ratio)) > 1) categories were marked according to FDR-corrected p-values =< 0.05 (diamond; ◊), 0.01 (triangle; △), and 0.001 (8-pointed asterisk; ❋).*

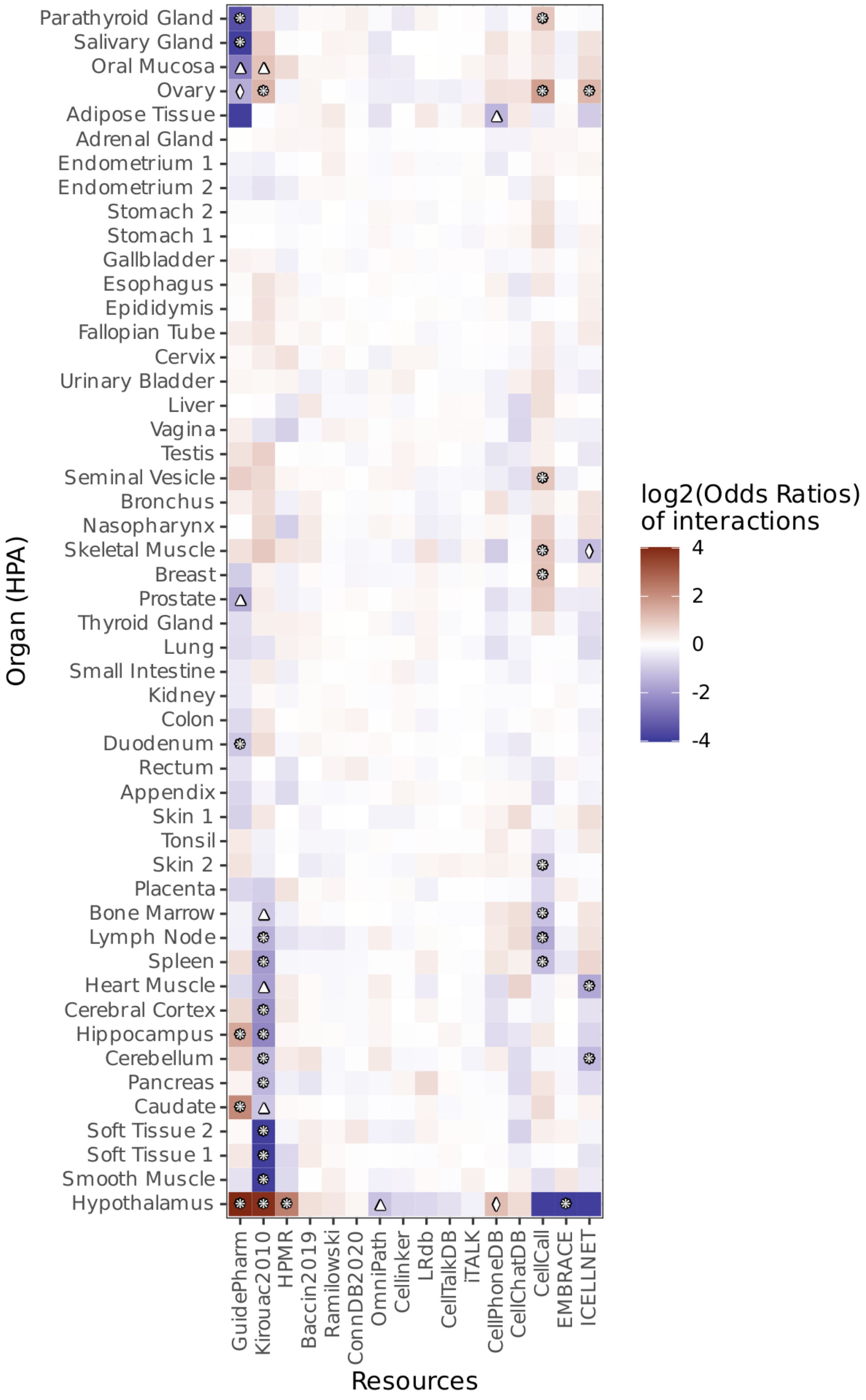

Supplementary Figure S9. *Tissue-enriched protein distributions across CCC resources. Enrichment Scores (A-C), Percentage Proportions (D-F), and Number of matches (G-I) to the 15 largest categories of tissue-enriched proteins in the HPA database per resource for Interactions, Receivers and Transmitters from different CCC resources. Fisher's exact test was used to estimate the differentially-represented categories. Differentially represented (absolute(log2(Odds ratio)) > 1) categories were marked according to FDR-corrected p-values =< 0.05 (diamond; ◊), 0.01 (triangle; △), and 0.001 (8-pointed asterisk; ✳).*

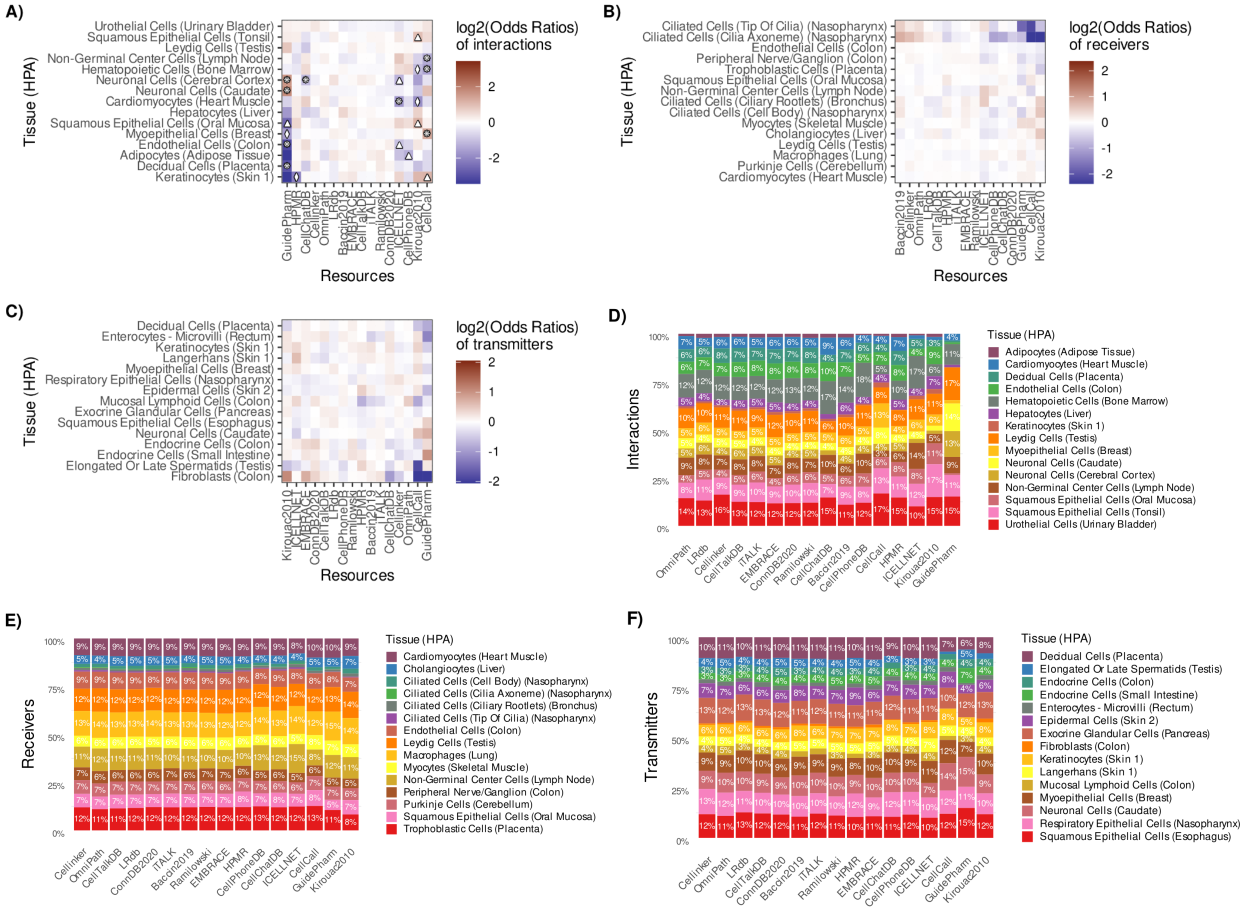

G)

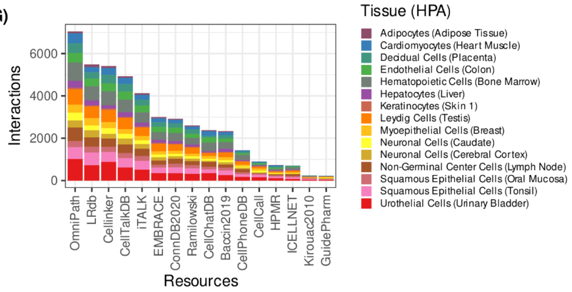

Interactions
0
2000
4000
6000
OmniPath
LRdb
Cellinker
CellTalkDB
iTALK
EMBRACE
ConnDB2020
Ramilowski
CellChatDB
Baccin2019
CellPhoneDB
CellCall
HPMR
ICELLNET
Kirouac2010
GuidePharm
Resources
Tissue (HPA)
Adipocytes (Adipose Tissue)
Cardiomyocytes (Heart Muscle)
Decidual Cells (Placenta)
Endothelial Cells (Colon)
Hematopoietic Cells (Bone Marrow)
Hepatocytes (Liver)
Keratinocytes (Skin 1)
Leydig Cells (Testis)
Myoepithelial Cells (Breast)
Neuronal Cells (Caudate)
Neuronal Cells (Cerebral Cortex)
Non-Germinal Center Cells (Lymph Node)
Squamous Epithelial Cells (Oral Mucosa)
Squamous Epithelial Cells (Tonsil)
Urothelial Cells (Urinary Bladder)

H)

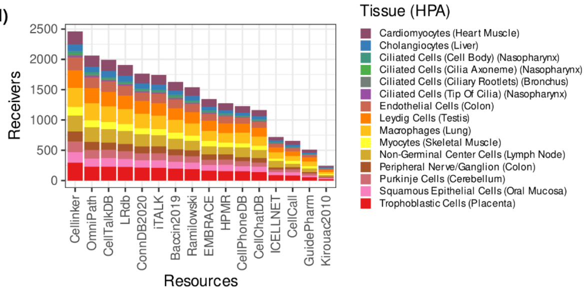

Receivers
0
500
1000
1500
2000
2500
Cellinker
OmniPath
CellTalkDB
LRdb
ConnDB2020
iTALK
Baccin2019
Ramilowski
EMBRACE
HPMR
CellPhoneDB
CellChatDB
ICELLNET
CellCall
GuidePharm
Kirouac2010
Resources
Tissue (HPA)
Cardiomyocytes (Heart Muscle)
Cholangiocytes (Liver)
Ciliated Cells (Cell Body) (Nasopharynx)
Ciliated Cells (Cilia Axoneme) (Nasopharynx)
Ciliated Cells (Ciliary Rootlets) (Bronchus)
Ciliated Cells (Tip Of Cilia) (Nasopharynx)
Endothelial Cells (Colon)
Leydig Cells (Testis)
Macrophages (Lung)
Myocytes (Skeletal Muscle)
Non-Germinal Center Cells (Lymph Node)
Peripheral Nerve/Ganglion (Colon)
Purkinje Cells (Cerebellum)
Squamous Epithelial Cells (Oral Mucosa)
Trophoblastic Cells (Placenta)

I)

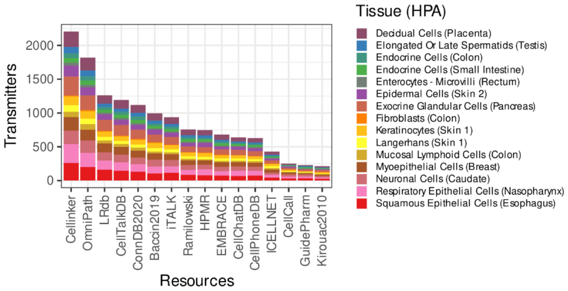

Transmitters
0
500
1000
1500
2000
Cellinker
OmniPath
LRdb
CellTalkDB
ConnDB2020
Baccin2019
iTALK
Ramilowski
HPMR
EMBRACE
CellChatDB
CellPhoneDB
ICELLNET
CellCall
GuidePharm
Kirouac2010
Resources
Tissue (HPA)
Decidual Cells (Placenta)
Elongated Or Late Spermatids (Testis)
Endocrine Cells (Colon)
Endocrine Cells (Small Intestine)
Enterocytes - Microvilli (Rectum)
Epidermal Cells (Skin 2)
Exocrine Glandular Cells (Pancreas)
Fibroblasts (Colon)
Keratinocytes (Skin 1)
Langerhans (Skin 1)
Mucosal Lymphoid Cells (Colon)
Myoepithelial Cells (Breast)
Neuronal Cells (Caudate)
Respiratory Epithelial Cells (Nasopharynx)
Squamous Epithelial Cells (Esophagus)

Supplementary Figure S10. *Interaction enrichment scores calculated for the largest 50 categories matched to tissue-enriched proteins from the HPA database. Fisher's exact test was used to estimate the differentially-represented categories. Differentially represented (absolute(log2(Odds ratio)) > 1) categories were marked according to FDR-corrected p-values =< 0.05 (diamond; ◊), 0.01 (triangle; △), and 0.001 (8-pointed asterisk; ✳).*

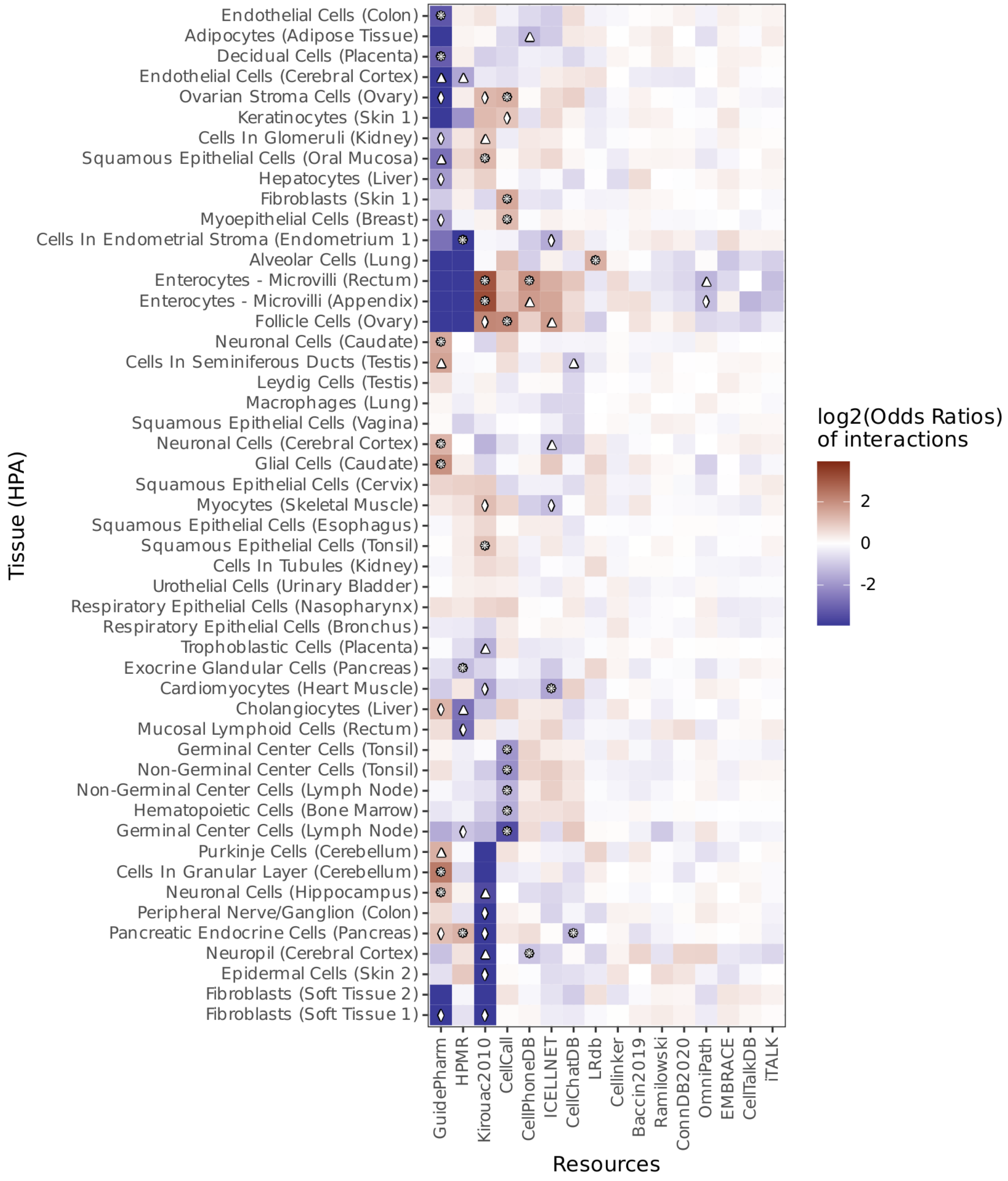

Supplementary Figure S11. *Disease-associated gene distributions across CCC resources. Enrichment Scores (A-C), Percentage Proportions (D-F), and Number of matches (G-I) to the DisGeNet database per resource for Interactions, Receivers and Transmitters from different CCC resources. Fisher's exact test was used to estimate the differentially-represented categories. No significant differentially-represented categories were detected following FDR correction.*

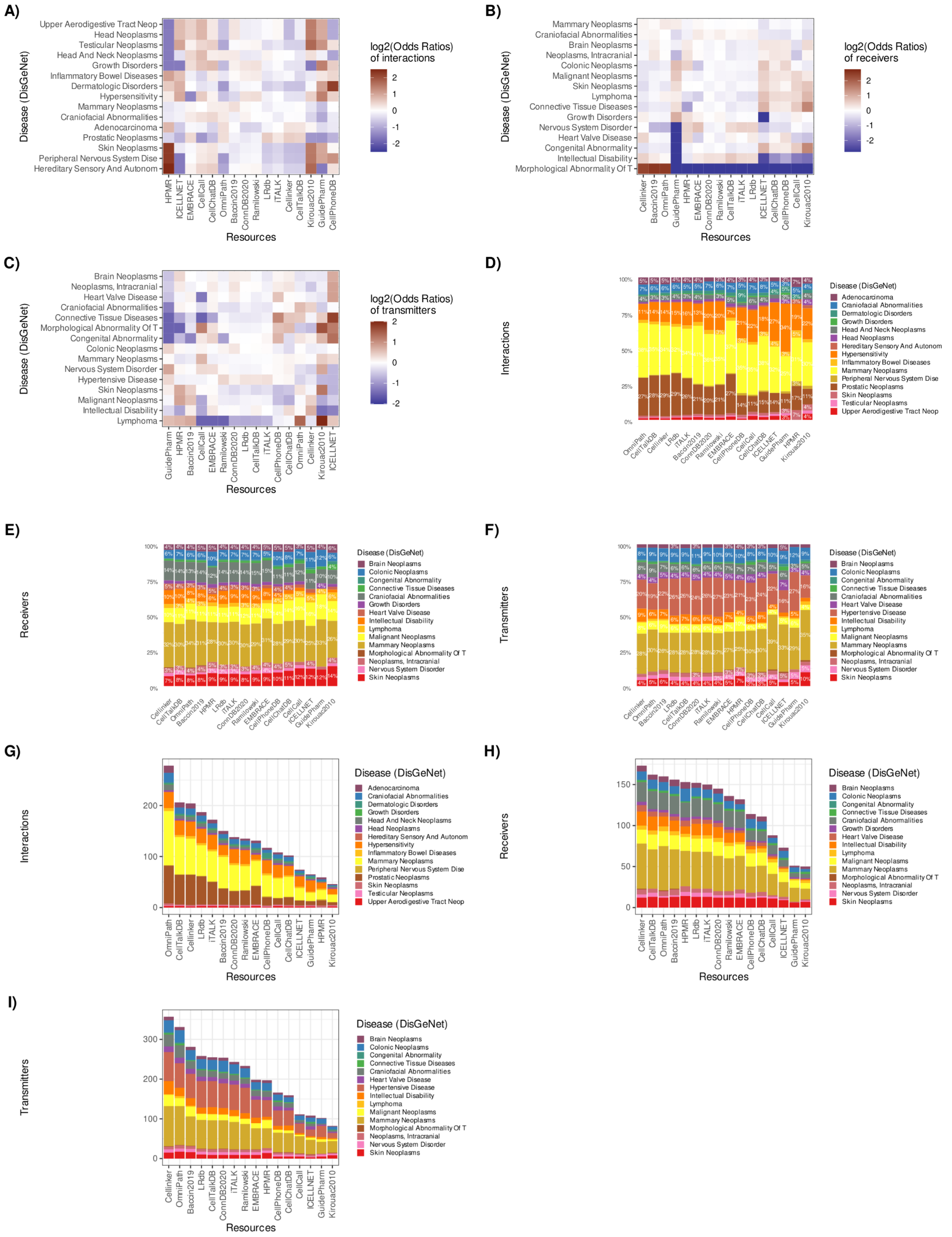

Supplementary Figure S12. *Overlap (Jaccard index) in the 1% highest ranked interactions A) when using the same Resource with different Methods (Blue; n=7) and B) when using the same Method with different Resources (Red; n=16). Boxplots represent the median pairwise jaccard index with hinges showing the first and third quartiles and whiskers extending 1.5 above and below the interquartile range.*

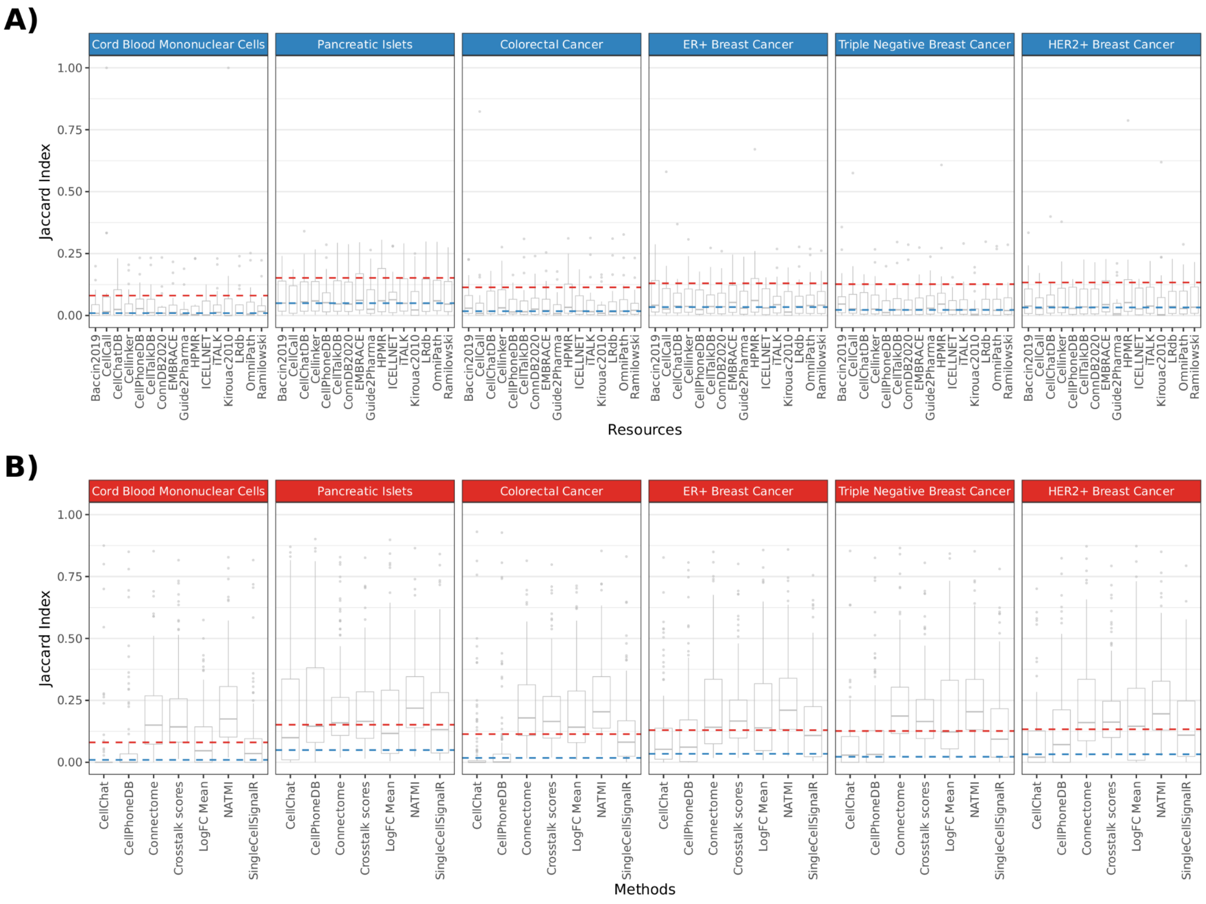

Supplementary Figure S13. *Robustness of cell-cell communication predictions to A) Cell type subsampling, B) Reshuffling of cell types labels C) Selective, and D) Non-selective replacement of interactions with putativelly false ones. The non-modified predictions (0% modifications) from each method were used to calculate the True Positive Rate. Boxplots represent the median true positive rate with hinges showing the first and third quartiles and whiskers extending 1.5 above and below the interquartile range (n = 5).*

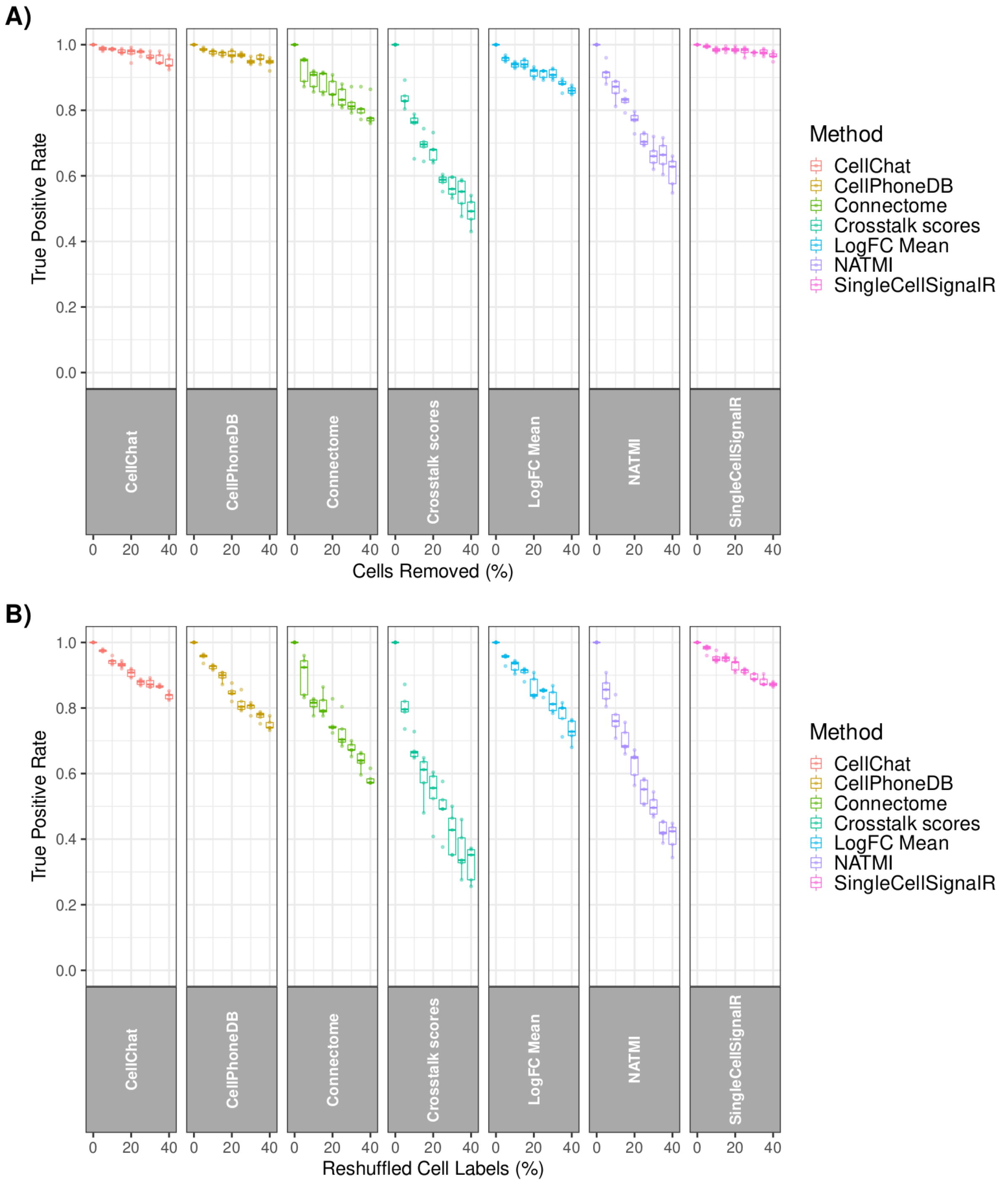

C)

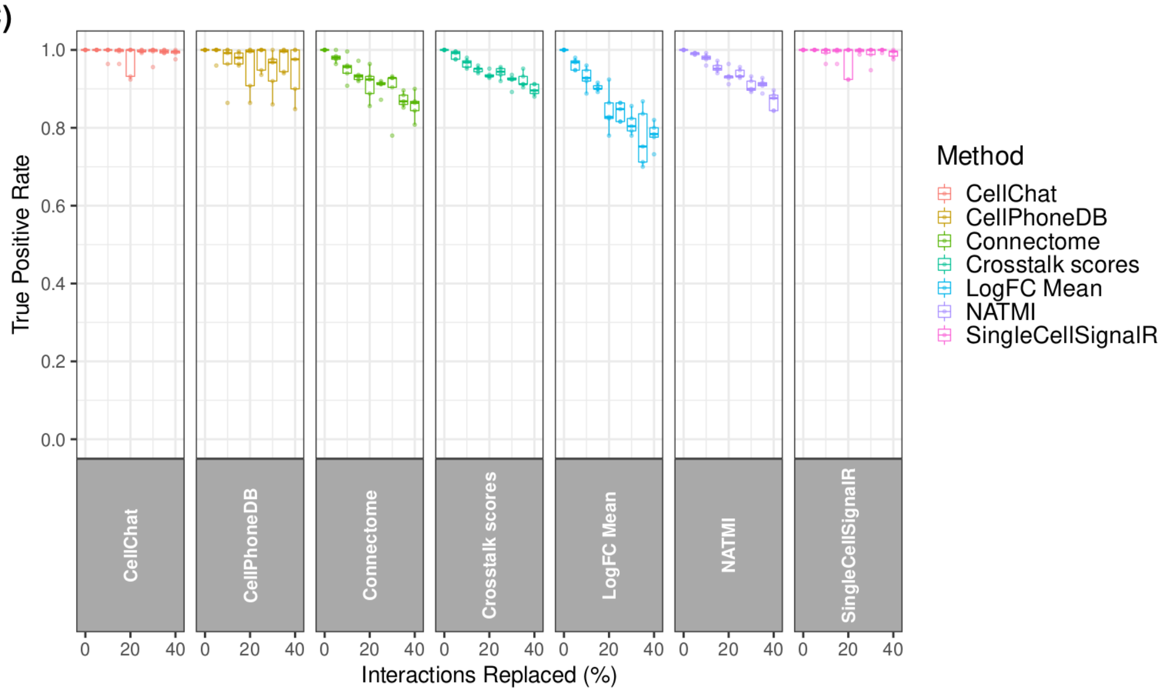
True Positive Rate
1.0
0.8
0.6
0.4
0.2
0.0
CellChat
CellPhoneDB
Connectome
Crosstalk scores
LogFC Mean
NATMI
SingleCellSignalR
0 20 40
Interactions Replaced (%)
Method
CellChat
CellPhoneDB
Connectome
Crosstalk scores
LogFC Mean
NATMI
SingleCellSignalR

D)

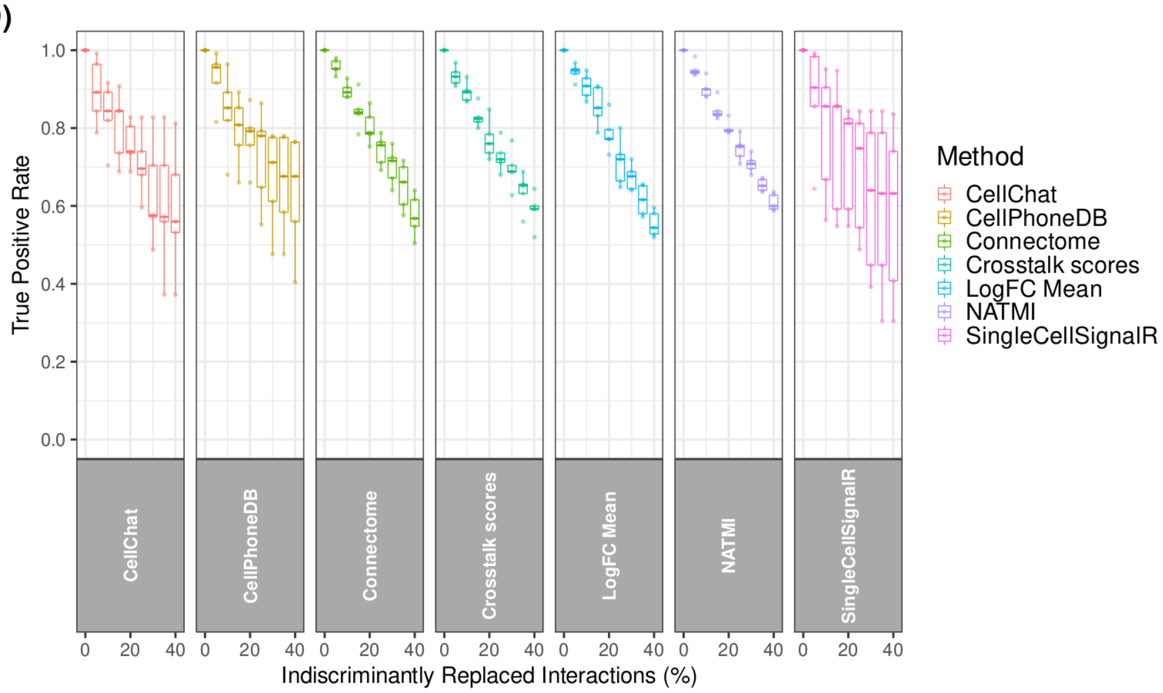
True Positive Rate
1.0
0.8
0.6
0.4
0.2
0.0
CellChat
CellPhoneDB
Connectome
Crosstalk scores
LogFC Mean
NATMI
SingleCellSignalR
0 20 40
Indiscriminantly Replaced Interactions (%)
Method
CellChat
CellPhoneDB
Connectome
Crosstalk scores
LogFC Mean
NATMI
SingleCellSignalR

Supplementary Figure S14. AUROC/AUPRC of CCC Method Agreement with Cytokine Activities. A) Each method's performance was estimated independently, i.e. only using the interactions predicted following the preprocessing steps of each method. B) Methods were evaluated using the union of all interactions predictions, and missing interactions were max-imputed. To ameliorate the differences of predictions assigned to the negative class between the different methods, we included a subsampling step, in which we downsampled the negative class 100 times to match the (lower) number of interactions assigned to the positive class.

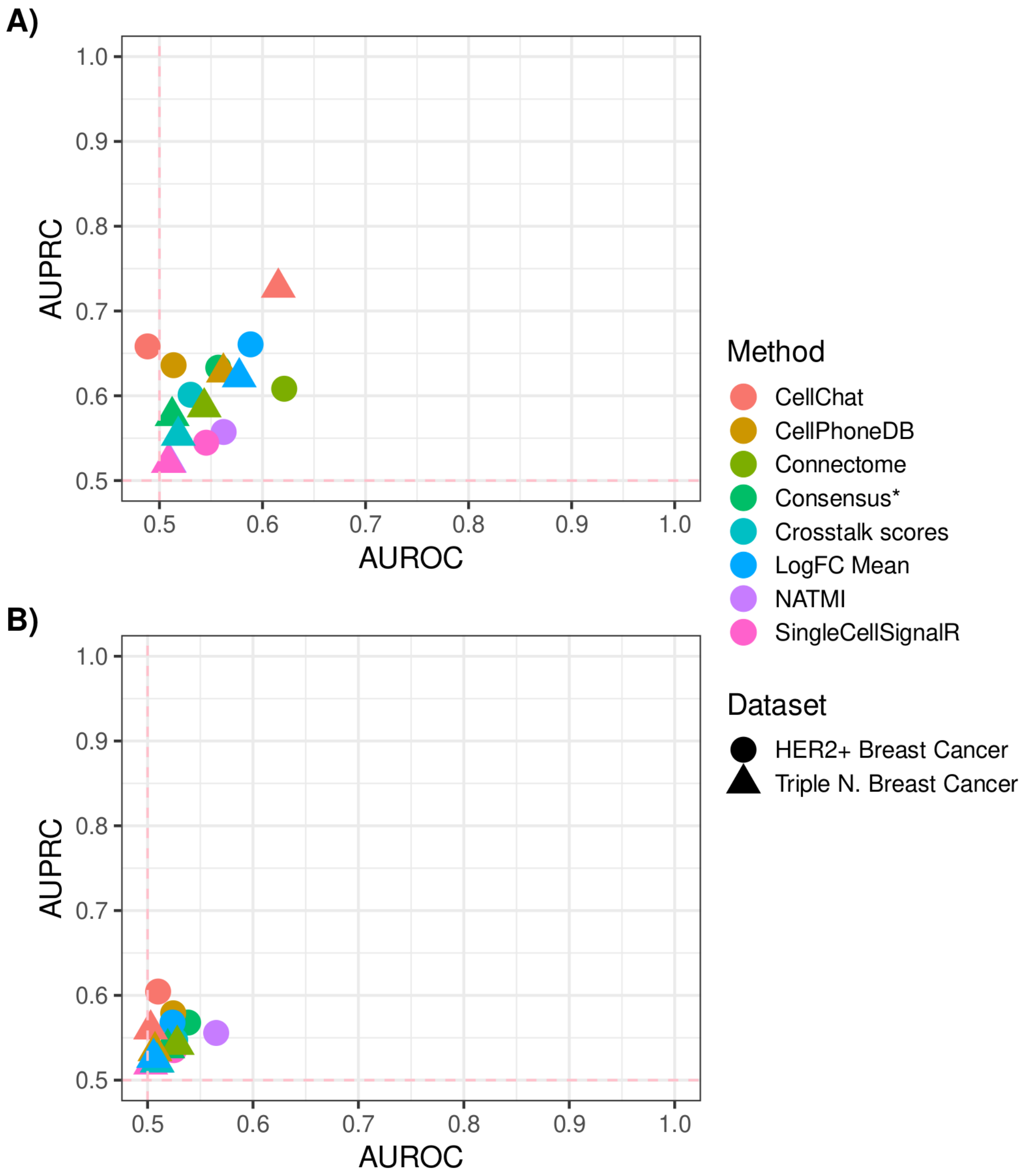

Supplementary Figure S15. Odds ratios of colocalized cell types among the highest ranked interaction predictions, across a ranked range between 50 and 5,000 in mer- and seqFISH datasets. Colocalized cell types were identified using the neighbourhood enrichment analysis, implemented in Squidpy. Odds ratios were calculated using Fisher's exact test. We use a more constrained rank range than the Visium colocalisation, due to the relatively lower gene coverage of the fluorescence-based spatial technologies. Consensus represents the aggregated ranks of all interactions predicted by the methods.

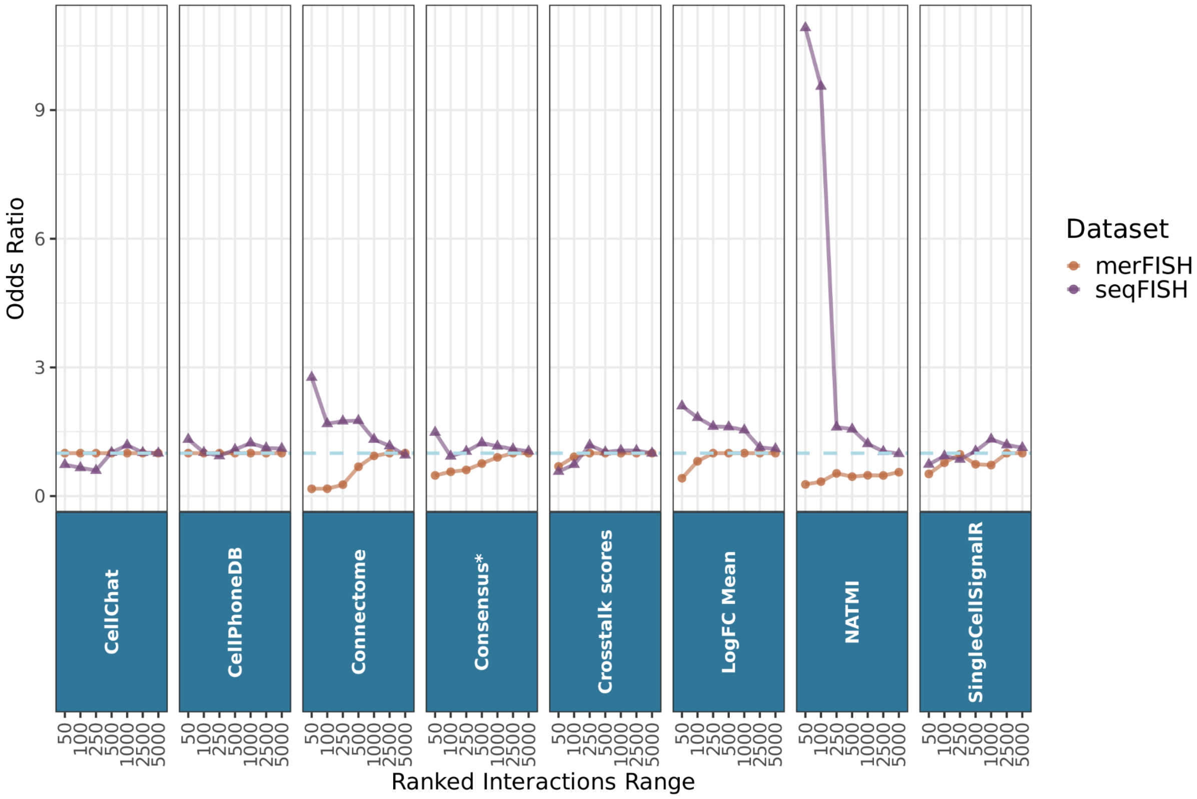

Supplementary Figure S16. Heatmap of median Jaccard index between the highest ranked 1,000 interactions per resource-method combination across <u>all</u> datasets. Clustered by Euclidean distance.

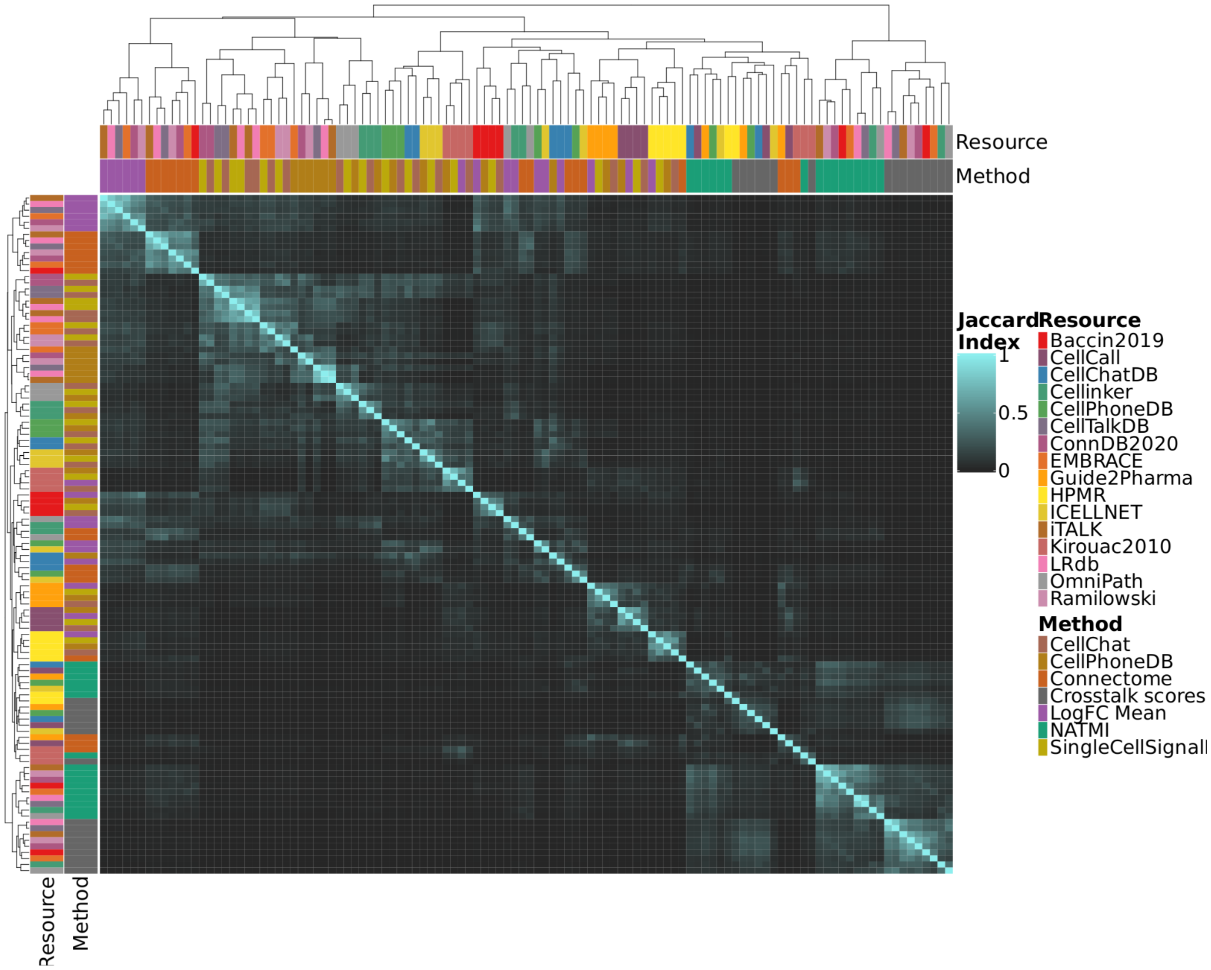

Supplementary Figure S17. Heatmap of median Jaccard index between the 1% highest ranked of interactions per resource-method combination across all datasets. Clustered by Euclidean distance.

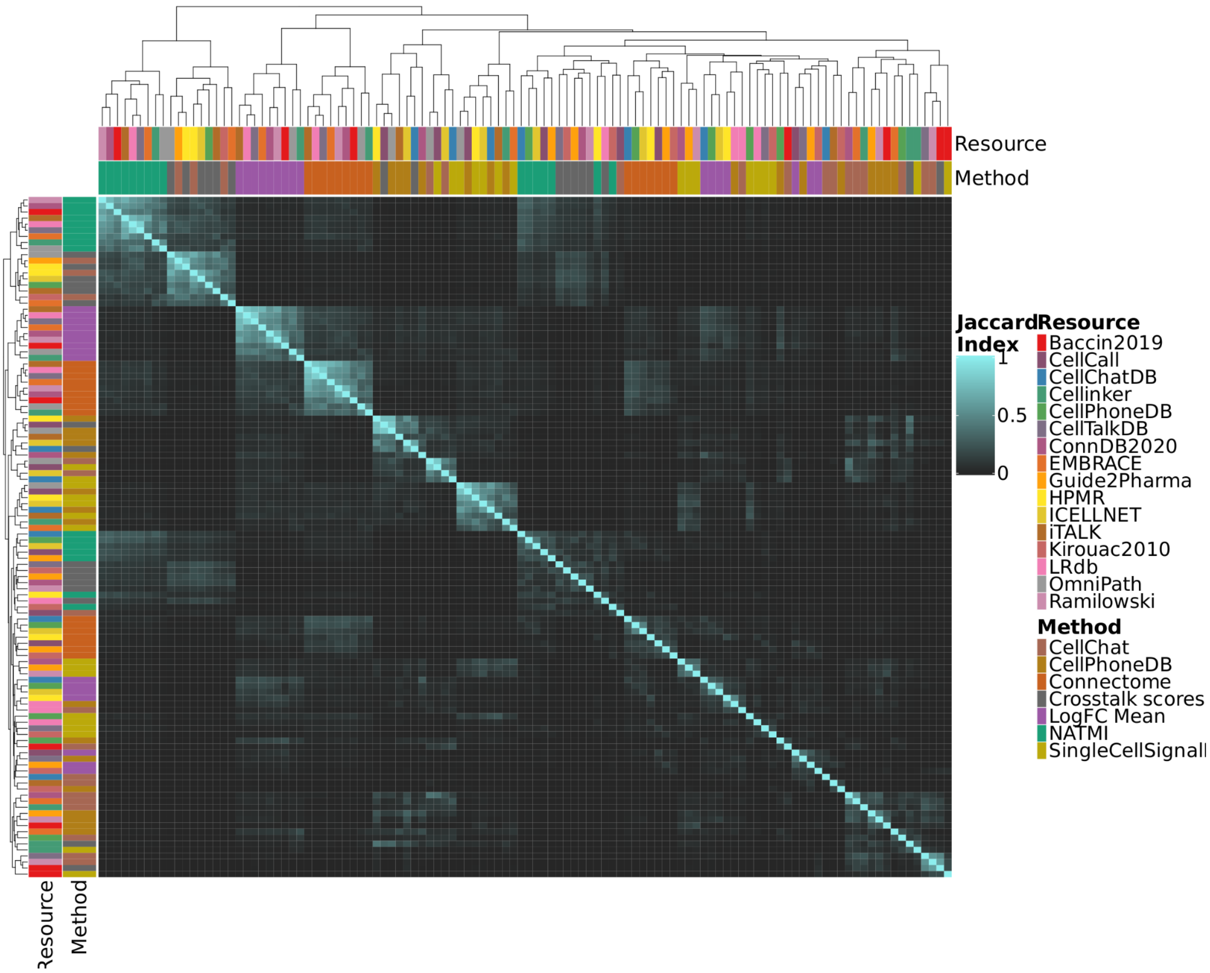

Supplementary Figure S18. *Overlap (Jaccard index) in the 1,000 highest ranked A) when using the same Resource with different Methods (Blue; n=7) and B) when using the same Method with different Resources (Red; n=16). Boxplots represent the median pairwise jaccard index with hinges showing the first and third quartiles and whiskers extending 1.5 above and below the interquartile range; the lines overlap for the CMBCs dataset. Here, we use only the p-values for CellPhoneDB and CellChat, not their 'composite' scores (* ***Supp. table 3****).*

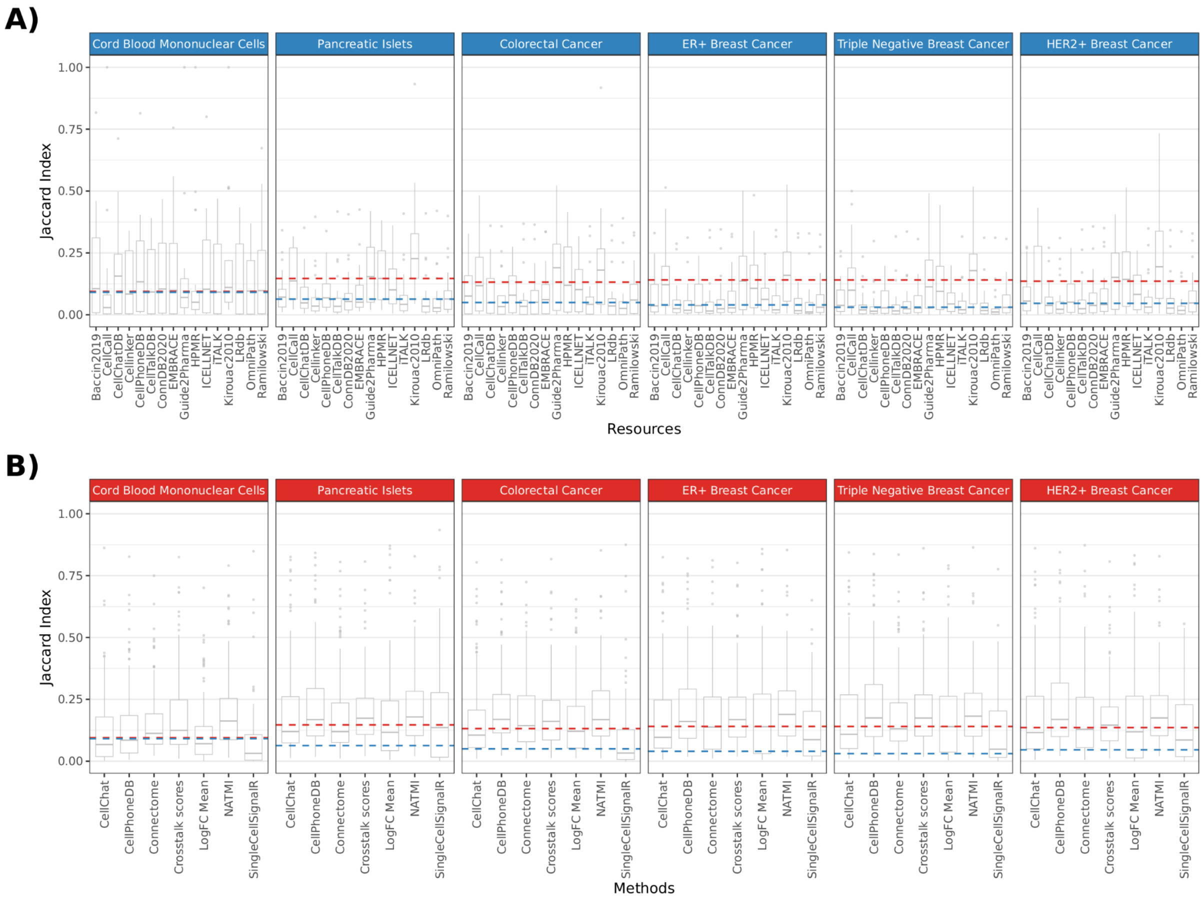

Supplementary Figure S19. *Heatmap of median Jaccard index between the highest ranked 1,000 interactions per resource-method combination across all datasets when using only the p-values for CellPhoneDB and CellChat. Clustered by Euclidean distance (* ***Supp. table 3****).*

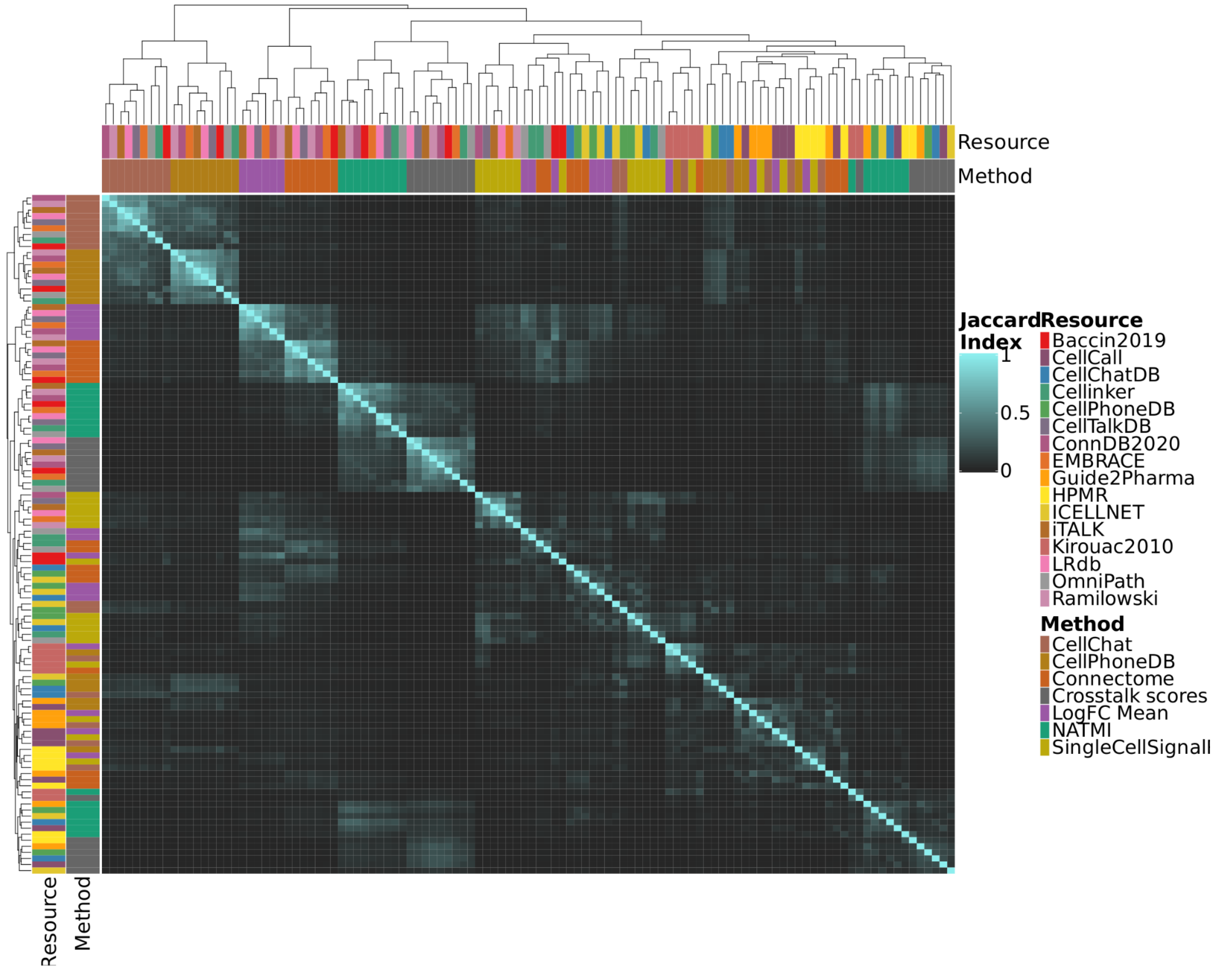

Supplementary Figure S20. *Overlap (Jaccard index) in the 1,000 highest ranked, non-specific interactions A) when using the same Resource with different Methods (Blue; n=7) and B) when using the same Method with different Resources (Red; n=16). Boxplots represent the median pairwise jaccard index with hinges showing the first and third quartiles and whiskers extending 1.5 above and below the interquartile range. Here, we use non-specific scoring functions for each method, if available (**Supp. table 3**).*

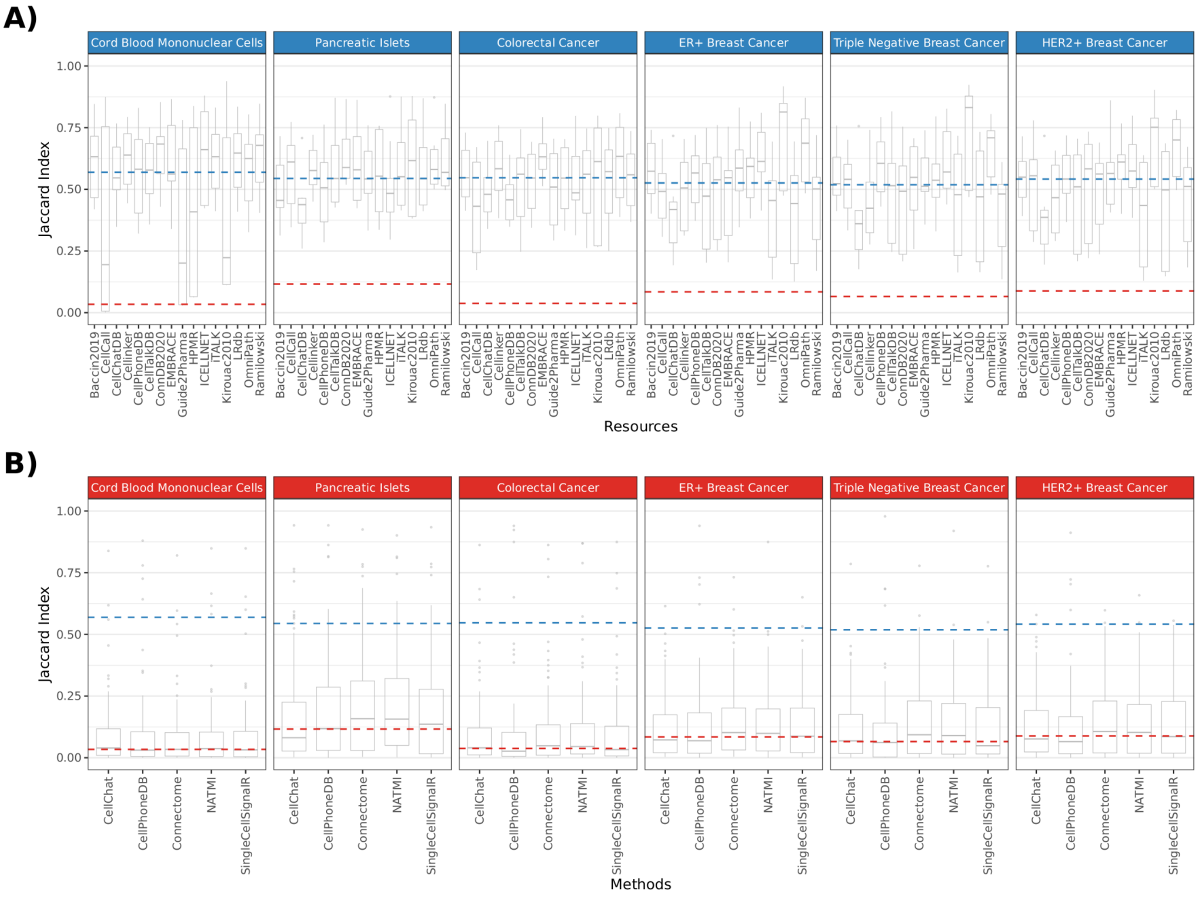

Supplementary Figure S21. *Heatmap of median Jaccard index between the 1,000 highest ranked, non-specific interactions per resource-method combination across all datasets. Clustered by Euclidean distance.*

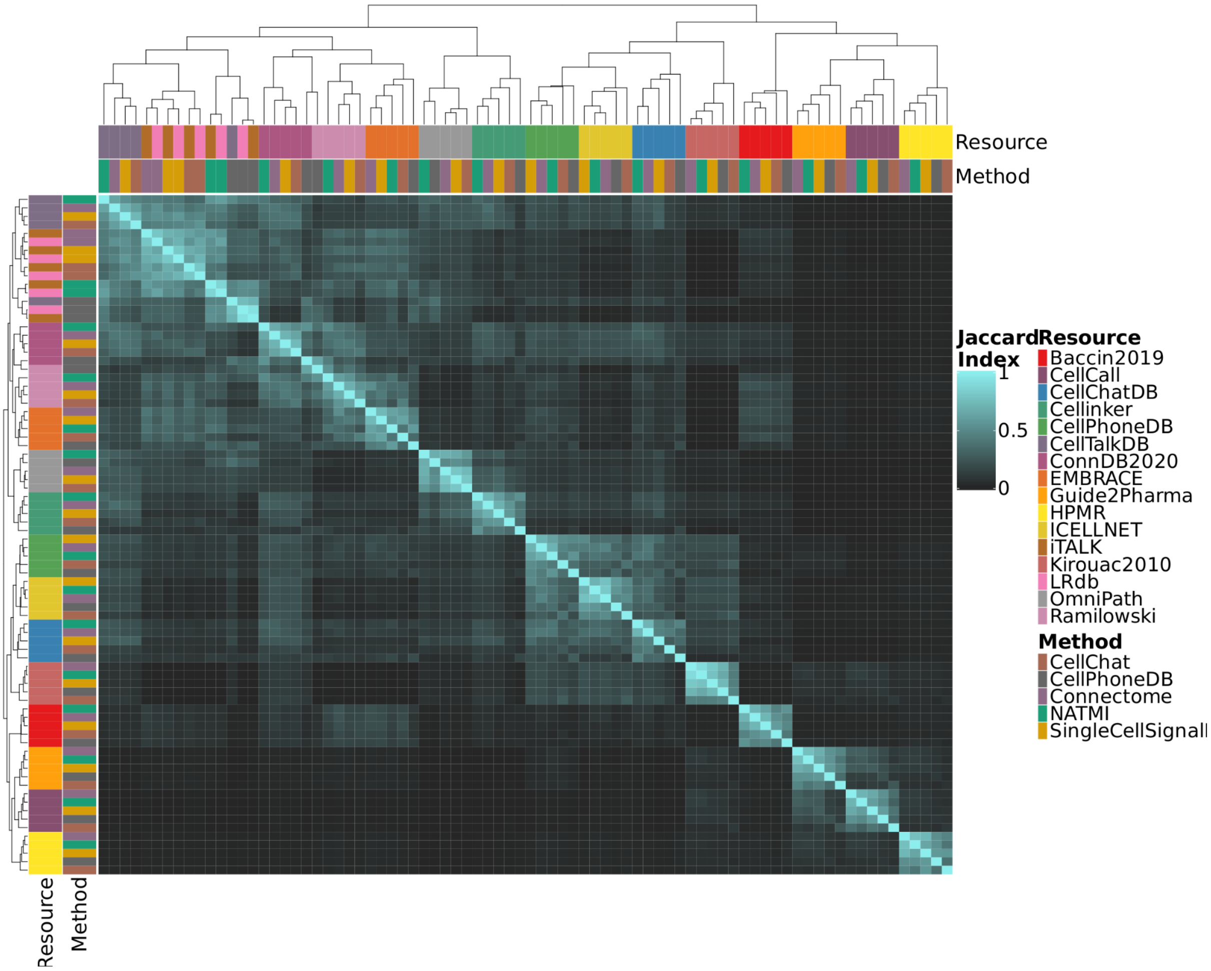

Supplementary Figure S22. *Relative frequencies of interactions per cell type estimated using the 1,000 highest ranked interactions for* ***A)*** *CBMCs,* ***B)*** *Colorectal cancer,* ***C)*** *ER+ breast cancer,* ***D)*** *HER2+ breast cancer,* ***E)*** *Triple negative breast cancer, and* ***F)*** *Pancreatic Islet datasets. The relative frequencies were defined as the number of interactions assigned to each cell type as 'source' or 'target' in the 1,000 highest ranked interactions.*

A)

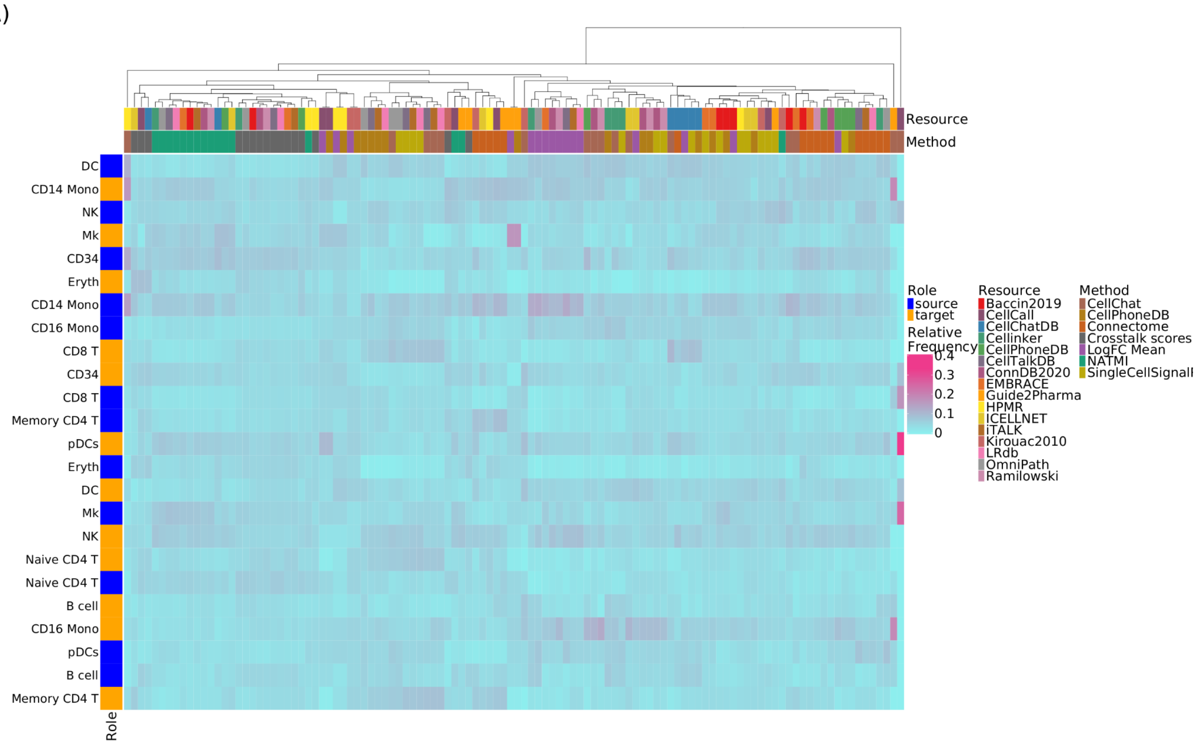

B)

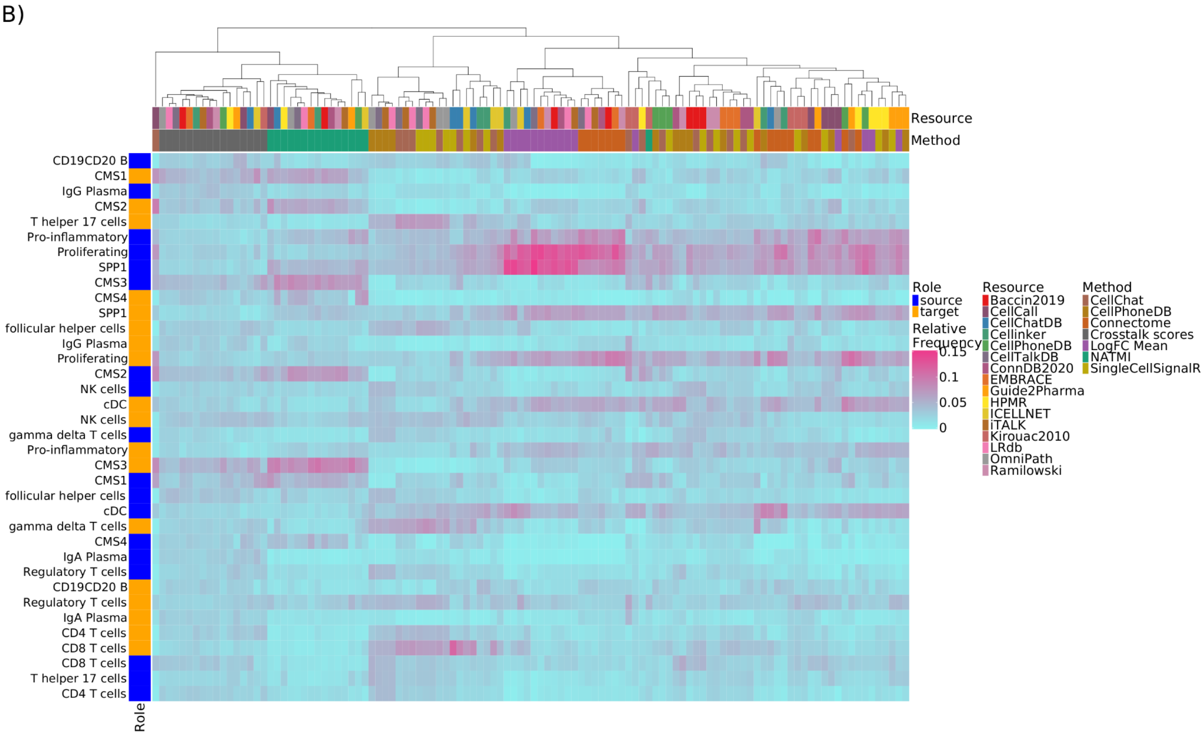

C)

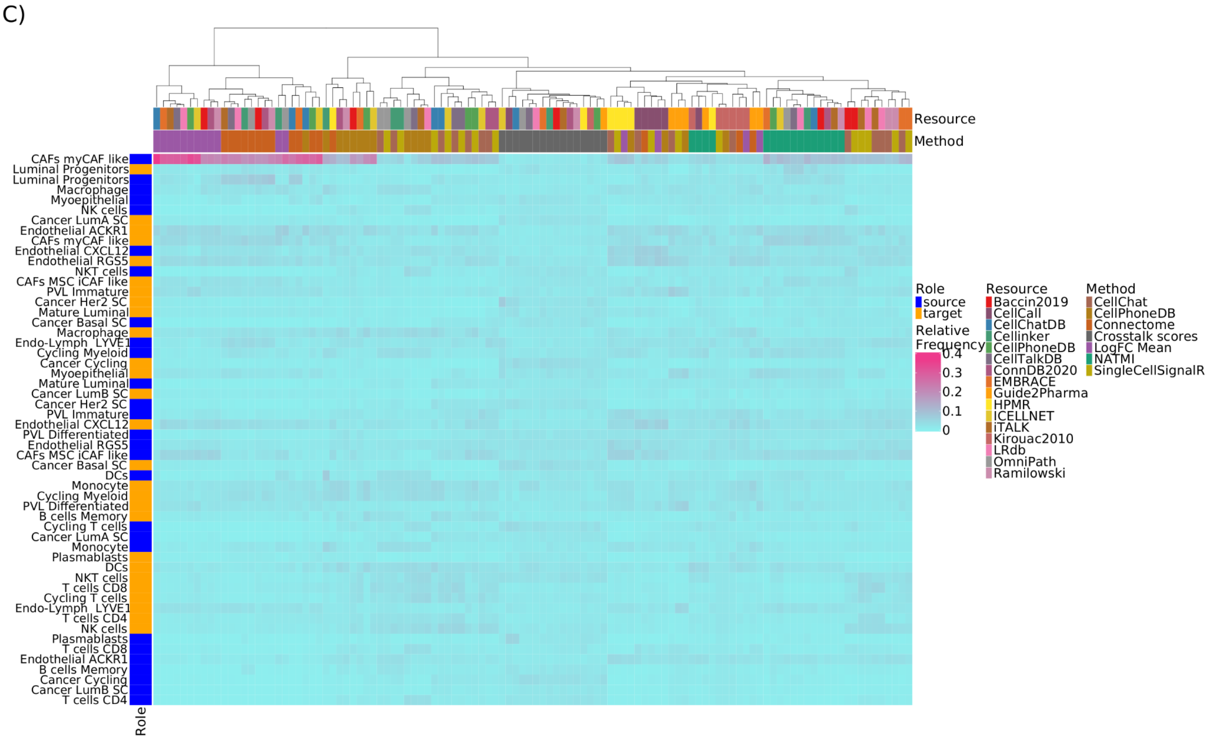
Resource
Method
CAFs myCAF like
Luminal Progenitors
Luminal Progenitors
Macrophage
Myoepithelial
NK cells
Cancer LumA SC
Endothelial ACKR1
CAFs myCAF like
Endothelial CXCL12
Endothelial RGS5
NKT cells
CAFs MSC iCAF like
PVL Immature
Cancer Her2 SC
Mature Luminal
Cancer Basal SC
Macrophage
Endo-Lymph_LYVE1
Cycling Myeloid
Cancer Cycling
Myoepithelial
Mature Luminal
Cancer LumB SC
Cancer Her2 SC
PVL Immature
Endothelial CXCL12
PVL Differentiated
Endothelial RGS5
CAFs MSC iCAF like
Cancer Basal SC
DCs
Monocyte
Cycling Myeloid
PVL Differentiated
B cells Memory
Cycling T cells
Cancer LumA SC
Monocyte
Plasmablasts
DCs
NKT cells
T cells CD8
Cycling T cells
Endo-Lymph_LYVE1
T cells CD4
NK cells
Plasmablasts
T cells CD8
Endothelial ACKR1
B cells Memory
Cancer Cycling
Cancer LumB SC
T cells CD4
Role
Role
source
target
Relative Frequency
0.4
0.3
0.2
0.1
0
Resource
Baccin2019
CellCall
CellChatDB
Cellinker
CellPhoneDB
CellTalkDB
ConnDB2020
EMBRACE
Guide2Pharma
HPMR
ICELLNET
iTALK
Kirouac2010
LRdb
OmniPath
Ramilowski
Method
CellChat
CellPhoneDB
Connectome
Crosstalk scores
LogFC Mean
NATMI
SingleCellSignalR

D)

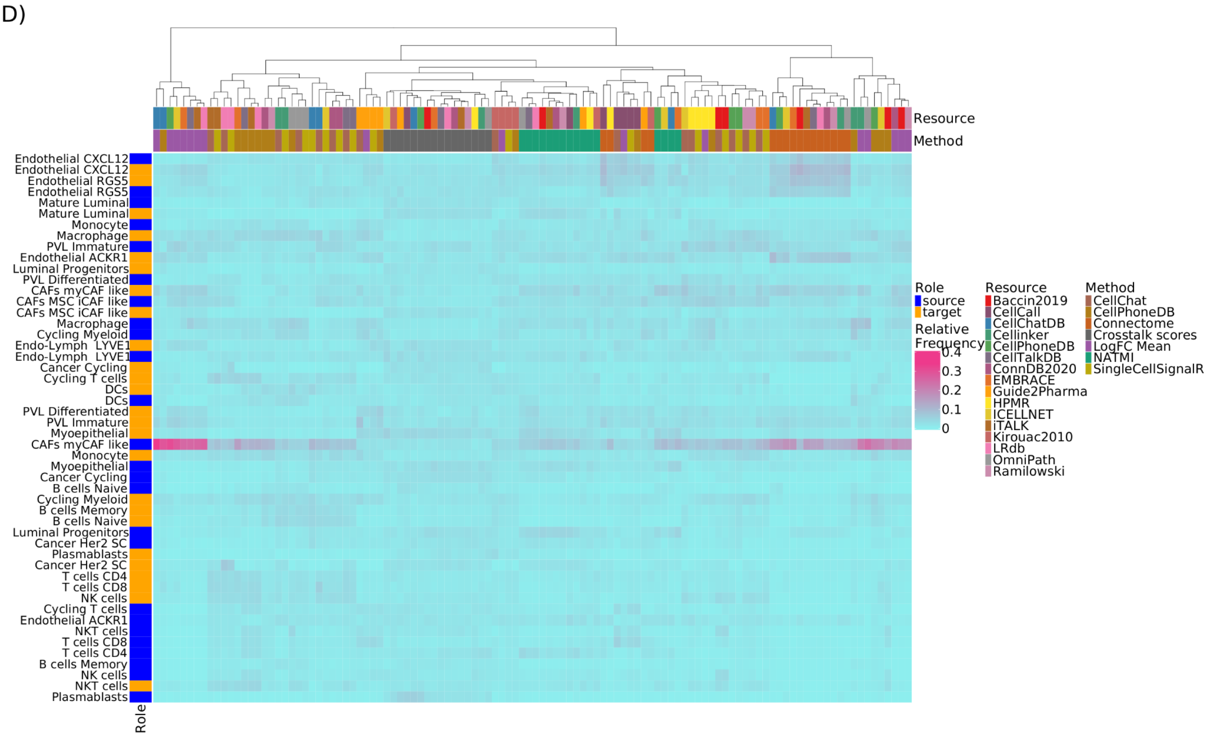
Resource
Method
Endothelial CXCL12
Endothelial CXCL12
Endothelial RGS5
Endothelial RGS5
Mature Luminal
Mature Luminal
Monocyte
Macrophage
PVL Immature
Endothelial ACKR1
Luminal Progenitors
PVL Differentiated
CAFs myCAF like
CAFs MSC iCAF like
CAFs MSC iCAF like
Macrophage
Cycling Myeloid
Endo-Lymph_LYVE1
Endo-Lymph_LYVE1
Cancer Cycling
Cycling T cells
DCs
DCs
PVL Differentiated
PVL Immature
Myoepithelial
CAFs myCAF like
Monocyte
Myoepithelial
Cancer Cycling
B cells Naive
Cycling Myeloid
B cells Memory
B cells Naive
Luminal Progenitors
Cancer Her2 SC
Plasmablasts
Cancer Her2 SC
T cells CD4
T cells CD8
NK cells
Cycling T cells
Endothelial ACKR1
NKT cells
T cells CD8
T cells CD4
B cells Memory
NK cells
NKT cells
Plasmablasts
Role
Role
source
target
Relative Frequency
0.4
0.3
0.2
0.1
0
Resource
Baccin2019
CellCall
CellChatDB
Cellinker
CellPhoneDB
CellTalkDB
ConnDB2020
EMBRACE
Guide2Pharma
HPMR
ICELLNET
iTALK
Kirouac2010
LRdb
OmniPath
Ramilowski
Method
CellChat
CellPhoneDB
Connectome
Crosstalk scores
LogFC Mean
NATMI
SingleCellSignalR

E)

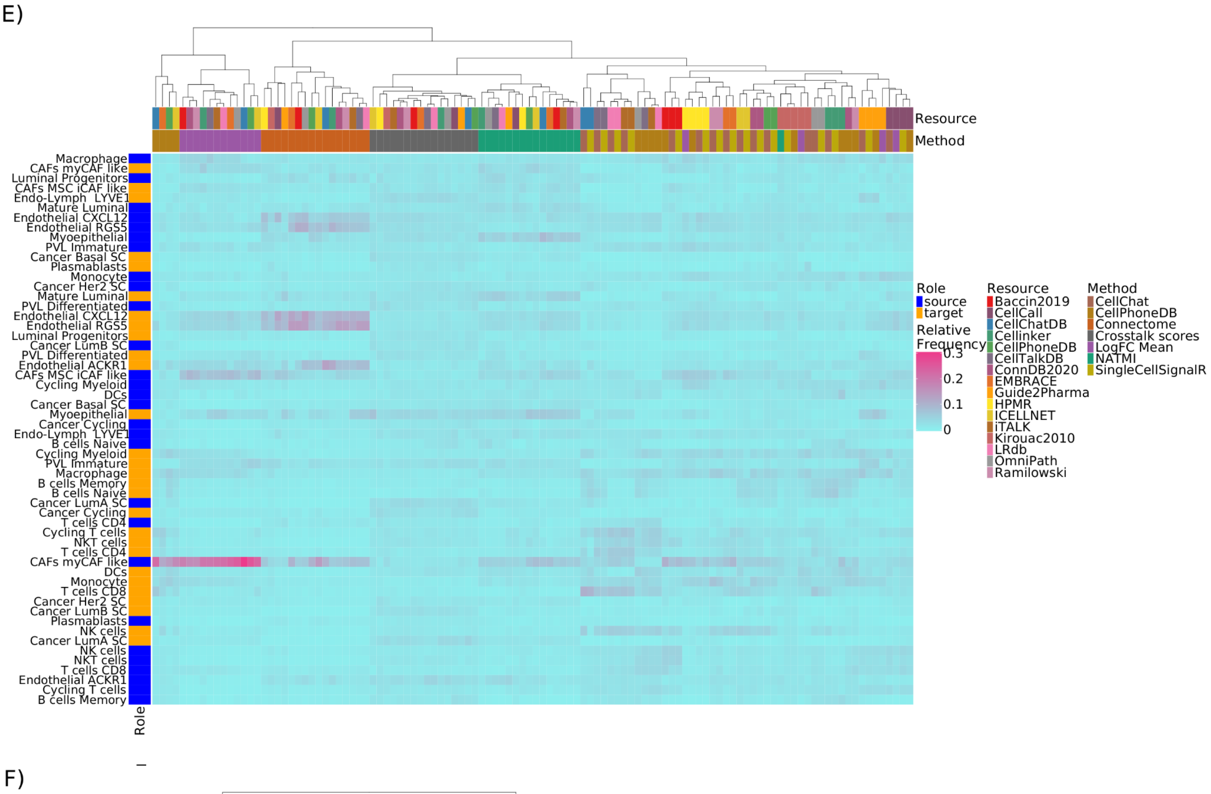
Resource
Method
Macrophage
CAFs myCAF like
Luminal Progenitors
CAFs MSC iCAF like
Endo-Lymph LYVE1
Mature Luminal
Endothelial CXCL12
Endothelial RGS5
Myoepithelial
PVL Immature
Cancer Basal SC
Plasmablasts
Monocyte
Cancer Her2 SC
Mature Luminal
PVL Differentiated
Endothelial CXCL12
Endothelial RGS5
Luminal Progenitors
Cancer LumB SC
PVL Differentiated
Endothelial ACKR1
CAFs MSC iCAF like
Cycling Myeloid
DCs
Cancer Basal SC
Myoepithelial
Cancer Cycling
Endo-Lymph LYVE1
B cells Naive
Cycling Myeloid
PVL Immature
Macrophage
B cells Memory
B cells Naive
Cancer LumA SC
Cancer Cycling
T cells CD4
Cycling T cells
NKT cells
T cells CD4
CAFs myCAF like
DCs
Monocyte
T cells CD8
Cancer Her2 SC
Cancer LumB SC
Plasmablasts
NK cells
Cancer LumA SC
NK cells
NKT cells
T cells CD8
Endothelial ACKR1
Cycling T cells
B cells Memory
Role
Role
source
target
Relative Frequency
0.3
0.2
0.1
0
Resource
Baccin2019
CellCall
CellChatDB
Cellinker
CellPhoneDB
CellTalkDB
ConnDB2020
EMBRACE
Guide2Pharma
HPMR
ICELLNET
iTALK
Kirouac2010
LRdb
OmniPath
Ramilowski
Method
CellChat
CellPhoneDB
Connectome
Crosstalk scores
LogFC Mean
NATMI
SingleCellSignalR

F)

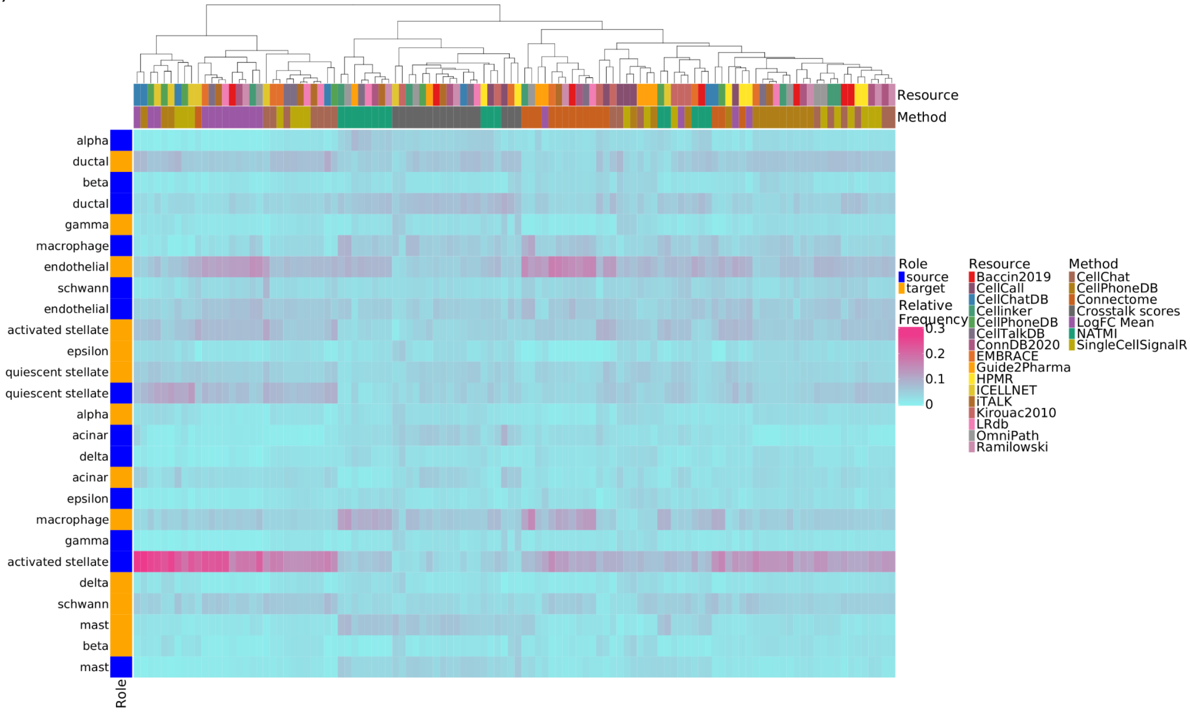
Resource
Method
alpha
ductal
beta
ductal
gamma
macrophage
endothelial
schwann
endothelial
activated stellate
epsilon
quiescent stellate
quiescent stellate
alpha
acinar
delta
acinar
epsilon
macrophage
gamma
activated stellate
delta
schwann
mast
beta
mast
Role
Role
source
target
Relative Frequency
0.3
0.2
0.1
0
Resource
Baccin2019
CellCall
CellChatDB
Cellinker
CellPhoneDB
CellTalkDB
ConnDB2020
EMBRACE
Guide2Pharma
HPMR
ICELLNET
iTALK
Kirouac2010
LRdb
OmniPath
Ramilowski
Method
CellChat
CellPhoneDB
Connectome
Crosstalk scores
LogFC Mean
NATMI
SingleCellSignalR

Supplementary Figure S23. *Relative frequencies of interactions per cell type estimated using the 1,000 highest ranked, non-specific interactions for **A)** CBMCs, **B)** Colorectal cancer, **C)** ER+ breast cancer, **D)** HER2+ breast cancer, **E)** Triple negative breast cancer, and **F)** Pancreatic Islet datasets. The relative frequencies were defined as the number of interactions assigned to each cell type as 'source' or 'target' in the 1,000 highest ranked, non-specific interactions.*

A)

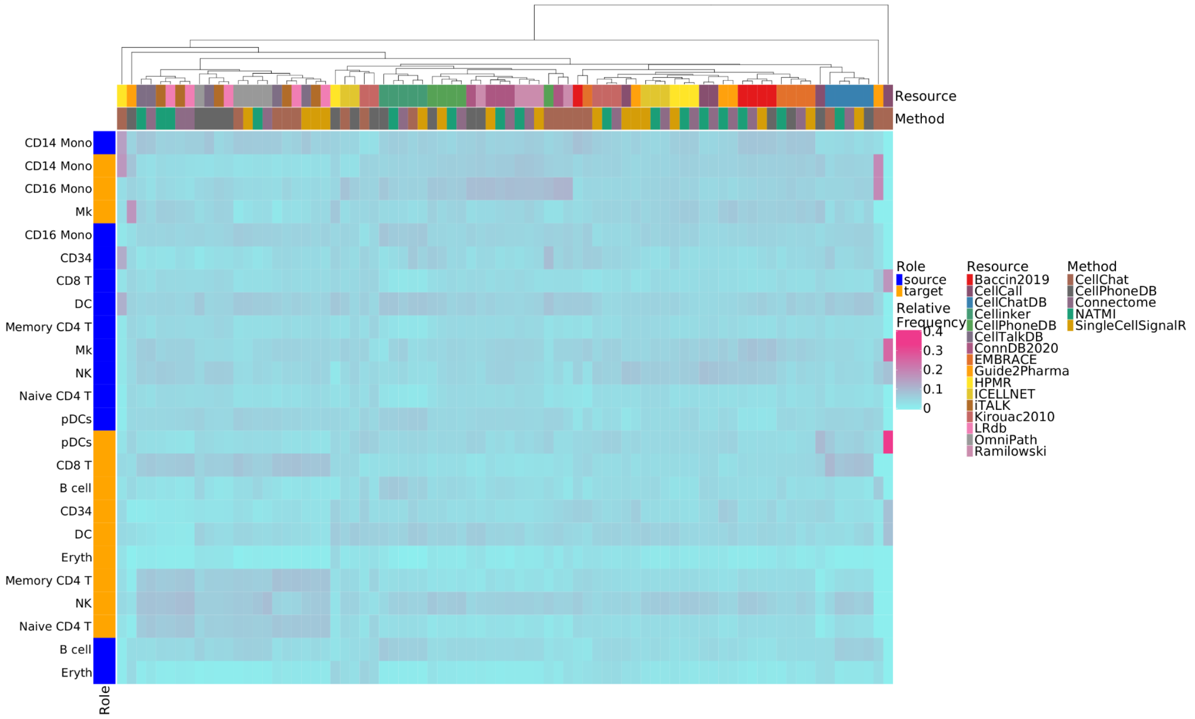

B)

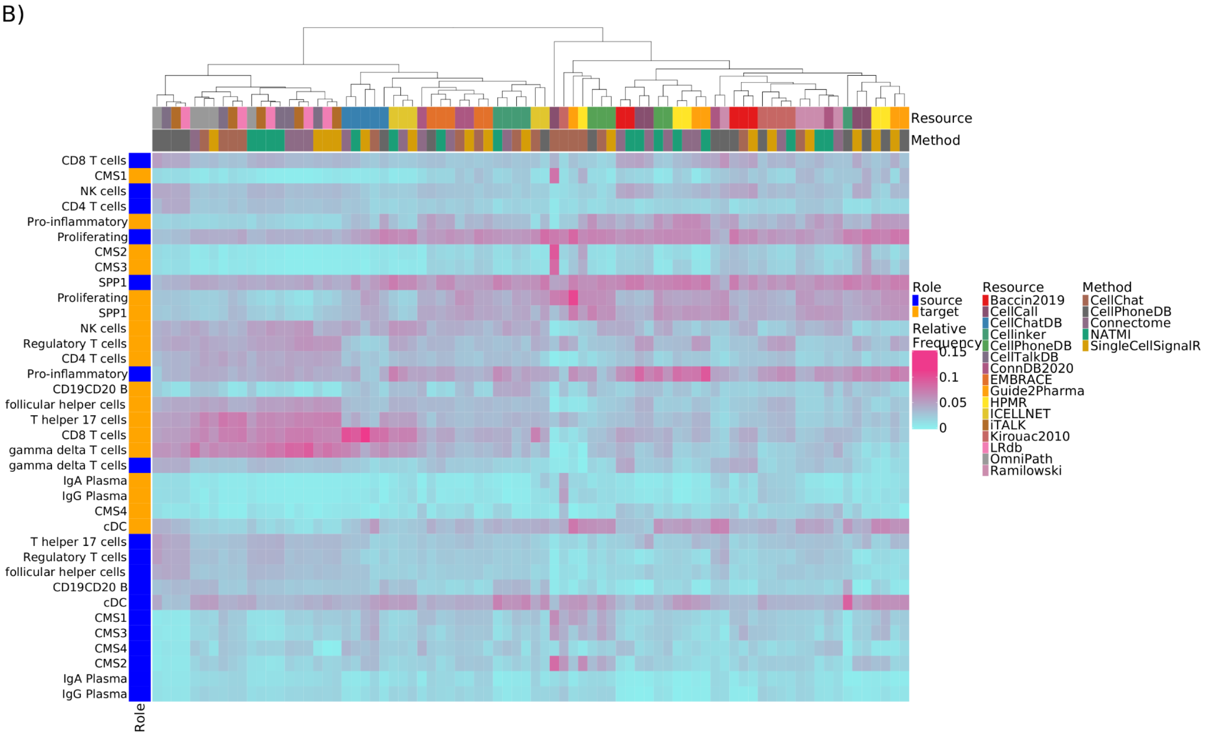

C)

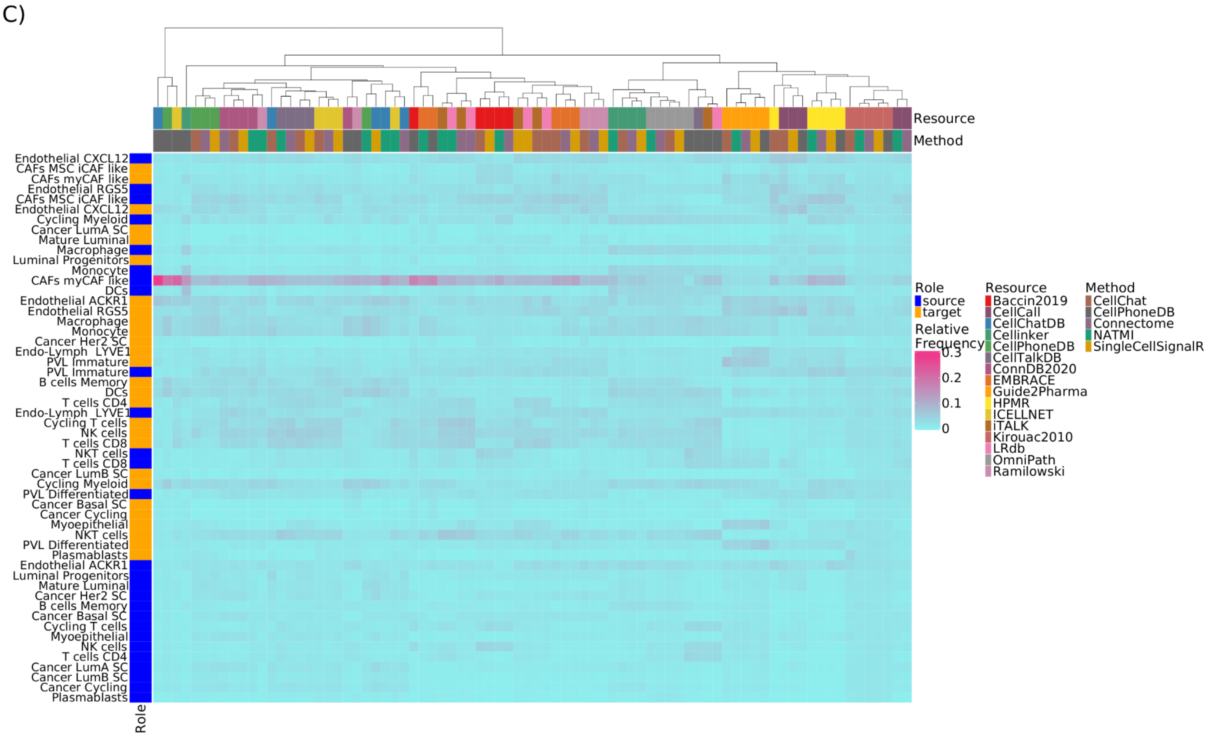
Resource
Method
Endothelial CXCL12
CAFs MSC iCAF like
CAFs myCAF like
Endothelial RGS5
CAFs MSC iCAF like
Endothelial CXCL12
Cycling Myeloid
Cancer LumA SC
Mature Luminal
Macrophage
Luminal Progenitors
Monocyte
CAFs myCAF like
DCs
Endothelial ACKR1
Endothelial RGS5
Macrophage
Monocyte
Cancer Her2 SC
Endo-Lymph_LYVE1
PVL Immature
PVL Immature
B cells Memory
DCs
T cells CD4
Endo-Lymph_LYVE1
Cycling T cells
NK cells
T cells CD8
NKT cells
T cells CD8
Cancer LumB SC
Cycling Myeloid
PVL Differentiated
Cancer Basal SC
Cancer Cycling
Myoepithelial
NKT cells
PVL Differentiated
Plasmablasts
Endothelial ACKR1
Luminal Progenitors
Mature Luminal
Cancer Her2 SC
B cells Memory
Cancer Basal SC
Cycling T cells
Myoepithelial
NK cells
T cells CD4
Cancer LumA SC
Cancer LumB SC
Cancer Cycling
Plasmablasts
Role
Role
source
target
Relative Frequency
0.3
0.2
0.1
0
Resource
Baccin2019
CellCall
CellChatDB
Cellinker
CellPhoneDB
CellTalkDB
ConnDB2020
EMBRACE
Guide2Pharma
HPMR
ICELLNET
iTALK
Kirouac2010
LRdb
OmniPath
Ramilowski
Method
CellChat
CellPhoneDB
Connectome
NATMI
SingleCellSignalR

D)

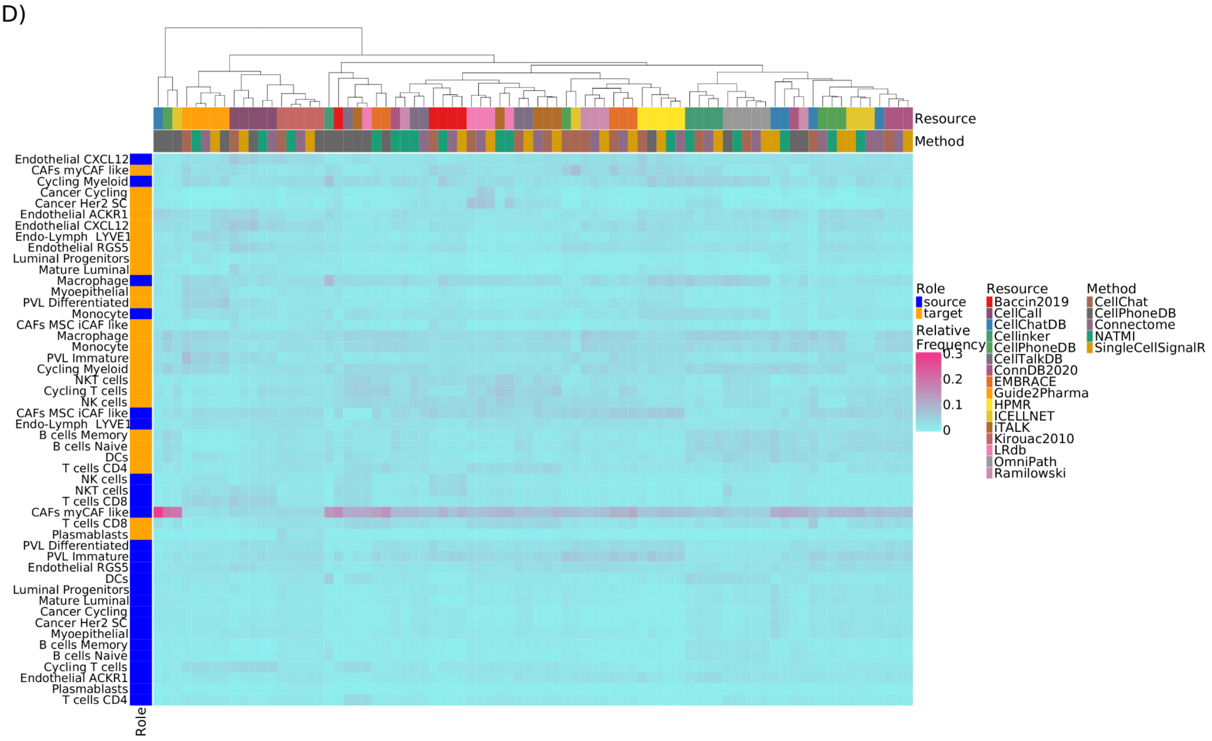
Resource
Method
Endothelial CXCL12
CAFs myCAF like
Cycling Myeloid
Cancer Cycling
Cancer Her2 SC
Endothelial ACKR1
Endothelial CXCL12
Endo-Lymph_LYVE1
Endothelial RGS5
Luminal Progenitors
Mature Luminal
Macrophage
Myoepithelial
PVL Differentiated
Monocyte
CAFs MSC iCAF like
Macrophage
Monocyte
PVL Immature
Cycling Myeloid
NKT cells
Cycling T cells
NK cells
CAFs MSC iCAF like
Endo-Lymph_LYVE1
B cells Memory
B cells Naive
DCs
T cells CD4
NK cells
NKT cells
T cells CD8
CAFs myCAF like
T cells CD8
Plasmablasts
PVL Differentiated
PVL Immature
Endothelial RGS5
DCs
Luminal Progenitors
Mature Luminal
Cancer Cycling
Cancer Her2 SC
Myoepithelial
B cells Memory
B cells Naive
Cycling T cells
Endothelial ACKR1
Plasmablasts
T cells CD4
Role
Role
source
target
Relative Frequency
0.3
0.2
0.1
0
Resource
Baccin2019
CellCall
CellChatDB
Cellinker
CellPhoneDB
CellTalkDB
ConnDB2020
EMBRACE
Guide2Pharma
HPMR
ICELLNET
iTALK
Kirouac2010
LRdb
OmniPath
Ramilowski
Method
CellChat
CellPhoneDB
Connectome
NATMI
SingleCellSignalR

E)

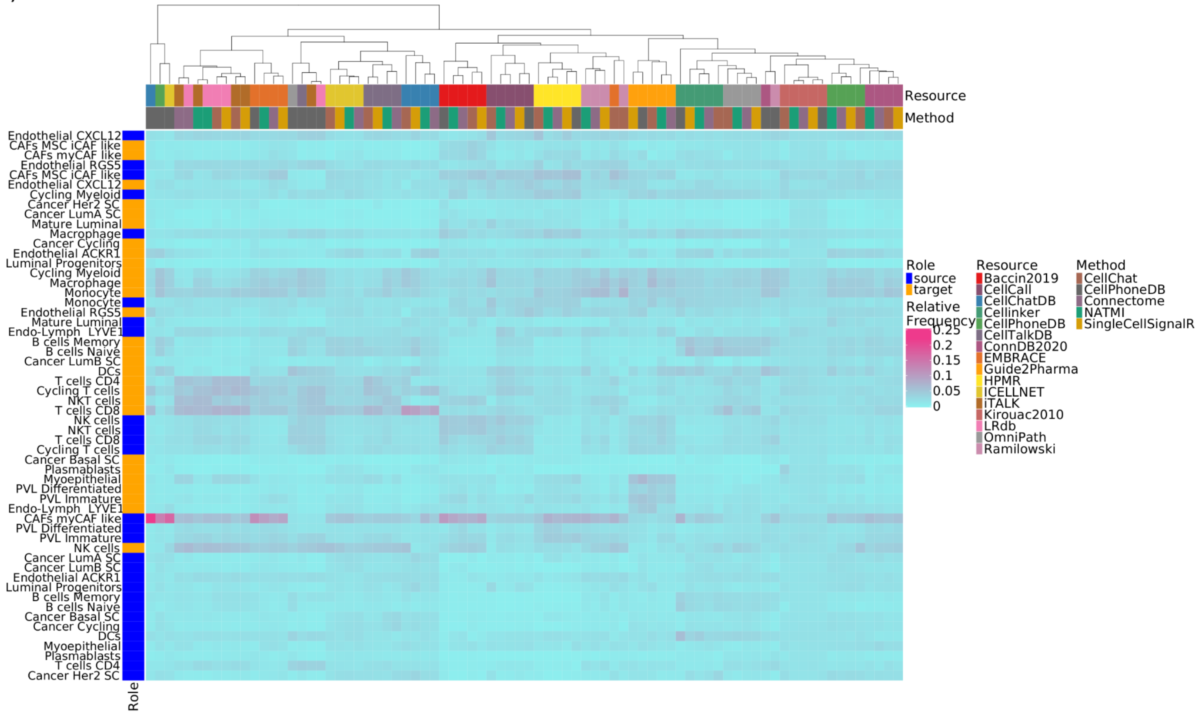
Resource
Method
Endothelial CXCL12
CAFs MSC iCAF like
CAFs myCAF like
Endothelial RGS5
CAFs MSC iCAF like
Endothelial CXCL12
Cycling Myeloid
Cancer Her2 SC
Cancer LumA SC
Mature Luminal
Macrophage
Cancer Cycling
Endothelial ACKR1
Luminal Progenitors
Cycling Myeloid
Macrophage
Monocyte
Monocyte
Endothelial RGS5
Mature Luminal
Endo-Lymph LYVE1
B cells Memory
B cells Naive
Cancer LumB SC
DCs
T cells CD4
Cycling T cells
NKT cells
T cells CD8
NK cells
NKT cells
T cells CD8
Cycling T cells
Cancer Basal SC
Plasmablasts
Myoepithelial
PVL Differentiated
PVL Immature
Endo-Lymph LYVE1
CAFs myCAF like
PVL Differentiated
PVL Immature
NK cells
Cancer LumA SC
Cancer LumB SC
Endothelial ACKR1
Luminal Progenitors
B cells Memory
B cells Naive
Cancer Basal SC
Cancer Cycling
DCs
Myoepithelial
Plasmablasts
T cells CD4
Cancer Her2 SC
Role
Role
source
target
Relative Frequency
0.25
0.2
0.15
0.1
0.05
0
Resource
Baccin2019
CellCall
CellChatDB
Cellinker
CellPhoneDB
CellTalkDB
ConnDB2020
EMBRACE
Guide2Pharma
HPMR
ICELLNET
iTALK
Kirouac2010
LRdb
OmniPath
Ramilowski
Method
CellChat
CellPhoneDB
Connectome
NATMI
SingleCellSignalR

F)

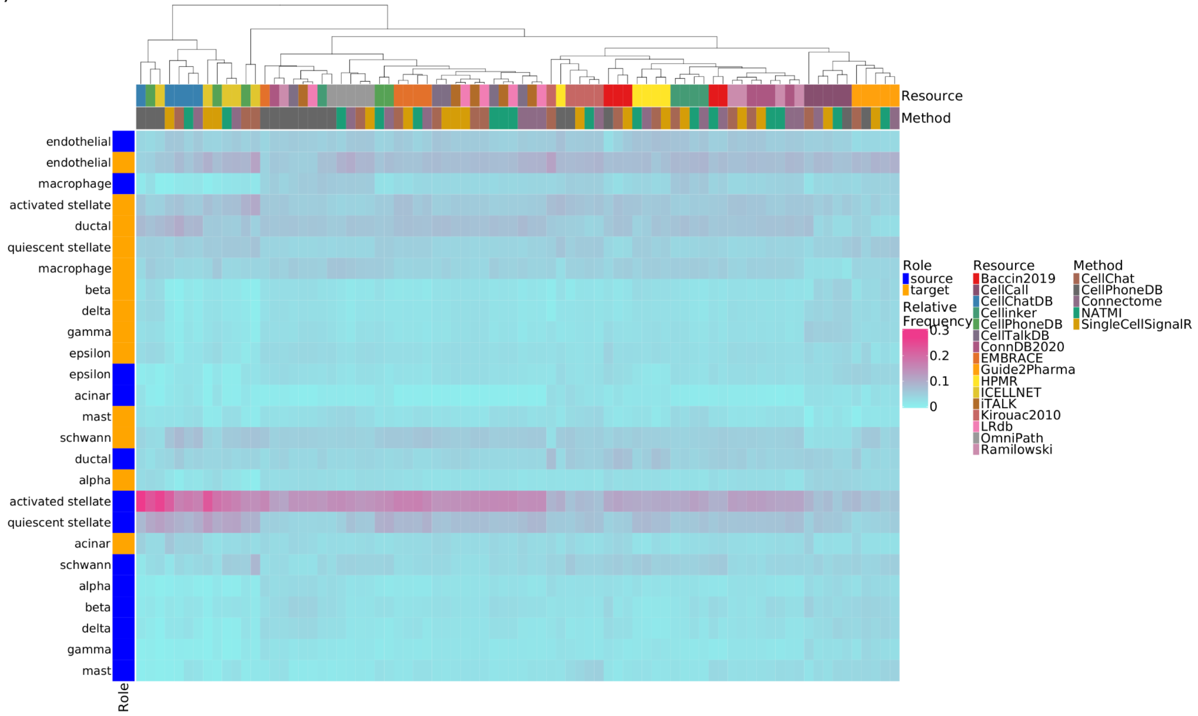
Resource
Method
endothelial
endothelial
macrophage
activated stellate
ductal
quiescent stellate
macrophage
beta
delta
gamma
epsilon
epsilon
acinar
mast
schwann
ductal
alpha
activated stellate
quiescent stellate
acinar
schwann
alpha
beta
delta
gamma
mast
Role
Role
source
target
Relative Frequency
0.3
0.2
0.1
0
Resource
Baccin2019
CellCall
CellChatDB
Cellinker
CellPhoneDB
CellTalkDB
ConnDB2020
EMBRACE
Guide2Pharma
HPMR
ICELLNET
iTALK
Kirouac2010
LRdb
OmniPath
Ramilowski
Method
CellChat
CellPhoneDB
Connectome
NATMI
SingleCellSignalR

Supplementary Figure S24. *Relative strength of interactions per cell type estimated using for* ***A)*** *CBMCs,* ***B)*** *Colorectal cancer,* ***C)*** *ER+ breast cancer,* ***D)*** *HER2+ breast cancer,* ***E)*** *Triple negative breast cancer, and* ***F)*** *Pancreatic Islet datasets.*

A)

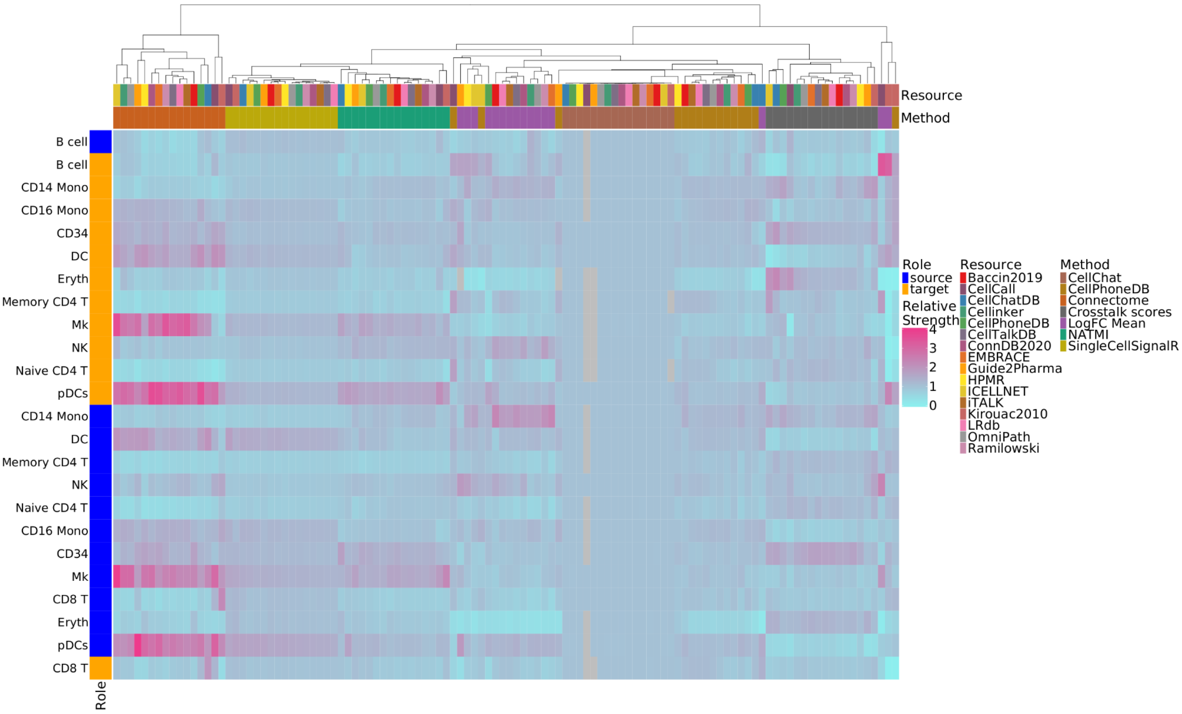

B)

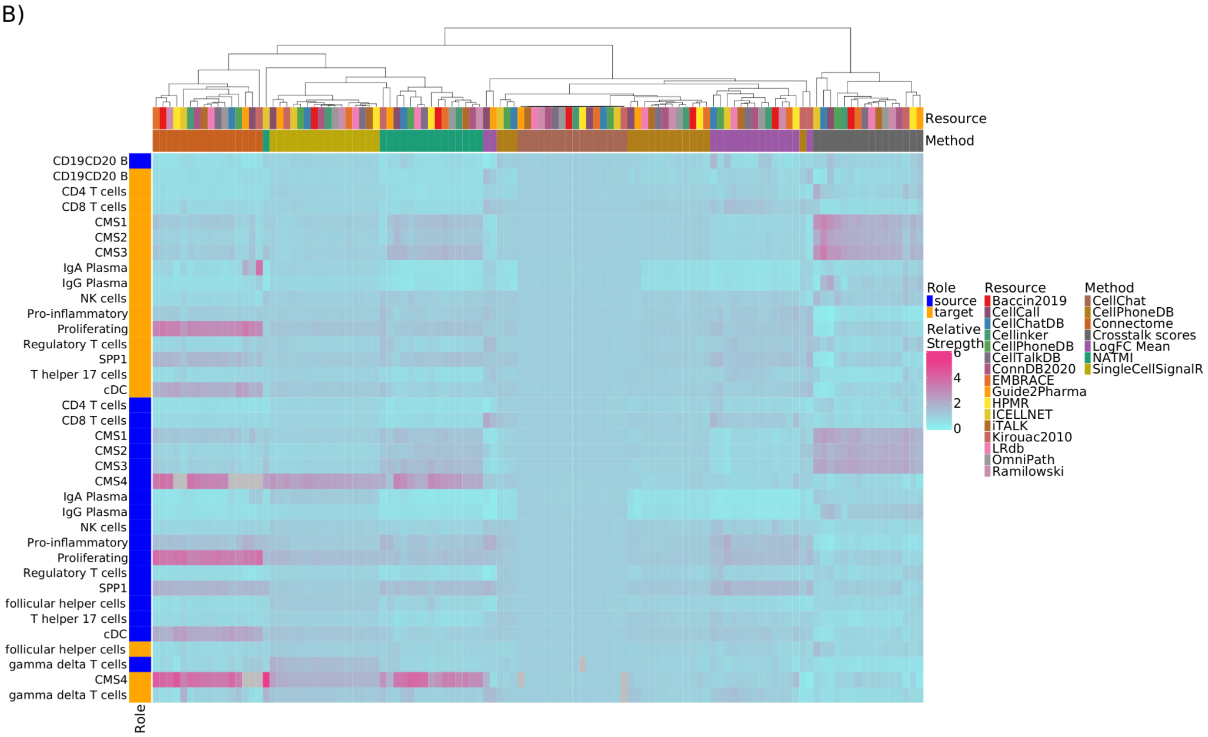

C)

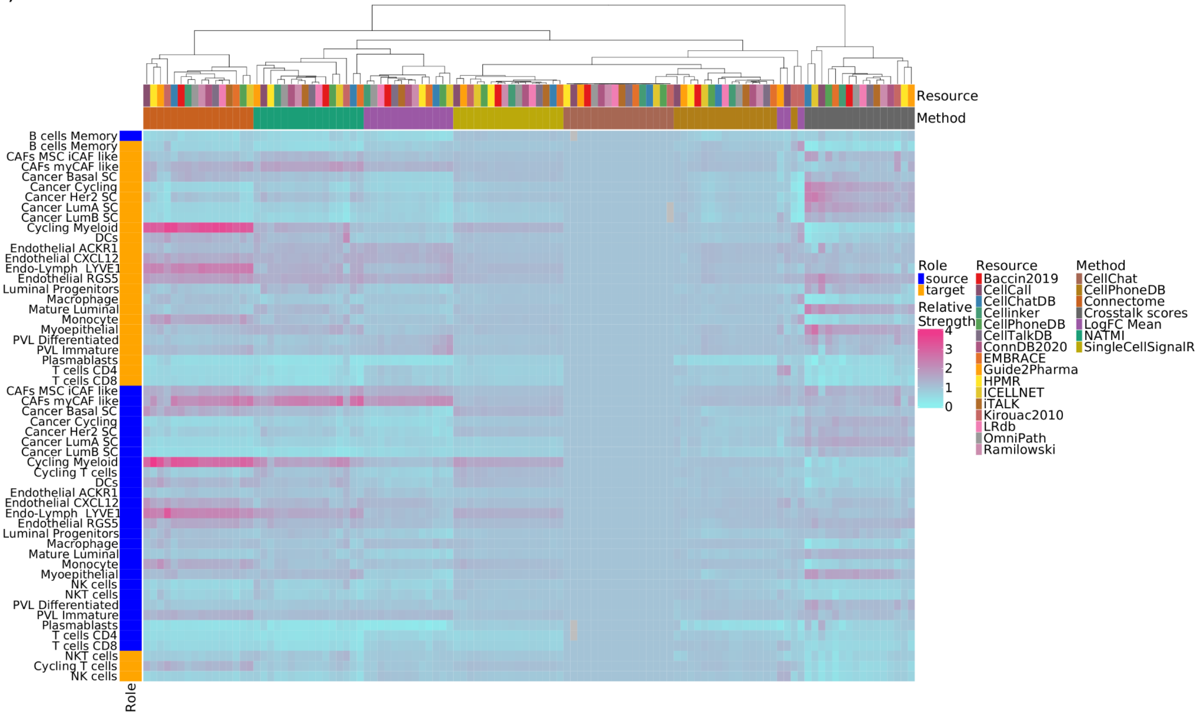
Resource
Method
B cells Memory
B cells Memory
CAFs MSC iCAF like
CAFs myCAF like
Cancer Basal SC
Cancer Cycling
Cancer Her2 SC
Cancer LumA SC
Cancer LumB SC
Cycling Myeloid
DCs
Endothelial ACKR1
Endothelial CXCL12
Endo-Lymph LYVE1
Endothelial RGS5
Luminal Progenitors
Macrophage
Mature Luminal
Monocyte
Myoepithelial
PVL Differentiated
PVL Immature
Plasmablasts
T cells CD4
T cells CD8
CAFs MSC iCAF like
CAFs myCAF like
Cancer Basal SC
Cancer Cycling
Cancer Her2 SC
Cancer LumA SC
Cancer LumB SC
Cycling Myeloid
Cycling T cells
DCs
Endothelial ACKR1
Endothelial CXCL12
Endo-Lymph LYVE1
Endothelial RGS5
Luminal Progenitors
Macrophage
Mature Luminal
Monocyte
Myoepithelial
NK cells
NKT cells
PVL Differentiated
PVL Immature
Plasmablasts
T cells CD4
T cells CD8
NKT cells
Cycling T cells
NK cells
Role
Role
source
target
Relative Strength
4
3
2
1
0
Resource
Baccin2019
CellCall
CellChatDB
Cellinker
CellPhoneDB
CellTalkDB
ConnDB2020
EMBRACE
Guide2Pharma
HPMR
ICELLNET
iTALK
Kirouac2010
LRdb
OmniPath
Ramilowski
Method
CellChat
CellPhoneDB
Connectome
Crosstalk scores
LogFC Mean
NATMI
SingleCellSignalR

D)

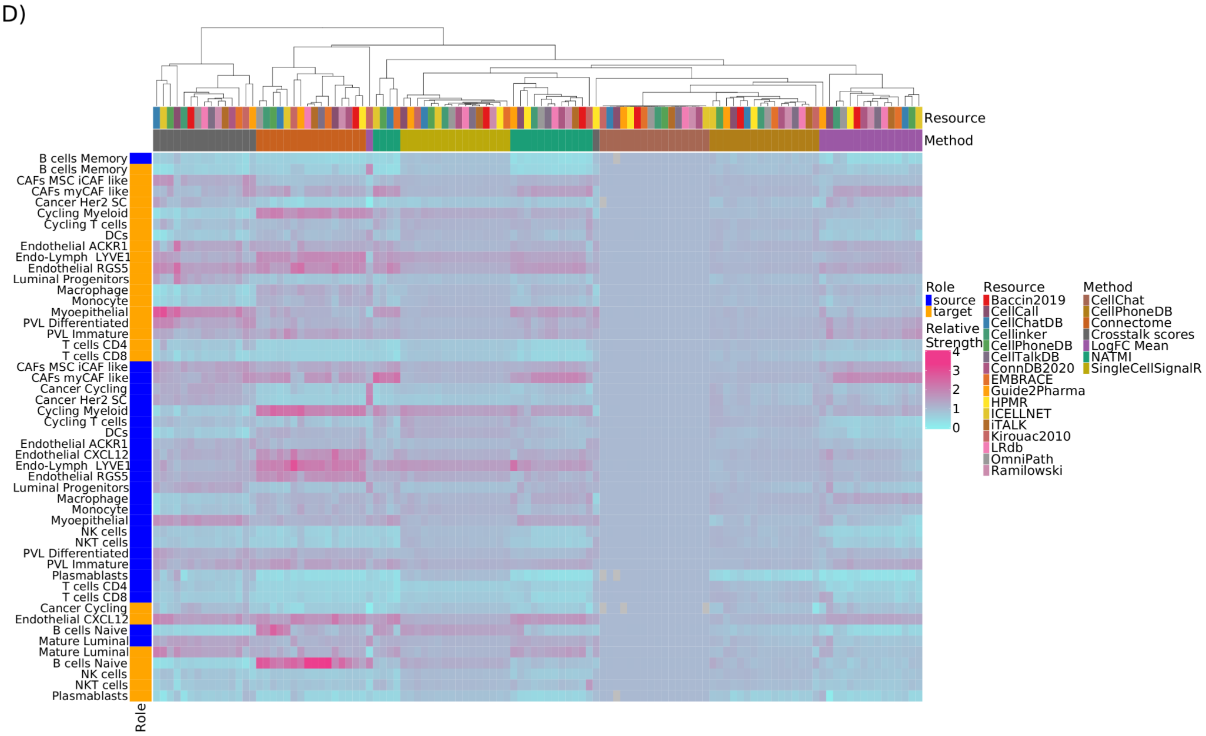
Resource
Method
B cells Memory
B cells Memory
CAFs MSC iCAF like
CAFs myCAF like
Cancer Her2 SC
Cycling Myeloid
Cycling T cells
DCs
Endothelial ACKR1
Endo-Lymph LYVE1
Endothelial RGS5
Luminal Progenitors
Macrophage
Monocyte
Myoepithelial
PVL Differentiated
PVL Immature
T cells CD4
T cells CD8
CAFs MSC iCAF like
CAFs myCAF like
Cancer Cycling
Cancer Her2 SC
Cycling Myeloid
Cycling T cells
DCs
Endothelial ACKR1
Endothelial CXCL12
Endo-Lymph LYVE1
Endothelial RGS5
Luminal Progenitors
Macrophage
Monocyte
Myoepithelial
NK cells
NKT cells
PVL Differentiated
PVL Immature
Plasmablasts
T cells CD4
T cells CD8
Cancer Cycling
Endothelial CXCL12
B cells Naive
Mature Luminal
Mature Luminal
B cells Naive
NK cells
NKT cells
Plasmablasts
Role
Role
source
target
Relative Strength
4
3
2
1
0
Resource
Baccin2019
CellCall
CellChatDB
Cellinker
CellPhoneDB
CellTalkDB
ConnDB2020
EMBRACE
Guide2Pharma
HPMR
ICELLNET
iTALK
Kirouac2010
LRdb
OmniPath
Ramilowski
Method
CellChat
CellPhoneDB
Connectome
Crosstalk scores
LogFC Mean
NATMI
SingleCellSignalR

E)

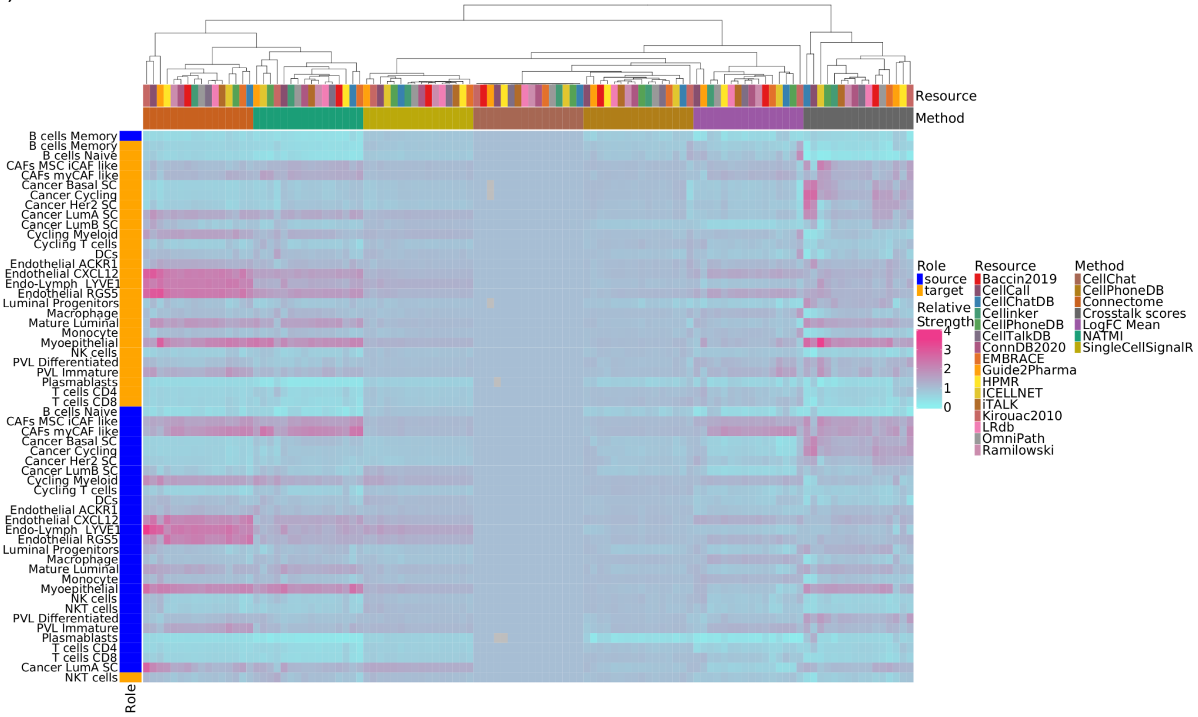

Resource
Method
B cells Memory
B cells Memory
B cells Naive
CAFs MSC iCAF like
CAFs myCAF like
Cancer Basal SC
Cancer Cycling
Cancer Her2 SC
Cancer LumA SC
Cancer LumB SC
Cycling Myeloid
Cycling T cells
DCs
Endothelial ACKR1
Endothelial CXCL12
Endo-Lymph_LYVE1
Endothelial RGS5
Luminal Progenitors
Macrophage
Mature Luminal
Monocyte
Myoepithelial
NK cells
PVL Differentiated
PVL Immature
Plasmablasts
T cells CD4
T cells CD8
B cells Naive
CAFs MSC iCAF like
CAFs myCAF like
Cancer Basal SC
Cancer Cycling
Cancer Her2 SC
Cancer LumB SC
Cycling Myeloid
Cycling T cells
DCs
Endothelial ACKR1
Endothelial CXCL12
Endo-Lymph_LYVE1
Endothelial RGS5
Luminal Progenitors
Macrophage
Mature Luminal
Monocyte
Myoepithelial
NK cells
NKT cells
PVL Differentiated
PVL Immature
Plasmablasts
T cells CD4
T cells CD8
Cancer LumA SC
NKT cells
Role
Role
source
target
Relative Strength
4
3
2
1
0
Resource
Baccin2019
CellCall
CellChatDB
Cellinker
CellPhoneDB
CellTalkDB
ConnDB2020
EMBRACE
Guide2Pharma
HPMR
ICELLNET
iTALK
Kirouac2010
LRdb
OmniPath
Ramilowski
Method
CellChat
CellPhoneDB
Connectome
Crosstalk scores
LogFC Mean
NATMI
SingleCellSignalR

F)

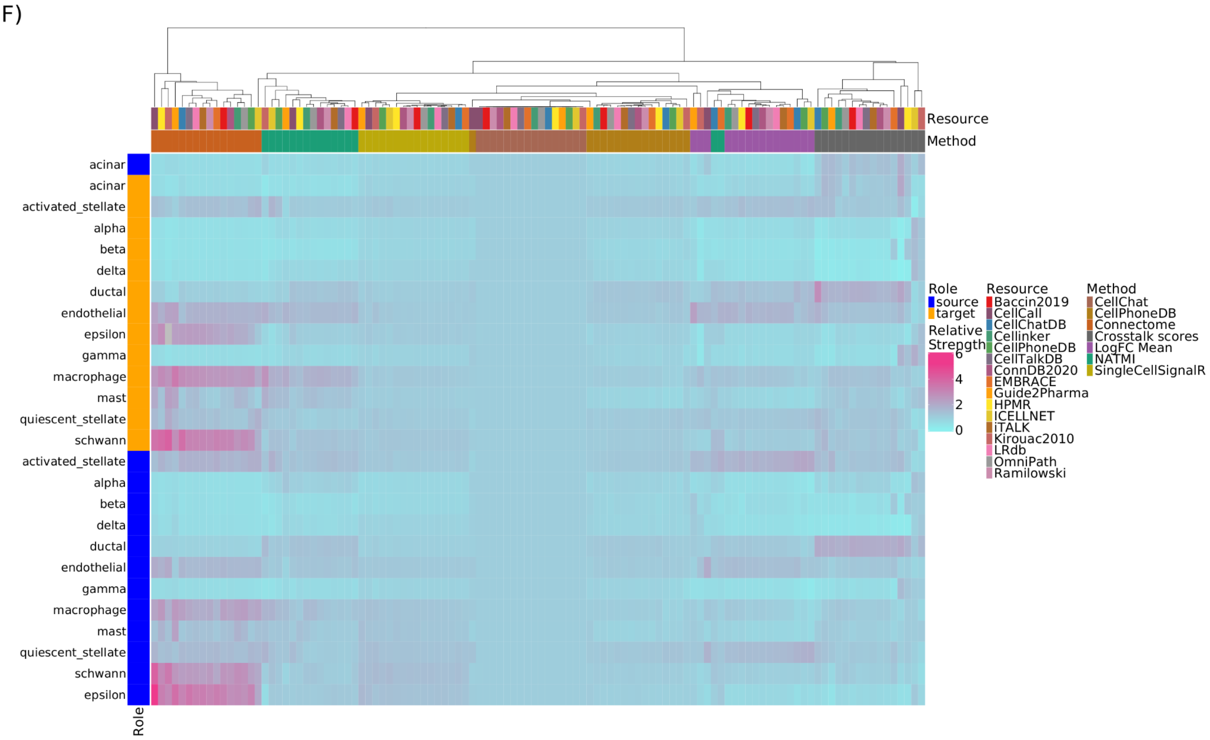

Resource
Method
acinar
acinar
activated_stellate
alpha
beta
delta
ductal
endothelial
epsilon
gamma
macrophage
mast
quiescent_stellate
schwann
activated_stellate
alpha
beta
delta
ductal
endothelial
gamma
macrophage
mast
quiescent_stellate
schwann
epsilon
Role
Role
source
target
Relative Strength
6
4
2
0
Resource
Baccin2019
CellCall
CellChatDB
Cellinker
CellPhoneDB
CellTalkDB
ConnDB2020
EMBRACE
Guide2Pharma
HPMR
ICELLNET
iTALK
Kirouac2010
LRdb
OmniPath
Ramilowski
Method
CellChat
CellPhoneDB
Connectome
Crosstalk scores
LogFC Mean
NATMI
SingleCellSignalR

Supplementary Figure S25. *Relative strength of interactions per cell type estimated using the non-specific interaction scores for* ***A)*** *CBMCs,* ***B)*** *Colorectal cancer,* ***C)*** *ER+ breast cancer,* ***D)*** *HER2+ breast cancer,* ***E)*** *Triple negative breast cancer, and* ***F)*** *Pancreatic Islet datasets.*

A)

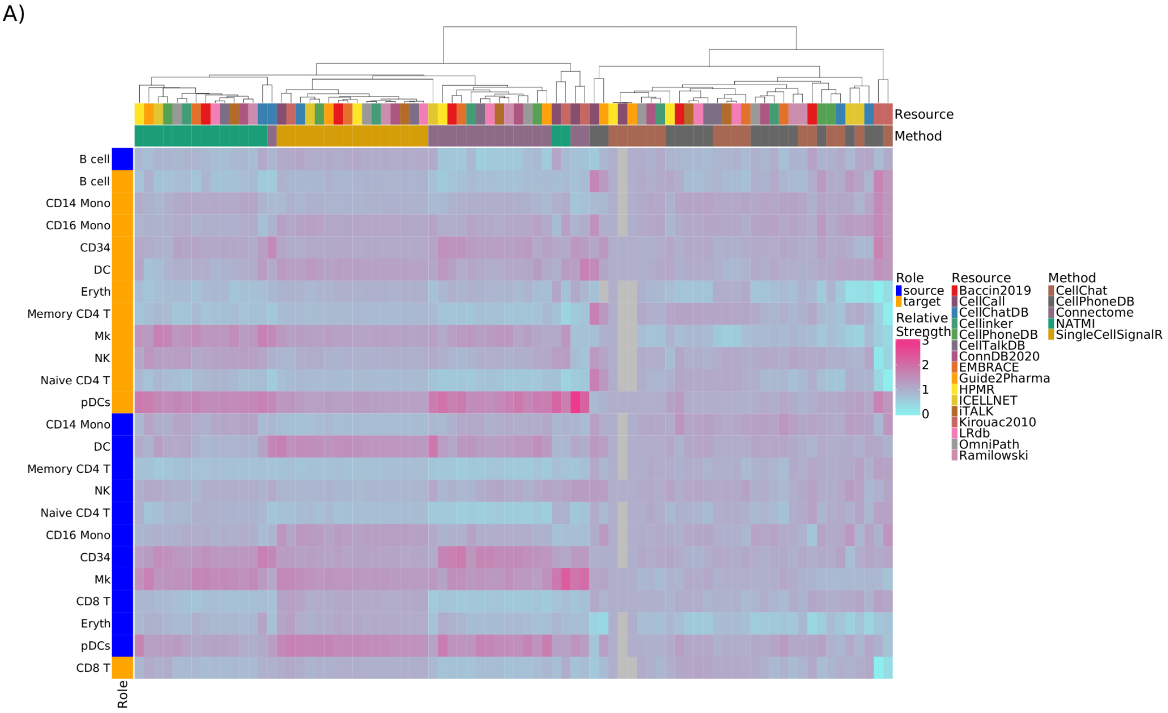

B)

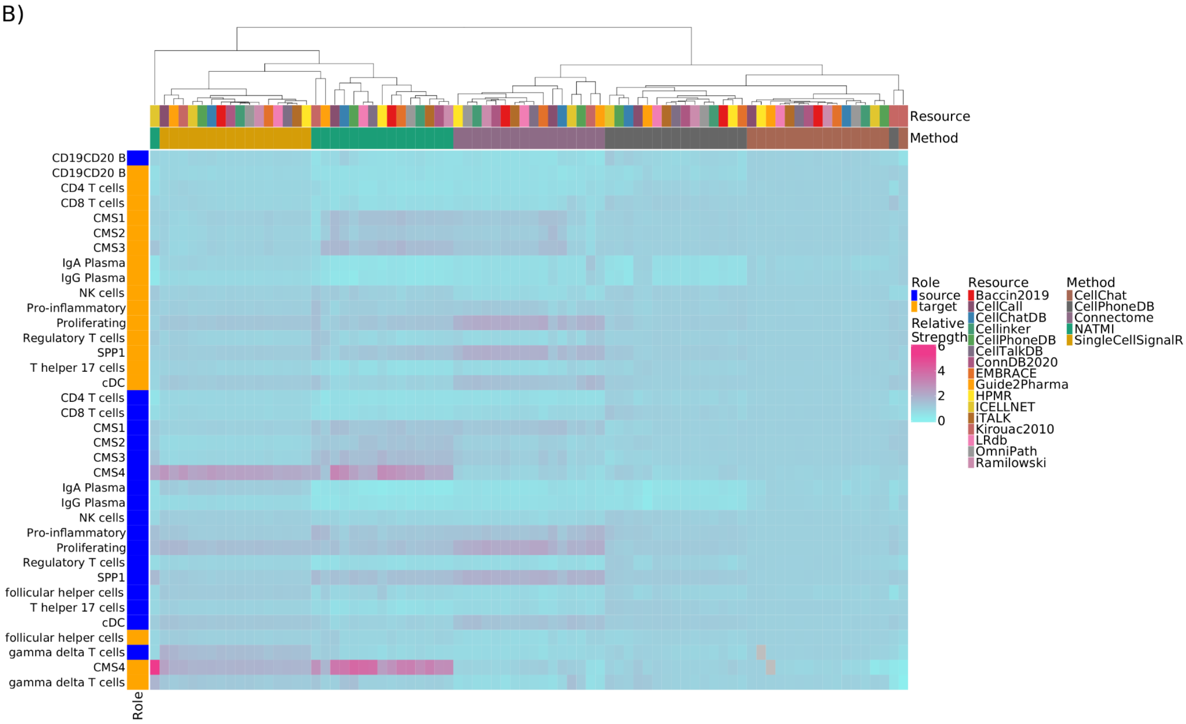

C)

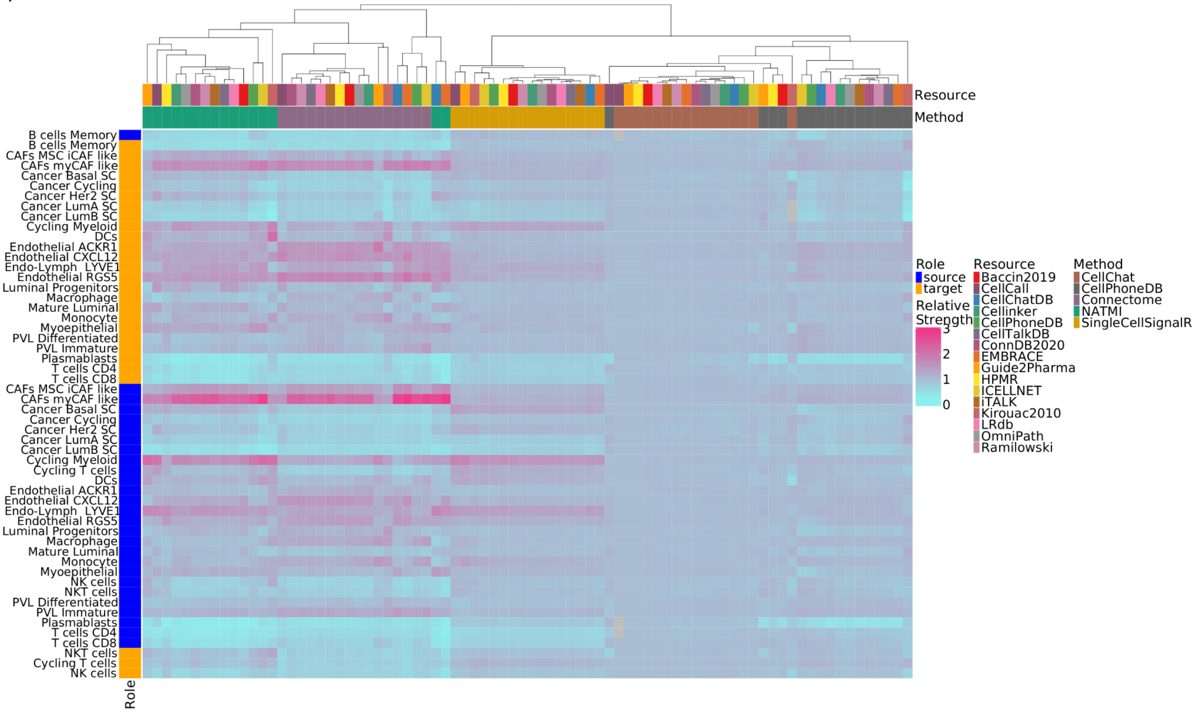
Resource
Method
B cells Memory
B cells Memory
CAFs MSC iCAF like
CAFs myCAF like
Cancer Basal SC
Cancer Cycling
Cancer Her2 SC
Cancer LumA SC
Cancer LumB SC
Cycling Myeloid
DCs
Endothelial ACKR1
Endothelial CXCL12
Endo-Lymph_LYVE1
Endothelial RGS5
Luminal Progenitors
Macrophage
Mature Luminal
Monocyte
Myoepithelial
PVL Differentiated
PVL Immature
Plasmablasts
T cells CD4
T cells CD8
CAFs MSC iCAF like
CAFs myCAF like
Cancer Basal SC
Cancer Cycling
Cancer Her2 SC
Cancer LumA SC
Cancer LumB SC
Cycling Myeloid
Cycling T cells
DCs
Endothelial ACKR1
Endothelial CXCL12
Endo-Lymph_LYVE1
Endothelial RGS5
Luminal Progenitors
Macrophage
Mature Luminal
Monocyte
Myoepithelial
NK cells
NKT cells
PVL Differentiated
PVL Immature
Plasmablasts
T cells CD4
T cells CD8
NKT cells
Cycling T cells
NK cells
Role
Role
source
target
Relative Strength
3
2
1
0
Resource
Baccin2019
CellCall
CellChatDB
Cellinker
CellPhoneDB
CellTalkDB
ConnDB2020
EMBRACE
Guide2Pharma
HPMR
ICELLNET
iTALK
Kirouac2010
LRdb
OmniPath
Ramilowski
Method
CellChat
CellPhoneDB
Connectome
NATMI
SingleCellSignalR

D)

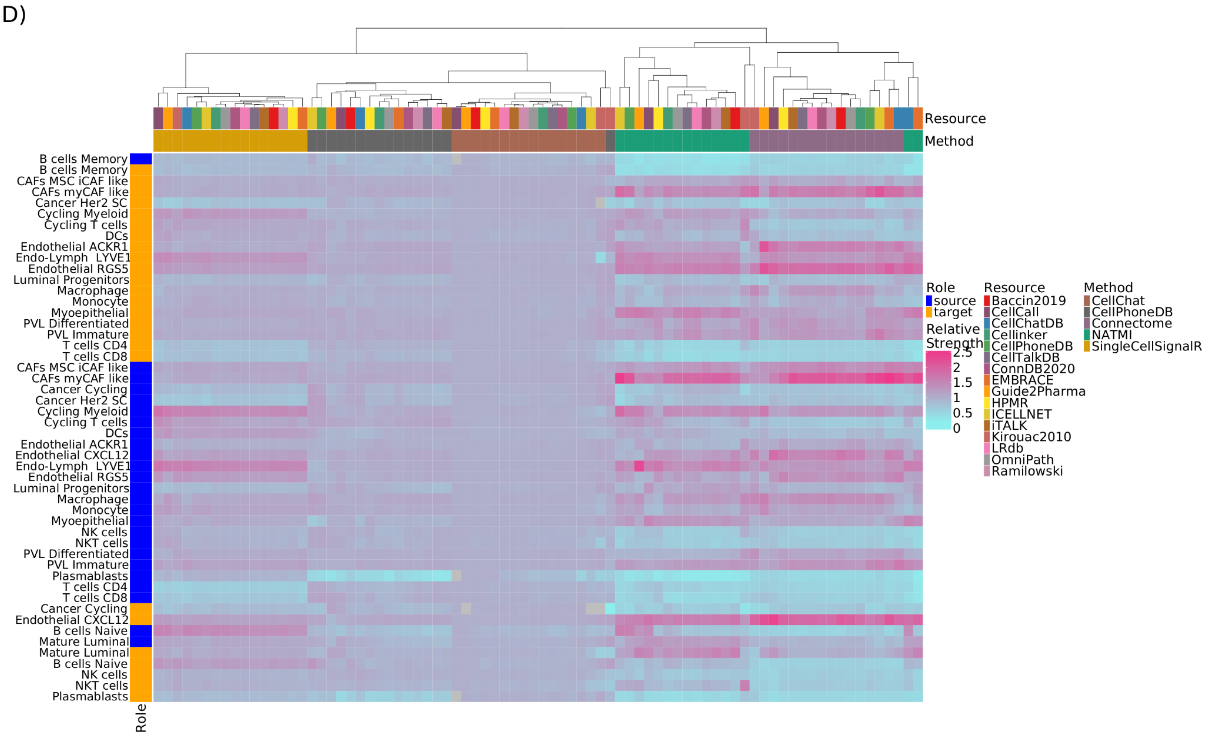
Resource
Method
B cells Memory
B cells Memory
CAFs MSC iCAF like
CAFs myCAF like
Cancer Her2 SC
Cycling Myeloid
Cycling T cells
DCs
Endothelial ACKR1
Endo-Lymph_LYVE1
Endothelial RGS5
Luminal Progenitors
Macrophage
Monocyte
Myoepithelial
PVL Differentiated
PVL Immature
T cells CD4
T cells CD8
CAFs MSC iCAF like
CAFs myCAF like
Cancer Cycling
Cancer Her2 SC
Cycling Myeloid
Cycling T cells
DCs
Endothelial ACKR1
Endothelial CXCL12
Endo-Lymph_LYVE1
Endothelial RGS5
Luminal Progenitors
Macrophage
Monocyte
Myoepithelial
NK cells
NKT cells
PVL Differentiated
PVL Immature
Plasmablasts
T cells CD4
T cells CD8
Cancer Cycling
Endothelial CXCL12
B cells Naive
Mature Luminal
Mature Luminal
B cells Naive
NK cells
NKT cells
Plasmablasts
Role
Role
source
target
Relative Strength
2.5
2
1.5
1
0.5
0
Resource
Baccin2019
CellCall
CellChatDB
Cellinker
CellPhoneDB
CellTalkDB
ConnDB2020
EMBRACE
Guide2Pharma
HPMR
ICELLNET
iTALK
Kirouac2010
LRdb
OmniPath
Ramilowski
Method
CellChat
CellPhoneDB
Connectome
NATMI
SingleCellSignalR

E)

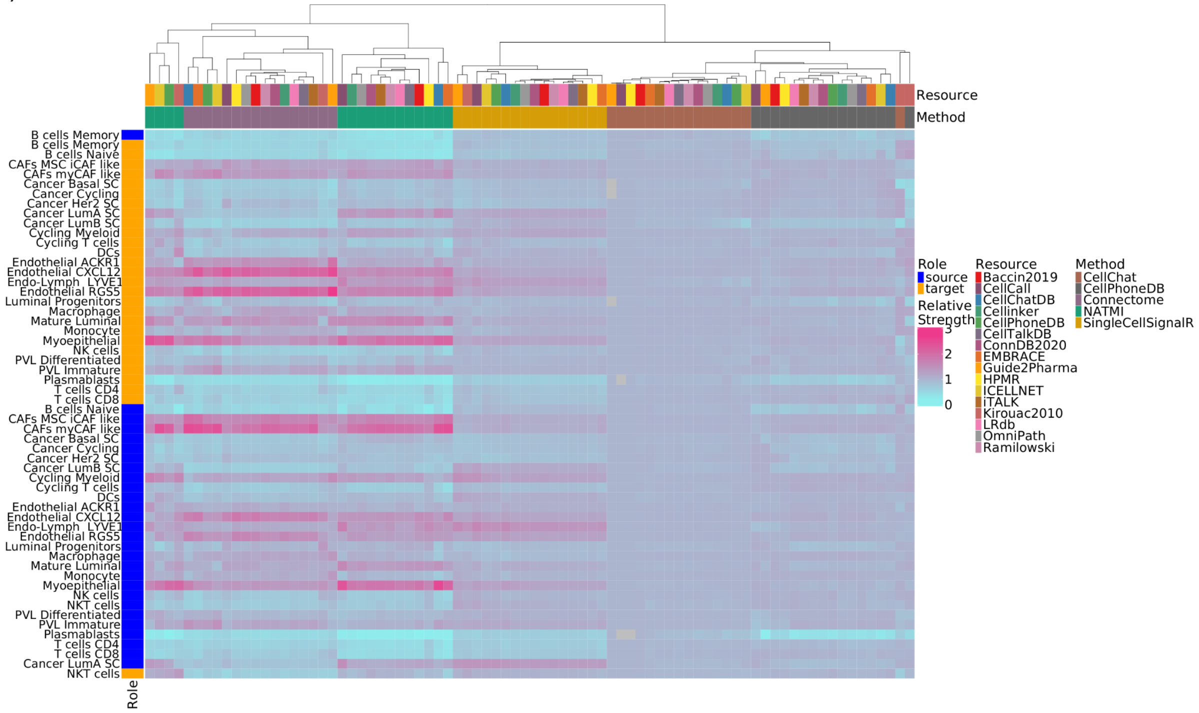

Resource
Method
Role
source
target
Relative Strength
3
2
1
0
Baccin2019
CellCall
CellChatDB
Cellinker
CellPhoneDB
CellTalkDB
ConnDB2020
EMBRACE
Guide2Pharma
HPMR
ICELLNET
iTALK
Kirouac2010
LRdb
OmniPath
Ramilowski
CellChat
CellPhoneDB
Connectome
NATMI
SingleCellSignalR
B cells Memory
B cells Memory
B cells Naive
CAFs MSC iCAF like
CAFs myCAF like
Cancer Basal SC
Cancer Cycling
Cancer Her2 SC
Cancer LumA SC
Cancer LumB SC
Cycling Myeloid
Cycling T cells
DCs
Endothelial ACKR1
Endothelial CXCL12
Endo-Lymph LYVE1
Endothelial RGS5
Luminal Progenitors
Macrophage
Mature Luminal
Monocyte
Myoepithelial
NK cells
PVL Differentiated
PVL Immature
Plasmablasts
T cells CD4
T cells CD8
B cells Naive
CAFs MSC iCAF like
CAFs myCAF like
Cancer Basal SC
Cancer Cycling
Cancer Her2 SC
Cancer LumB SC
Cycling Myeloid
Cycling T cells
DCs
Endothelial ACKR1
Endothelial CXCL12
Endo-Lymph LYVE1
Endothelial RGS5
Luminal Progenitors
Macrophage
Mature Luminal
Monocyte
Myoepithelial
NK cells
NKT cells
PVL Differentiated
PVL Immature
Plasmablasts
T cells CD4
T cells CD8
Cancer LumA SC
NKT cells
Role

F)

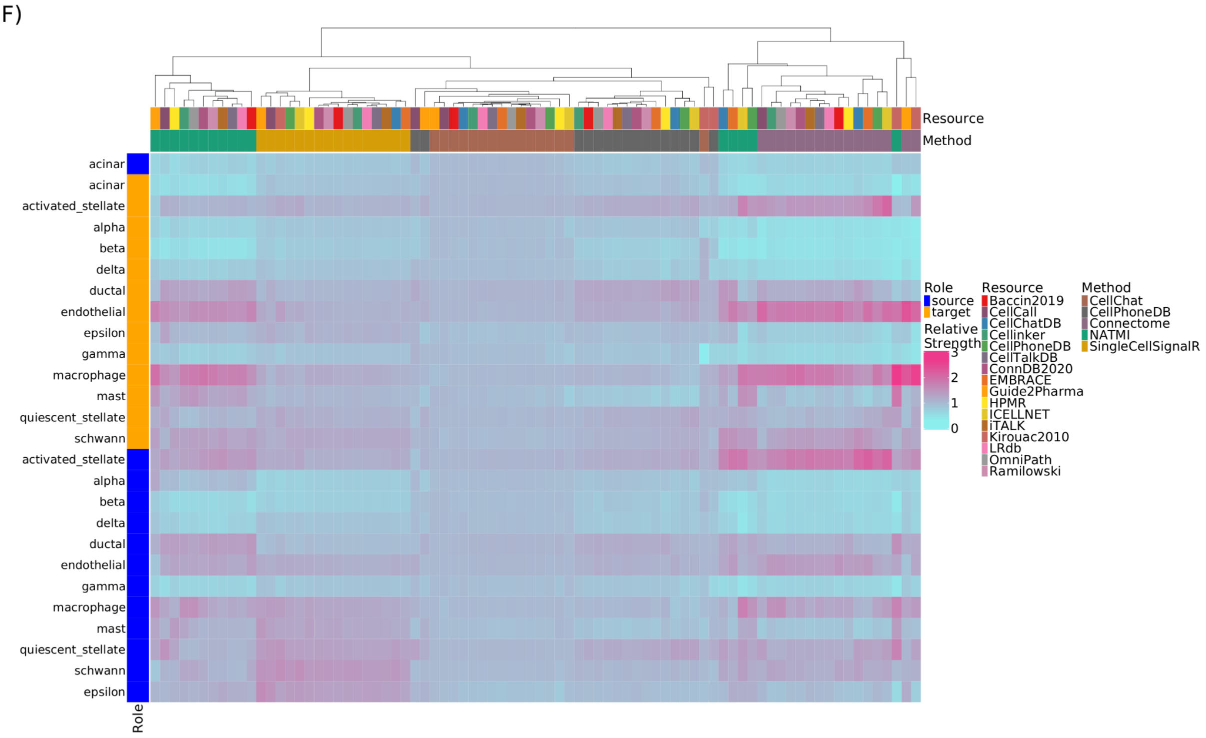

Resource
Method
Role
source
target
Relative Strength
3
2
1
0
Baccin2019
CellCall
CellChatDB
Cellinker
CellPhoneDB
CellTalkDB
ConnDB2020
EMBRACE
Guide2Pharma
HPMR
ICELLNET
iTALK
Kirouac2010
LRdb
OmniPath
Ramilowski
CellChat
CellPhoneDB
Connectome
NATMI
SingleCellSignalR
acinar
acinar
activated_stellate
alpha
beta
delta
ductal
endothelial
epsilon
gamma
macrophage
mast
quiescent_stellate
schwann
activated_stellate
alpha
beta
delta
ductal
endothelial
gamma
macrophage
mast
quiescent_stellate
schwann
epsilon
Role

Supplementary Figure S26. *Agreement with receptor protein specificity using **A)** CellPhoneDB and CellChat's composite scores, and **B)** p-values alone. Each method's performance was estimated independently, i.e. only using the interactions predicted following the preprocessing steps of each method.*

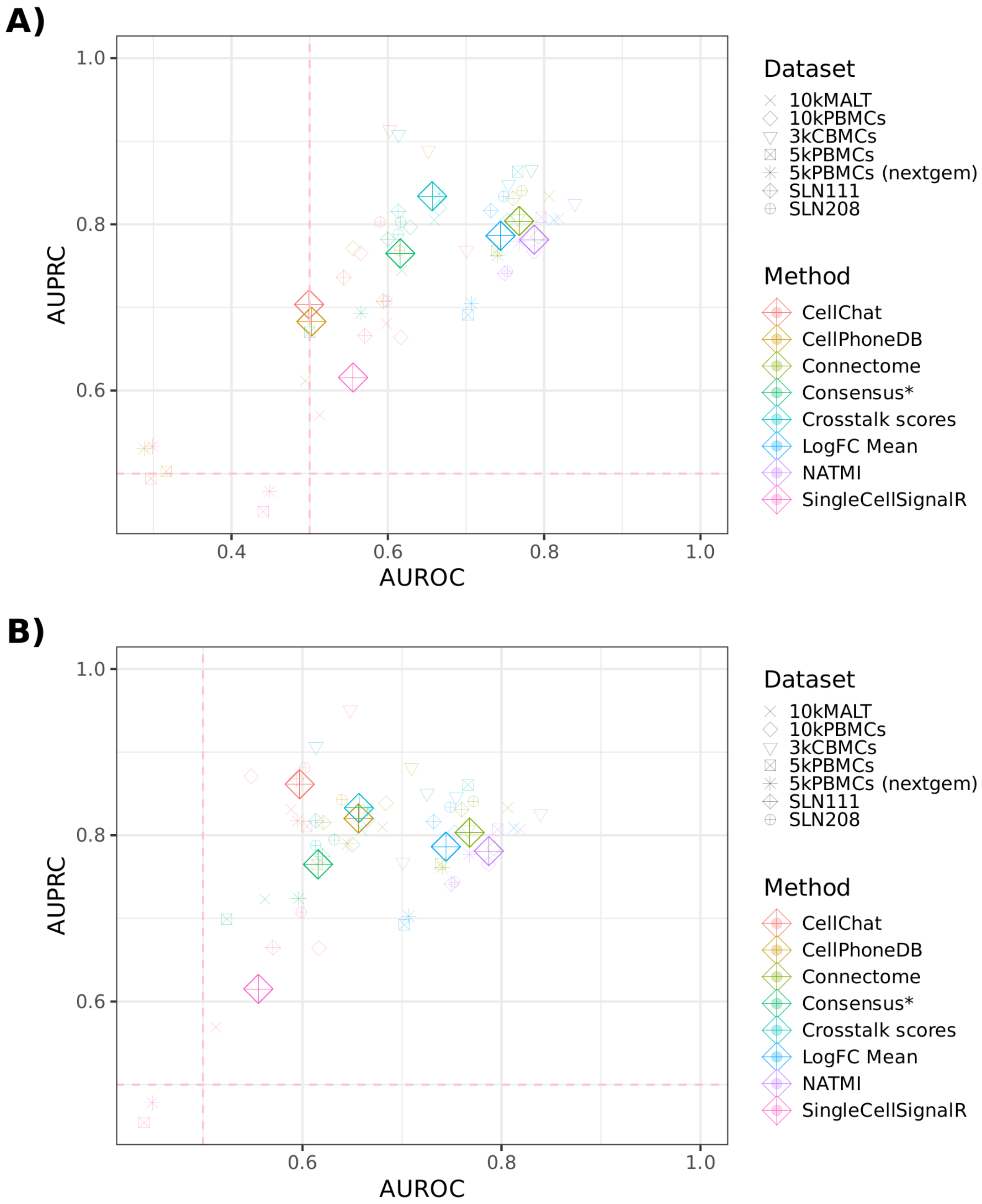

Supplementary table 1. Unique Transmitters, Receivers, and interactions in each resource. We defined unique–interactions, receivers and transmitters between the CCC resources if they could be found in only one of the resources.

Resource	Transmitters	Receivers	Interactions
Baccin2019	5.71%	4.93%	10.60%
CellCall	1.81%	0.51%	3.14%
CellChatDB	4.25%	3.78%	20.76%
Cellinker	30.34%	24.93%	39.30%
CellPhoneDB	2.99%	6.94%	10.68%
CellTalkDB	2.94%	5.24%	5.53%
ConnDB2020	6.12%	3.51%	8.04%
EMBRACE	0.43%	0.00%	0.53%
GuidePharm	0.68%	0.41%	5.52%
HPMR	8.22%	12.75%	18.12%
ICELLNET	0.31%	0.40%	2.25%
iTALK	0.00%	0.00%	0.11%
Kirouac2010	4.20%	0.00%	10.53%
LRdb	0.75%	0.00%	1.51%
OmniPath	4.09%	3.95%	8.45%
Ramilowski	0.00%	0.00%	0.00%
Total	6.35%	5.74%	10.35%

Supplementary table 2. Cell–cell communication resources - additional information.

Resource	Further curation	Sources	Interactions*	Original curation † §	Overlap with curated set✦§
Baccin2019/**(a)**[1]	Murine identifiers (only), Multimeric complexes	Ramilowski2015, KEGG REACTOME, Literature	1,625	No‡	82%
CellCall [2]	Multimeric complexes, murine identifiers, Interactions downstream transcription factors (from KEGG), transcription factor regulons.	ConnectomeDB2020, CellLinker, CellTalkDB, CellChatDB, STRING	1,123	No	51%
CellChatDB[3]	Multimeric complexes, 229 signaling pathway families, agonists and antagonists, co-receptors, localisations, Murine identifiers	KEGG, Literature	1,912	Yes	84%
Cellinker [4]	Multimeric complexes, murine identifiers, sMOL ligands, intercellular communication roles	DLRP, HPMR, CellPhoneDB, Guide2Pharma, Literature	3,701	Yes	71%
CellPhoneDB [5–7]	Multimeric complexes, intercellular communication roles	Guide2Pharma, I2D, IntAct, UniProt, Literature, HPIDB	1,219	Yes	90%
CellTalkDB[8]	Murine identifiers	STRING, Literature	3,392	Yes	100%
ConnectomeDB2020[9]	Intercellular communication roles	Ramilowski2015, CellphoneDB, Baccin2019, LRdb, ICELLNET, Literature	2,266	Yes†	99%
EMBRACE**(a)**[10]	Murine identifiers	Ramilowski2015	1,639	No	97%
Guide2Pharma**(b)**[11]	-	Literature	665	Yes	100%
HPMR[12]	-	Literature	596	Yes	100%
ICELLNET[13]	Multimeric complexes, Signalling families, Cytokine-focus	STRING, Ingenuity, BioGRID, Reactome, CellPhoneDB	738	Yes	95%
iTALK[14]	Ligand categories	Ramilowski2015, HPMR, Guide2Pharma, Graeber2001, Griffith2014, Cameron2003, Zhou2017, Auslander2018	2,566	No‡	96%
Kirouac2010[15]	-	Literature, COPE	152	Yes	100%

LRdb[16]	-	cellsignal.com, Ramilowski2015, Guide2Pharma, HPMR, HPRD, Reactome, UniProt, Literature	3,228	No‡	96%
Ramilowski2015[17]	-	DLRP, HPMR, IUPHAR, HPRD, STRING, Literature	1889	Yes†	99%
scConnect [18]	-	Guide2Pharma	479	No	92%

Asterisk **(*)**: All the resources above, except CellCall which was obtained directly via its Github page and converted to OmniPath format, were retrieved from the OmniPath database (https://omnipathdb.org/). Here, we show the number of human interactions for each CCC dedicated resource subsequent to dissociation of complexes into subunits.

Cross (✝): Does the resource contain original expert curated data? Here *expert curated* (synonyms: manually- or literature curated) means only the interactions curated in the context of cell-cell communication. We assessed the curation contents based on the publications, webpages, software manuals and data files.

Star (✦) We collected the curated interactions from all resources which contained any. Then for each resource we measured the overlap against the curated set. Many resources contribute to the curated set, or integrate other resources which are part of it, resulting in a bias in the overlaps.

Section sign (§) These columns aim to give insights on the status of curated CCC interactions, with limitations described in further footnotes. None of these attributes is an objective measure of the value or quality of the resources.

Double dagger (‡): Unclear whether the resource contains original curation effort, insufficient information in the description of methods or in the data

Dagger (†): connectomeDB2020 is an updated edition of Ramilowski2015

(a) Translated from murine identifiers to human, which accounts for the lower number of obtained interactions.

(b) Kept only the unique human-annotated interactions between transmitter and receiver proteins.

Supplementary table 3. Description of the different scoring settings used to compare the methods presented in this work.

	Composite#		Non-composite	Non-Specific
Method	*Used throughout the main text*		*Specificity alone for CellChat and CellPhoneDB*	*Scores which do not incorporate cell-pair specificity in interaction predictions*
CellChat	Probability	(Filtered by p-value =< 0.05)	p-values alone	Probability
CellPhoneDBv2	Truncated Mean	(Filtered by p-value =< 0.05)	p-values alone	Truncated Mean
Connectome	weight_scale		weight_scale	weight_norm
Crosstalk Scores	Crosstalk score		Crosstalk score	-
logFC Mean	logFC Mean		logFC Mean	-
NATMI	Specificity-based edge weight		Specificity-based edge weight	Mean-expression edge weight
SingleCellSignalR	LRscore		LRscore	LRscore

Hashtag (#): Unless explicitly mentioned, we used the composite method settings.

Supplementary table 4. Signalling category examples.

Signalling type	Transmitter	Transmitter symbol	Receiver	Receiver symbol	Resource
Direct-contact	Q9NQS3	NECTIN3	Q495A1	TIGIT	Cellinker
Direct-contact	P52803	EFNA5	P54764	EPHA4	Ramilowski
Direct-contact	Q9NT99	LRRC4B	Q6UXZ4	UNC5D	Cellinker
Direct-contact	P10321	HLA-C	P43632	KIR2DS4	Ramilowski
Direct-contact	Q14952	KIR2DS3	Q7Z3B1	NEGR1	Cellinker
Other	P21741	MDK	P18827	SDC1	Cellinker
Other	P01374	LTA	P19438	TNFRSF1A	OmniPath
Other	P21741	MDK	P18827	SDC1	CellChatDB
Other	P49763	PGF	P17948	FLT1	EMBRACE
Other	P06858	LPL	P13611	VCAN	Cellinker
Secreted	Q8IZJ0	IFNL2	Q08334	IL10RB	Ramilowski
Secreted	P00747	PLG	P05556	ITGB1	iTALK
Secreted	P56706	WNT7B	O75197	LRP5	Ramilowski
Secreted	P20062	TCN2	P21554	CNR1	iTALK
Secreted	P01303	NPY	Q9Y5X5	NPFFR2	ConnDB2020

Supplementary Note 1. Protein localisation to categorise CCC

To estimate the localisation distributions of transmitters and receivers as well as to categorise CCC interactions according to signalling categories we obtained protein subcellular localisation annotations via OmniPath[19], which combines this information from 20 resources, such as UniProt, the Cell Surface Protein Atlas[20], and Membranome [21]. The localisation annotations were then filtered according to the 51st consensus percentile (**4.1. Methods**), and we used the localisations of transmitters and receivers to approximate the categories of interactions. The largest part of interactions were those between secreted proteins targeting transmembrane proteins (Secreted -> Transmembrane), which we refer to as the 'Secreted signalling' category. Further, we attributed interactions between and within the transmembrane and peripheral plasma membrane proteins (Transmembrane -> Transmembrane, Peripheral -> Transmembrane, Transmembrane -> Peripheral, Peripheral -> Peripheral) to intercellular signalling events that require physical contact between cells, or 'Direct-contact' signalling. Interactions categorised as neither secreted nor direct-contact were labelled as 'Other' and made up the remainder of the interactions. These were largely a consequence of the localisation annotation process, i.e. such involving proteins matched to multiple localisation categories; e.g. proteins annotated as both secreted and peripheral/transmembrane (**Supp. table 4**).

On average 77% of transmitters and 89% of receivers were annotated as secreted and transmembrane proteins, respectively (**Supp. Fig. S6E-F**). CCC resources were predominantly (73% on average) composed of interactions associated with secreted signalling, while direct-contact signalling constituted a substantially smaller (18% on average) proportion of interactions (**Supp. Fig. S6G**). The proportions of secreted and direct-contact signalling varied between resources, as some of them, such as Cellinker and OmniPath had an overrepresentation of direct-contact signalling when compared to the collective, while the opposite was noted for Guide to Pharmacology and Kirouac2010 (**Supp. Fig. S6A**).

Our results suggest that localisations of transmitters and receivers were largely uniformly distributed and that secreted signalling was predominant across all resources. Yet, differences were noted between the relative abundance of secreted and direct-contact signalling interactions.

Supplementary Note 2. Examining the Disagreement in CCC predictions

Since methods report the different total numbers of interactions, we also assessed the agreement by comparing the top 1% predicted interactions, and found low overlap between the different combinations. In this case, the results generated using different methods showed markedly lower overlap than those generated using different resources (**Supp. Fig. S12**). We then merged and clustered the results from all of the datasets, and found that highest ranked interactions were partly grouped by method (**Supp. Fig. S16-17**). There was nevertheless some, albeit limited, similarity between the predictions of CellChat, CellPhoneDB, and SingleCellSignalR; NATMI and Crosstalk scores; as well as between logFC Mean and Connectome (**Supp. Fig. S16-17**). Results were broadly comparable when considering only the p-values for the ranking of CellChat and CellPhoneDB to compare with the other methods (**Supp. Fig. S18-19; Supp. table 3**).

To better explain the low overlap between the methods, we first hypothesised that the observed discrepancies stem from the method's distinct approaches to prioritise the interactions, and in particular the approaches used to assign cell type specificity to the interactions. For methods that provide additional alternative non-specific scoring functions, we used those functions which consider the magnitude of expression alone to estimate the CCC predictions (**Supp. table 3**). In this case, the overlap between methods was substantially higher, ranging from 0.519 to 0.569 (median = 0.543), while the low overlap when using different resources remained relatively unchanged and ranged from 0.034 to 0.116 (median = 0.075) (**Supp. Fig. S20**) and there was a clear clustering by the different resources (**Supp. Fig. S21**).

We thus postulated that the low overlap between the methods when using their recommended scoring functions was explained by differences at the cell type level. As such, we calculated the frequencies of interactions per cell type, and saw grouping largely by method (**Supp. Fig. S22**). We performed the same analysis using the alternative, non-specific scoring functions and instead noted clustering by resource, while predictions from methods were generally grouped (**Supp. Fig. S23**). We also estimated the relative interaction strength assigned to

each cell type (4.5 Methods), and in this case we noted clustering by method for both the specific and non-specific interaction scores (**Supp. Fig. S24-25**).

The remainder of the differences between the top predictions across the different methods are expected to arise from the methods' distinct approaches in the preprocessing of the input data or from the fact that some methods consider multi-complex subunits whereas others omit such information.

Taken together, these results suggest that the low overlap between the methods when using their recommended scoring functions, is largely a product of the distinct approaches to compute the cell-type specificity aspect of the CCC interactions scores. Finally, this was also reflected by the relative importances assigned to the different cell types.

Supplementary Note 3. Agreement with Receptor Protein specificity

To assess the agreement between CCC predictions from transcriptomics data and antibody-tagged receptor protein abundance, we used seven CITE-Seq datasets (Methods 4.8), which had between 9 and 106 surface-protein genes matched to receptor proteins. In particular, we assessed the agreement of CCC methods with receptor protein specificity by estimating their area under the receiver-operator characteristic (AUROC) and area under the precision-recall curve (AUPRC) metrics (Methods 4.6.3).

We first built AUROC and AUPRC curves for each method using their independent universes, meaning that each method's performance was estimated using only the interactions returned by that method. While the methods' performance is not directly comparable, this allowed us to independently assess the performance of the methods as originally proposed.

All methods were better than random at predicting the most specifically expressed receptor proteins (**Supp. Fig. S26A**), including SingleCellSignalR, which does not explicitly take specificity into account. As anticipated, CellPhoneDB and CellChat showed markedly better performance when we used their p-values alone to predict receptor specificity instead of their composite scores (**Supp. table 3**; **Supp. Fig. S26B**).

Albeit one could assume that receptor protein abundance is possibly more informative about the occurring CCC events than RNA expression alone, the observed agreement of all methods with receptor specificity is itself largely expected due to the correlation between RNA and protein expression. Nevertheless, our analysis suggests that estimates of CCC events using RNA information are also expected to be a good proxy of predictions at the protein level.

Bibliography

1. Baccin, C. *et al.* Combined single-cell and spatial transcriptomics reveal the molecular, cellular and spatial bone marrow niche organization. *Nat. Cell Biol.* **22**, 38–48 (2020).
2. Zhang, Y. *et al.* CellCall: integrating paired ligand-receptor and transcription factor activities for cell-cell communication. *Nucleic Acids Res.* **49**, 8520–8534 (2021).
3. Jin, S. *et al.* Inference and analysis of cell-cell communication using CellChat. *Nat. Commun.* **12**, 1088 (2021).
4. Zhang, Y. *et al.* Cellinker: a platform of ligand-receptor interactions for intercellular communication analysis. *Bioinformatics* (2021) doi:10.1093/bioinformatics/btab036.
5. Garcia-Alonso, L. *et al.* Mapping the temporal and spatial dynamics of the human endometrium in vivo and in vitro. *BioRxiv* (2021) doi:10.1101/2021.01.02.425073.
6. Efremova, M., Vento-Tormo, M., Teichmann, S. A. & Vento-Tormo, R. CellPhoneDB: inferring cell-cell communication from combined expression of multi-subunit ligand-receptor complexes. *Nat. Protoc.* **15**, 1484–1506 (2020).
7. Vento-Tormo, R. *et al.* Single-cell reconstruction of the early maternal-fetal interface in humans. *Nature* **563**, 347–353 (2018).
8. Shao, X. *et al.* CellTalkDB: a manually curated database of ligand-receptor interactions in humans and mice. *Brief. Bioinformatics* **22**, (2021).
9. Hou, R., Denisenko, E., Ong, H. T., Ramilowski, J. A. & Forrest, A. R. R. Predicting cell-to-cell communication networks using NATMI. *Nat. Commun.* **11**, 5011 (2020).
10. Sheikh, B. N. *et al.* Systematic Identification of Cell-Cell Communication Networks in the Developing Brain. *iScience* **21**, 273–287 (2019).
11. Harding, S. D. *et al.* The IUPHAR/BPS Guide to PHARMACOLOGY in 2018: updates and expansion to encompass the new guide to IMMUNOPHARMACOLOGY. *Nucleic Acids Res.* **46**, D1091–D1106 (2018).
12. Ben-Shlomo, I., Yu Hsu, S., Rauch, R., Kowalski, H. W. & Hsueh, A. J. W. Signaling receptome: a genomic and evolutionary perspective of plasma membrane receptors involved in signal transduction. *Sci. STKE* **2003**, RE9 (2003).

13. Noël, F. *et al.* Dissection of intercellular communication using the transcriptome-based framework ICELLNET. *Nat. Commun.* **12**, 1089 (2021).
14. Wang, Y. *et al.* iTALK: an R Package to Characterize and Illustrate Intercellular Communication. *BioRxiv* (2019) doi:10.1101/507871.
15. Kirouac, D. C. *et al.* Dynamic interaction networks in a hierarchically organized tissue. *Mol. Syst. Biol.* **6**, 417 (2010).
16. Cabello-Aguilar, S. *et al.* SingleCellSignalR: inference of intercellular networks from single-cell transcriptomics. *Nucleic Acids Res.* **48**, e55 (2020).
17. Ramilowski, J. A. *et al.* A draft network of ligand-receptor-mediated multicellular signalling in human. *Nat. Commun.* **6**, 7866 (2015).
18. Jakobsson, J. E. T., Spjuth, O. & Lagerström, M. C. scConnect: a method for exploratory analysis of cell-cell communication based on single cell RNA sequencing data. *Bioinformatics* (2021) doi:10.1093/bioinformatics/btab245.
19. Türei, D. *et al.* Integrated intra- and intercellular signaling knowledge for multicellular omics analysis. *Mol. Syst. Biol.* **17**, e9923 (2021).
20. Bausch-Fluck, D. *et al.* A mass spectrometric-derived cell surface protein atlas. *PLoS ONE* **10**, e0121314 (2015).
21. Lomize, A. L., Lomize, M. A., Krolicki, S. R. & Pogozheva, I. D. Membranome: a database for proteome-wide analysis of single-pass membrane proteins. *Nucleic Acids Res.* **45**, D250–D255 (2017).
