# Supplementary material for: Comparison of methods and resources for cell-cell communication inference from single-cell RNA-Seq data
Source: Nat Commun. 2022 Jun 9;13:3224. doi: 10.1038/s41467-022-30755-0 (PMC9184522; doi:10.1038/s41467-022-30755-0)
Supplement: Supplementary file 2 — Reporting Summary [file 41467_2022_30755_MOESM2_ESM.pdf]

nature portfolio

Corresponding author(s): Julio Saez Rodriguez

Last updated by author(s): May 9, 2022

Reporting Summary

Nature Portfolio wishes to improve the reproducibility of the work that we publish. This form provides structure for consistency and transparency in reporting. For further information on Nature Portfolio policies, see our Editorial Policies and the Editorial Policy Checklist.

Statistics

For all statistical analyses, confirm that the following items are present in the figure legend, table legend, main text, or Methods section.

n/a	Confirmed	
☒	☐	The exact sample size (*n*) for each experimental group/condition, given as a discrete number and unit of measurement
☐	☒	A statement on whether measurements were taken from distinct samples or whether the same sample was measured repeatedly
☐	☒	The statistical test(s) used AND whether they are one- or two-sided<br>*Only common tests should be described solely by name; describe more complex techniques in the Methods section.*
☐	☒	A description of all covariates tested
☐	☒	A description of any assumptions or corrections, such as tests of normality and adjustment for multiple comparisons
☐	☒	A full description of the statistical parameters including central tendency (e.g. means) or other basic estimates (e.g. regression coefficient) AND variation (e.g. standard deviation) or associated estimates of uncertainty (e.g. confidence intervals)
☐	☒	For null hypothesis testing, the test statistic (e.g. *F*, *t*, *r*) with confidence intervals, effect sizes, degrees of freedom and *P* value noted<br>*Give P values as exact values whenever suitable.*
☒	☐	For Bayesian analysis, information on the choice of priors and Markov chain Monte Carlo settings
☒	☐	For hierarchical and complex designs, identification of the appropriate level for tests and full reporting of outcomes
☐	☒	Estimates of effect sizes (e.g. Cohen's *d*, Pearson's *r*), indicating how they were calculated

Our web collection on statistics for biologists contains articles on many of the points above.

Software and code

Policy information about availability of computer code

Data collection	SeuratData_0.2.1, Squidpy1.1.2, OmnipathR_3.3.3
Data analysis	The work presented in this work was generated using liana_0.0.5 (https://github.com/saezlab/liana). All relevant code to reproduce the analyses is available at:https://github.com/saezlab/ligrec_decouple.<br><br>Relevant software:<br>decoupleR_2.1.3, rlang_0.4.11, logger_0.2.0, shadowtext_0.0.8, RCurl_1.98-1.5, viridis_0.6.1, viridisLite_0.4.0, OmnipathR_3.3.3, ggplotify_0.1.0, UpSetR_1.4.0, proxy_0.4-26, pheatmap_1.0.12, RColorBrewer_1.1.2, patchwork_1.1.1, ggsignif_0.6.3, ComplexHeatmap_2.6.2, org.Hs.eg.db_3.12.0, org.Mm.eg.db_3.12.0, BiocManager_1.30.16, Connectome_1.0.1, SingleCellExperiment_1.12.0, biomaRt_2.46.3, SCAomni_0.0.1.8* (https://github.com/saezlab/SingleCellSignalR_v1), SummarizedExperiment_1.20.0, GenomicRanges_1.42.0, GenomeInfoDb_1.26.7, IRanges_2.24.1, S4Vectors_0.28.1, NATMI* (https://github.com/saezlab/NATMI), CellChat_1.1.3, BiocGenerics_0.36.1, igraph_1.2.6 yardstick_0.0.8 , SeuratObject_4.0.2, Seurat_4.0.3, forcats_0.5.1, stringr_1.4.0, dplyr_1.0.6, purrr_0.3.4, readr_2.1.0, tidyr_1.1.3, tibble_3.1.2, ggplot2_3.3.5, tidyverse_1.3.1, magrittr_2.0.1, Squidpy1.1.2

For manuscripts utilizing custom algorithms or software that are central to the research but not yet described in published literature, software must be made available to editors and reviewers. We strongly encourage code deposition in a community repository (e.g. GitHub). See the Nature Portfolio guidelines for submitting code & software for further information.

Data

Policy information about availability of data

All manuscripts must include a data availability statement. This statement should provide the following information, where applicable:

- Accession codes, unique identifiers, or web links for publicly available datasets
- A description of any restrictions on data availability
- For clinical datasets or third party data, please ensure that the statement adheres to our policy

The processed and annotated Human Breast Cancer single-cell atlas 43 is available via the GEO accession number: GSE176078 [https://www.ncbi.nlm.nih.gov/geo/query/acc.cgi?acc=GSE176078]. The filtered breast cancer 10x Visium slides from the same publication are available at https://zenodo.org/record/4739739.
Processed seqFISH 77 [https://content.cruk.cam.ac.uk/jmlab/SpatialMouseAtlas2020/] and merFISH 52 (GEO accession number: GSE113576 [https://www.ncbi.nlm.nih.gov/geo/query/acc.cgi?acc=GSE113576]) datasets were obtained via the spatial single-cell analysis framework - Squidpy (v1.1.0) 69 [https://squidpy.readthedocs.io/en/latest/api.html#module-squidpy.datasets].
Pancreatic islet 45 (GEO accession numbers: GSE84133 [http://www.ncbi.nlm.nih.gov/geo/query/acc.cgi?acc=GSE84133], GSE81076 [http://www.ncbi.nlm.nih.gov/geo/query/acc.cgi?acc=GSE81076], GSE85241 [http://www.ncbi.nlm.nih.gov/geo/query/acc.cgi?acc=GSE85241], GSE86469 [http://www.ncbi.nlm.nih.gov/geo/query/acc.cgi?acc=GSE86469]; ArrayExpress: E-MTAB-5061 [http://www.ebi.ac.uk/microarray-as/aer/result?queryFor=Experiment&eAccession=E-MTAB-5061]) and cord blood mononuclear cells 44 (GEO accession number: GSE100866 [https://www.ncbi.nlm.nih.gov/geo/query/acc.cgi?acc=GSE100866]) scRNA-Seq datasets were obtained via SeuratData (https://github.com/satijalab/seurat-data).
Publicly available 5K PBMC [https://support.10xgenomics.com/single-cell-gene-expression/datasets/3.1.0/5k_pbmc_protein_v3_nextgem], 5K PBMC NextGem [https://support.10xgenomics.com/single-cell-gene-expression/datasets/3.1.0/5k_pbmc_protein_v3], 10K PBCM [https://support.10xgenomics.com/single-cell-gene-expression/datasets/3.1.0/5k_pbmc_protein_v3], and 10K MALT (https://www.10xgenomics.com/resources/datasets/10-k-cells-from-a-malt-tumor-gene-expression-and-cell-surface-protein-3-standard-3-0-0) CITE-Seq datasets were obtained from 10X Genomics (accessible under the list of datasets at https://tinyurl.com/10xCITEseq).
Processed and annotated murine spleen-lymph CITE-Seq datasets 78 are available via the GEO accession number: GSE150599 [https://www.ncbi.nlm.nih.gov/geo/query/acc.cgi?acc=GSE150599].
The processed single cell RNA-Seq data 46 for 23 Korean colorectal cancer patients are available via the GEO accession number: GSE132465 [https://www.ncbi.nlm.nih.gov/geo/query/acc.cgi?acc=GSE132465].
Spatial transcriptomics datasets (10x Visium slides) on sagittal adult mouse brain anterior and posterior slices were obtained from SeuratData, available at https://github.com/satijalab/seurat-data, under the dataset name of `stxBrain`, or publically via the 10X Genomics website under Spatial Gene Expression v1 Chemistry datasets [https://tinyurl.com/10xVisiumDemonstration]. The single-cell data (Allen Brain Atlas 50) used for the cell type mapping (deconvolution), was obtained as a Seurat object, accessible at https://www.dropbox.com/s/cuowvm4vrf65pvq/allen_cortex.rds?dl=1, and is alternatively available via accession number: GSE71585 [http://www.ncbi.nlm.nih.gov/geo/query/acc.cgi?acc=GSE71585].
The 10x Genomics' 3k PBMC dataset used in the robustness analysis is available at https://cf.10xgenomics.com/samples/cell/pbmc3k/pbmc3k_filtered_gene_bc_matrices.tar.gz. Source data are provided with this paper.

Field-specific reporting

Please select the one below that is the best fit for your research. If you are not sure, read the appropriate sections before making your selection.

☒ Life sciences ☐ Behavioural & social sciences ☐ Ecological, evolutionary & environmental sciences

For a reference copy of the document with all sections, see nature.com/documents/nr-reporting-summary-flat.pdf

Life sciences study design

All studies must disclose on these points even when the disclosure is negative.

Sample size	No sample size calculation was performed. All data presented in this manuscript were previously published and used to compare the methods. The choice of dataset was mainly driven by the quality and ease of availability of the data. The number and wide range of datasets types was sufficient to support the results presented in this work.
Data exclusions	No data was excluded from the work presented here. The only exceptions were standard and pre-established quality control steps of single-cell RNA-seq data, such as removal of low quality cells and lowly expressed genes.
Replication	All replication attempts were consistent and can be performed independently
Randomization	Allocation was random.
Blinding	All results are based on published data. Therefore, blinding from investigators is not applicable.

Reporting for specific materials, systems and methods

We require information from authors about some types of materials, experimental systems and methods used in many studies. Here, indicate whether each material, system or method listed is relevant to your study. If you are not sure if a list item applies to your research, read the appropriate section before selecting a response.

Materials & experimental systems

n/a	Involved in the study
☒	☐ Antibodies
☒	☐ Eukaryotic cell lines
☒	☐ Palaeontology and archaeology
☒	☐ Animals and other organisms
☒	☐ Human research participants
☒	☐ Clinical data
☒	☐ Dual use research of concern

Methods

n/a	Involved in the study
☒	☐ ChIP-seq
☒	☐ Flow cytometry
☒	☐ MRI-based neuroimaging
